# The contemporary model of vertebral column joint dysfunction and impact of high-velocity, low-amplitude controlled vertebral thrusts on neuromuscular function

**DOI:** 10.1007/s00421-021-04727-z

**Published:** 2021-06-23

**Authors:** Heidi Haavik, Nitika Kumari, Kelly Holt, Imran Khan Niazi, Imran Amjad, Amit N. Pujari, Kemal Sitki Türker, Bernadette Murphy

**Affiliations:** 1 Centre for Chiropractic Research, New Zealand College of Chiropractic, Auckland, New Zealand; 2 Faculty of Health and Environmental Sciences, Health and Rehabilitation Research Institute, AUT University, Auckland, New Zealand; 3 Department of Health Science and Technology, Aalborg University, Aalborg, Denmark; 4 Riphah International University, Islamabad, Pakistan; 5 School of Physics, Engineering and Computer Science, University of Hertfordshire, Hatfield, UK; 6 School of Engineering, University of Aberdeen, Aberdeen, UK; 7 School of Medicine, Koç University, Istanbul, Turkey; 8 Faculty of Dentistry, Gelişim University, Istanbul, Turkey; 9 Faculty of Health Sciences, University of Ontario Institute of Technology, Oshawa, ON Canada

**Keywords:** Chiropractic, Spinal manipulation, Muscle strength, Neuromuscular function

## Abstract

**Purpose:**

There is growing evidence that vertebral column function and dysfunction play a vital role in neuromuscular control. This invited review summarises the evidence about how vertebral column dysfunction, known as a central segmental motor control (CSMC) problem, alters neuromuscular function and how spinal adjustments (high-velocity, low-amplitude or HVLA thrusts directed at a CSMC problem) and spinal manipulation (HVLA thrusts directed at segments of the vertebral column that may not have clinical indicators of a CSMC problem) alters neuromuscular function.

**Methods:**

The current review elucidates the peripheral mechanisms by which CSMC problems, the spinal adjustment or spinal manipulation alter the afferent input from the paravertebral tissues. It summarises the contemporary model that provides a biologically plausible explanation for CSMC problems, the manipulable spinal lesion. This review also summarises the contemporary, biologically plausible understanding about how spinal adjustments enable more efficient production of muscular force. The evidence showing how spinal dysfunction, spinal manipulation and spinal adjustments alter central multimodal integration and motor control centres will be covered in a second invited review.

**Results:**

Many studies have shown spinal adjustments increase voluntary force and prevent fatigue, which mainly occurs due to altered supraspinal excitability and multimodal integration. The literature suggests physical injury, pain, inflammation, and acute or chronic physiological or psychological stress can alter the vertebral column’s central neural motor control, leading to a CSMC problem. The many gaps in the literature have been identified, along with suggestions for future studies.

**Conclusion:**

Spinal adjustments of CSMC problems impact motor control in a variety of ways. These include increasing muscle force and preventing fatigue. These changes in neuromuscular function most likely occur due to changes in supraspinal excitability. The current contemporary model of the CSMC problem, and our understanding of the mechanisms of spinal adjustments, provide a biologically plausible explanation for how the vertebral column’s central neural motor control can dysfunction, can lead to a self-perpetuating central segmental motor control problem, and how HVLA spinal adjustments can improve neuromuscular function.

## Introduction

The vertebral column is linked biomechanically and neurologically to the limbs. Yet, we know very little about how altered sensory feedback from the vertebral column affects limb sensorimotor integration and motor performance. Recently, several research studies have documented changes in motor output following vertebral column dysfunction or perturbations involving the application of controlled vertebral column high-velocity, low-amplitude (HVLA) thrusts (Christiansen et al. 2018; Farid et al. 2018; Haavik and Murphy 2011; Haavik et al. 2017, 2018a, b; Haavik Taylor and Murphy 2008; Haavik-Taylor and Murphy 2007a; Holt et al. 2016a, b, 2019; Lelic et al. 2016; Niazi et al. 2015). The mechanisms for these changes are still not fully understood. With this invited review, the current understanding of how vertebral column motion segment movement and perturbations to the vertebral column with HVLA vertebral column thrusts will be discussed. Throughout this review, the part of the spine identified as the site of biomechanical dysfunction and thus, the clinical target of an HVLA thrust, will be referred to as a central segmental motor control (CSMC) problem. This review will focus on what is known about the physiology of spinal joint dysfunction, including CSMC problems. CSMC problems, are by some referred to as vertebral subluxations (Cooperstein 2010, 2013; Holt et al. 2019; Niazi et al. 2015). Vertebral subluxation is a term recognised as biomechanical lesions of the vertebral column by the World Health Organization (Organization 2005), is recognised in the International Statistical Classification of Diseases and Related Health Problems (ICD) (ICD-10-CM code M99.1), and is used in many research publications (Cooperstein et al. 2010, 2013; Holt et al. 2019; Niazi et al. 2015). The basic science research that has emerged over the past two decades has led to vertebral subluxations being characterised as self-perpetuating, central segmental motor control (CSMC) problems that involve a joint, such as a vertebral motion segment, that is not moving appropriately, resulting in ongoing maladaptive neural plastic changes that interfere with the central nervous system’s (CNS’s) ability to regulate neuromuscular function (Cooperstein et al. 2013; Gatterman 1995; WHO 2016). It is thought that such “maladaptation” of body posture may initially be beneficial and potentially occurs to avoid further pain from the region (pain adaptation concept of (Lund et al. 1991), however when maintained for a long period of time, this response may become maladaptive or harmful.

A CSMC problem is characterised by tight vertebral muscles, reduced intervertebral movement and tenderness to touch (Triano et al. 2013). The clinical importance of this type of vertebral dysfunction is considered not only important by chiropractors, but also various other health professionals, such as osteopaths who call it ‘somatic dysfunction’ or ‘spinal lesion’ and physiotherapists and physical medicine specialists who use the term ‘vertebral (spinal) lesion’ (Leach 1986). Within the chiropractic profession, this spinal lesion has been called by many names over the years, including ‘manipulable or functional spinal lesion’, ‘vertebral subluxation complex’, ‘chiropractic subluxation’, ‘subluxation’, ‘vertebral subluxation’, ‘biomechanical joint dysfunction’, or ‘spinal fixation’ (Nelson 1997; Triano et al. 2013; Ebrall et al. 2008; Gatterman 1995; The Rubicon Group 2017).

The CSMC problems can be identified using a combination of pathophysiologic indicators of vertebral column dysfunction (Triano et al. 2013) and then corrected using a variety of manual techniques (Cooperstein and Gleberzon 2004). The most common technique is a specific HVLA thrust directed at a motion segment with a CSMC problem, also known as an adjustment (Coulter and Shekelle 2005). It is possible to direct a thrust at any spinal segment, regardless of whether it is dysfunctional or not. Therefore, for the purposes of this review, if a thrust is directed at a spinal segment that has not been examined and identified as having clinical indicators of dysfunction, it will be referred to as spinal manipulation. In contrast, a thrust directed at a dysfunctional vertebral motion segment will be referred to as a spinal adjustment or simply adjustment. This distinction is important, as adjustments are likely to have different physiological consequences compared to thrusting at or manipulating a vertebral segment that has no signs of motor control dysfunction, and may explain contradictory findings in the literature. The evidence for central neuroplastic effects of spinal adjustments and spinal manipulation will be considered and discussed in relation to known factors that influence motor output. Gaps in the literature will be identified. In this first invited review, the current contemporary understanding of the mechanisms by which CSMC problems arise, and the known neuroplastic neuromuscular consequences of spinal adjustments or spinal manipulation will be discussed. The direct evidence that exists showing spinal adjustments or manipulations alter neuromuscular function will also be discussed. Then each peripheral receptor that could be involved in conveying the altered sensory feedback from the areas of the vertebral column with evidence of motor control dysfunction will be considered, and any evidence, to date, that has shown how spinal adjustments or spinal manipulation can impact the signalling of these sensory organs will be discussed. The second invited review will summarise the evidence for changes in the spinal or supraspinal motor control centres following spinal adjustments and spinal manipulation. Where appropriate, the findings from various experiments that have investigated the consequences of altered spinal afferent input to the central nervous system (CNS), including both acute models (such as fatigue or injury) and chronic models (such as subclinical pain) will be discussed to help elucidate how vertebral column afferent input ultimately influences neuromuscular control and function. The previously published review by Haavik and Murphy (2012) on the role of spinal manipulation in disordered sensorimotor integration will be updated with recent evidence of the impact of spinal manipulation on multisensory integration. This review has great relevance to understanding the role of vertebral column function and dysfunction and the physiological consequences of spinal adjustments or manipulations on neuromuscular control for multiple clinical populations, including those with recurrent and chronic spinal pain, athletes and right through to populations that have lost some of their ability to voluntarily activate their muscles, such as chronic stroke populations.

## Methods

PubMed, CINHAL and Google Scholar were searched for relevant articles through to December 2020 to inform this review. The search strategy for reviewing the effect of spinal adjustments or spinal manipulation on strength included the following search terms: chiropractic, manual therapy, HVLA, adjustment, manipulation, strength, maximum voluntary contraction, electromyography (EMG), and motor-evoked potential. Specific inclusion criteria were: spinal adjustments or spinal manipulation were the intervention assessed and muscle output or force were measured as an outcome. The search strategy for reviewing the effects of spinal adjustments or spinal manipulation on sensory organs included the following search terms: chiropractic, manual therapy, HVLA, adjustment, manipulation, muscle spindle and Golgi tendon organ. Overall, studies were included if they met all the following criteria: spinal adjustments or spinal manipulation where the intervention assessed and the study appeared in a peer-reviewed English-language journal. Studies were excluded if they were reviews, books, theses, conference papers, commentaries, or letters. The reference list of included studies and recent systematic reviews were also searched.

### Overview of the contemporary understanding of the mechanisms by which central segmental motor control (CSMC) problems, spinal adjustments or spinal manipulation impact neuromuscular function

Movement control relies on the accurate detection and integration of multiple sensory receptor inputs from the inside (interoception) and outside the body (exteroception). Interoception is the perception of internal bodily signals and processes (Craig 2002, 2003; Craig and Craig 2009; Critchley and Garfinkel 2017; Quadt et al. 2018; Chen et al. 2021) and includes proprioception (sense of the position of the limbs against the trunk), vestibular sense or equilibrium (sense of the position of the body against the gravity), vasomotor flushing (e.g., hot flashes), immune activity, autonomic activity, thirst, and distension of the bladder, stomach, rectum or oesophagus (Craig 2002, 2003; Craig and Craig 2009). Exteroception is the perception of external, environmental stimuli, such as visual, auditory, touch, smell and taste stimuli (Blanke et al. 2015; Kassab and Alexandre 2015). When planning a movement, this sensory information is integrated with memories and the current movement goal to send appropriate motor commands in the correct order and at the precise time needed, to perform the intended movements optimally. Various anticipatory and postural control mechanisms also come into play to enable the accurate execution of this intended movement. All this occurs while the actual sensory feedback of the movement is compared with expected feedback and efference copies (copies of the movement commands that the brain sends out to muscles) to fine-tune the movement in progress (Tagliabue and McIntyre 2014). The efference copies also play a role in inner body and external world schemas, sensorimotor integration and motor control (Kilteni et al. 2019).

**Fig. 1:**
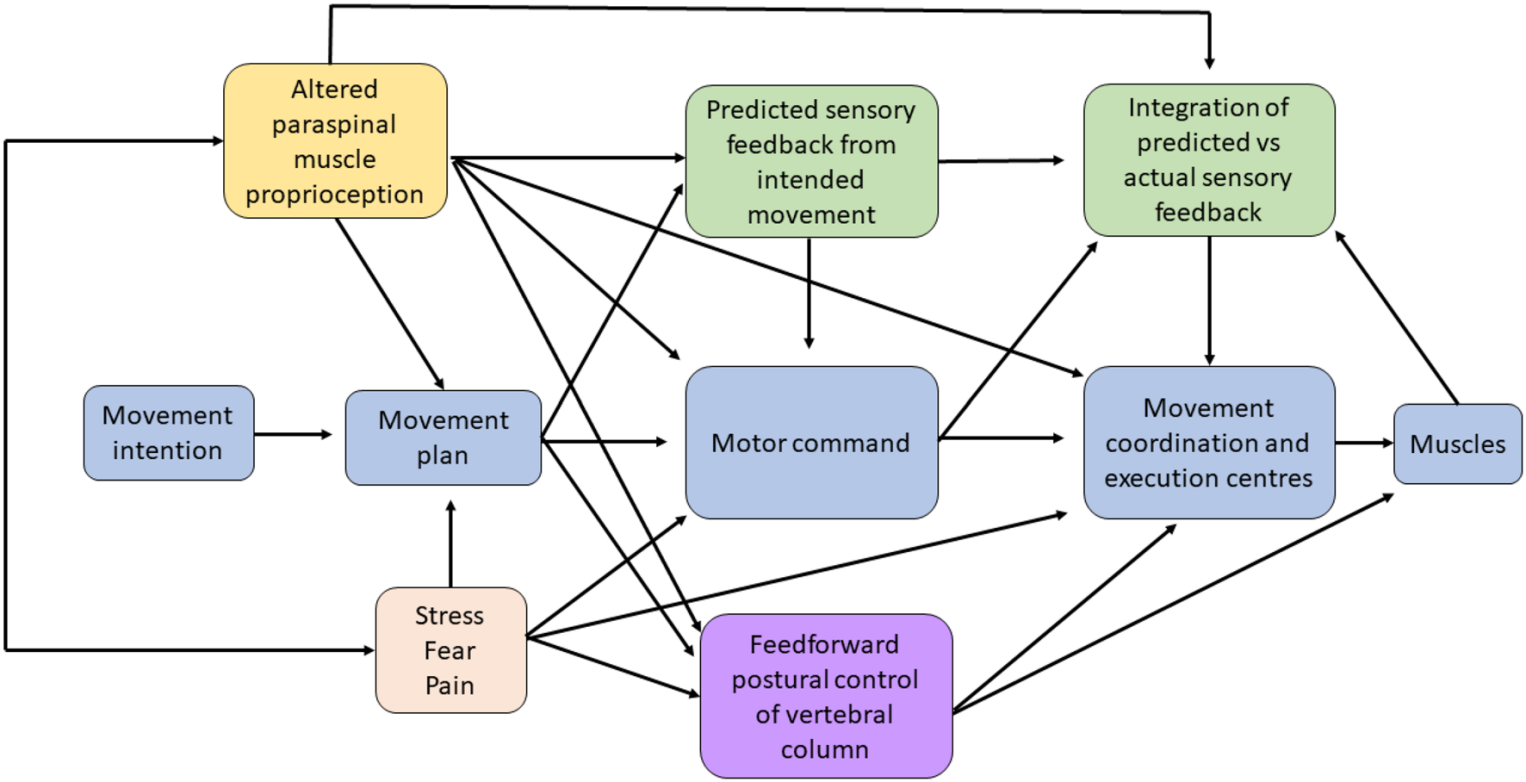
Image depicting the sensorimotor integration (SMI) that occurs during the performance of a movement. It specifically depicts how altered paraspinal muscle proprioceptive input from either a central segmental motor control (CSMC) problem or from an adjustment (yellow box) likely alters neuromuscular function at multiple levels, by impacting the motor plan itself, the motor command messages, the predicted sensory feedback the CNS will expect and therefore the integration of the predicted and actual sensory feedback created by the moving muscles as well as feedforward postural control of the vertebral column. Additional situations and conditions, such as stress, fear or the presence of pain (orange box) are also known to influence multiple aspects of the movement. There is also some evidence that altered paraspinal muscle proprioceptive input can influence and is influenced by stress, pain and fear. The evidence for how altered paraspinal muscle proprioceptive input from CSMC problems or joint dysfunction animal models influences any of these aspects of a movement is discussed in greater detail throughout this invited review

Figure 1 highlights the impact of deep proprioceptive afferent information from paraspinal muscles to this process of performing a movement. The evidence to support Fig. 1, specifically the role of altered paraspinal proprioceptive input from CSMC problems or spinal adjustments and/or manipulations is discussed in several of the following sections of this review.

The basic science research about CSMC problems and mechanisms of spinal adjustments and manipulations has seen a shift away from a local structural pathology model, where a CSMC problem was thought to directly put pressure on, or irritate, spinal nerve roots or the spinal cord itself (Grostic 1988; Stephensen 1927), towards a more central neuroplasticity model (Boal and Gillette 2004; Gyer et al. 2019; Haavik and Murphy 2012; Hennenhoefer and Schmidt 2019; Pickar 2002; Haavik Taylor et al. 2010). It is well documented that there can be both spinal cord and intervertebral foramina (IVF) encroachment due to overt pathology, such as tumours or disc herniations. Moreover, since it is possible that such spinal canal and IVF encroachment can be asymptomatic (Borenstein et al. 2001), it was thought that maybe CSMC problems directly “squashed” nerve roots, interfering with action potential transmission or axoplasmic flow across that nerve root, thus interfering with the structures that nerve root innervated. Human studies have shown that lumbar spine stenosis and disc herniations can encroach on neural tissue enough to retard axoplasmic flow and the latency and amplitude of action potential transmission through the IVF (Morishita et al. 2006). Thus, it is clear that people with a disc herniation definitely can have “squashed” nerve roots to the degree that interferes with nerve conduction through the affected IVF and that people with spinal stenosis can have “squashing” of the spinal cord itself. However, there is no evidence that more subtle vertebral dysfunction, i.e. CSMC problems, have a “squashed” nerve root component. Neither is there any evidence that spinal adjustments or manipulations relieve the pressure of a “squashed” nerve root, except for possibly relieving the pressure of the affected nerve roots of radiculopathy patients (Rodine and Vernon 2012; McMorland et al. 2010). In animal research, one group has shown that compression of the cervical dorsal nerve root can disrupt nerve function if the pressure applied to the nerve root is above 31.6 mN (Hubbard and Winkelstein 2008). Disruption in nerve function was quantified as changes in neurofilament immunoreactivity in the cervical dorsal root. What was interesting though, was that it only required 26.3 mN of pressure to cause mechanical hypersensitivity, an enhanced response to an innocuous stimulus such as touch, in the animals tested (Hubbard and Winkelstein 2008; Lolignier et al. 2015). In this study, the C6/7 nerve root was exposed in anaesthetised rats, and then the pressure on the C7 nerve root was slowly increased for 15 min (Hubbard and Winkelstein 2008). The amount of pressure applied to different rat’s nerve roots could be varied and then the impact on the rat’s behaviour was observed. The behaviour was assessed by recording the total number of paw withdrawals triggered by stimulating the plantar surface of the right forepaw with a non-nociceptive von Frey filament. This study suggested that the force required to cause axoplasmic flow disruption or action potential conduction changes was greater than the force it took to cause mechanical hypersensitivity in the studied animals. This suggests that the pressure on a nerve root needed to disrupt nerve root communication, would most likely result in radicular symptoms. Most people who have their spine assessed and adjusted by chiropractors do not present with radicular symptoms (Adams et al. 2017); thus it would be fair to suggest that for most people, CSMC problems are unlikely to affect communication across the accompanying nerve roots. Another group has shown in both cats and rabbits that pressure on the dorsal root ganglia (DRG) was required to have long-term effects from nerve root compression (Howe et al. 1977). However, the long-term effect that occurred when the DRG themselves were compressed was a change in sensory afferent feedback to the CNS from the “squashed” nerve root area (Howe et al. 1977). This suggests that to get long-term effects or changes from “squashed” nerve roots, that pressure has to directly impact the DRG. Furthermore, this study shows that the only long-term consequences were afferent changes, not axoplasmic flow changes, nor changes in action potentials across the nerve root itself (Howe et al. 1977). In another study that also explored whether a CSMC problem could include a “squashed” nerve root component, the scientists inserted small steel rods into the IVF’s of rat’s (Song et al. 2003). These authors described their experiment that showed when these small stainless-steel rods were inserted into the rat’s L5 IVF, this mainly caused a hyperafferentation of sensory feedback to the CNS, and they found no evidence of changes to the nerve root function itself (Song et al. 2003). They also showed that these rats ended up exhibiting hind paw hyperalgesia (Song et al. 2003). Combined, these studies suggest that most CSMC problems do not have a “squashed” nerve root component, and instead are most likely to cause altered sensory feedback to the CNS from the dysfunctional vertebral motion segment. This has important implications for both clinicians and scientists. Several of the studies discussed in this review appear to have chosen to apply HVLA thrusts at a part of the spine because this might influence the communication across the nerve roots at the level of thrust application, i.e. ‘relieving pressure off squashed nerve roots’. This might be because these studies were carried out before this research that has shown ‘relieving pressure off squashed nerve roots’ is highly unlikely the mechanisms of an adjustment. This may also be the case in practice, if clinicians are applying HVLA manipulations to certain parts of the spine in an effort to impact the part of the body these nerve roots innervate. Yet there seems to be very little evidence that a CSMC problem interferes with nerve root function, unless the person also has radiating nerve root pain (which would also show up clinically with changes in dermatomes, myotomes and altered stretch reflexes). In experiments, if this faulty reasoning is applied, there may not be any beneficial changes from adjustments, or manipulations, if they have been applied only to regions of the spine that innervate certain structures or muscles of interest. This limitation has been highlighted, where applicable, in the discussions below.

**Fig. 2:**
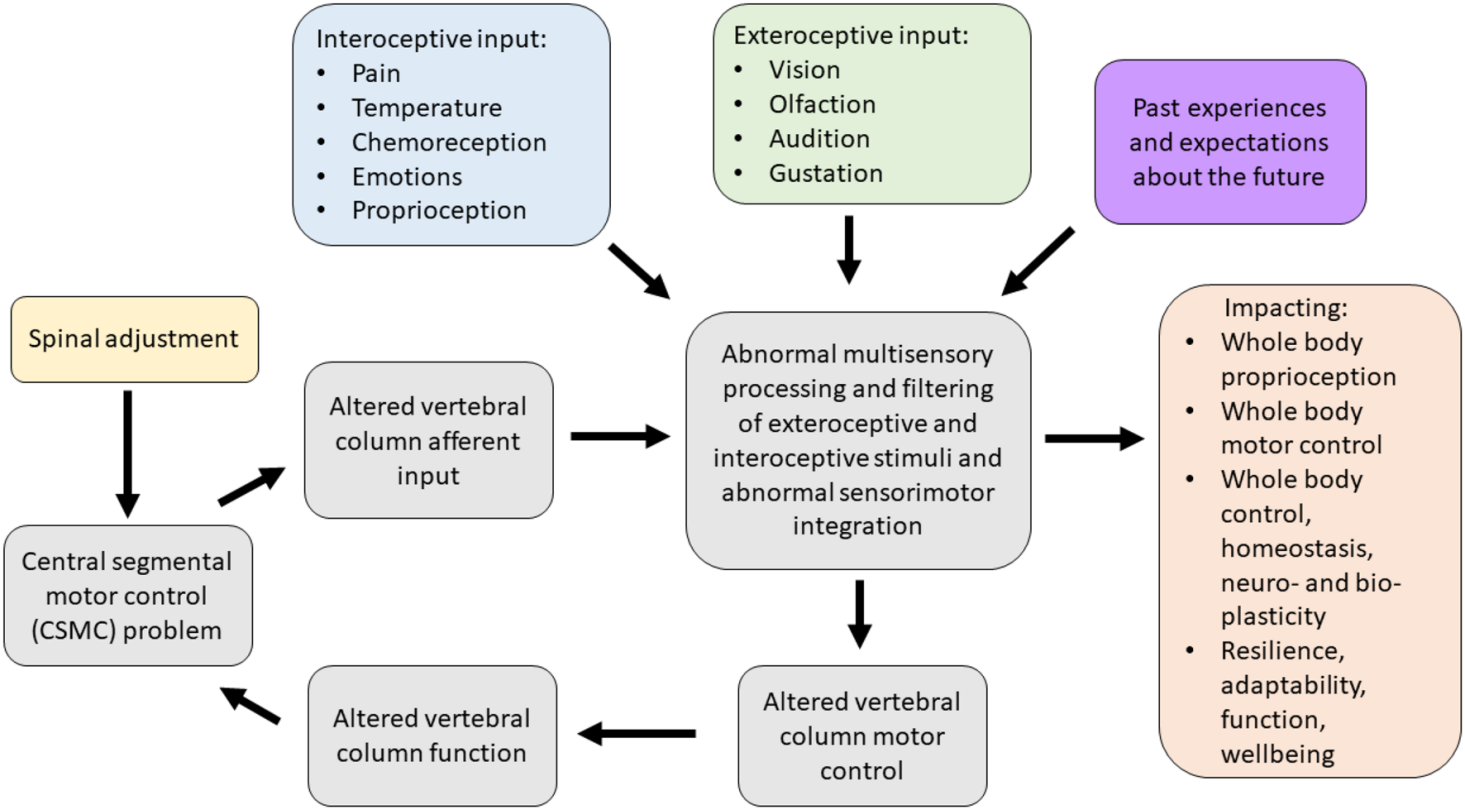
Contemporary model of the mechanism by which central segmental motor control (CSMC) problems and spinal adjustments result in neuroplastic consequences that impact neuromuscular function. The grey boxes, depicting the impact of proprioceptive input from the deep paraspinal muscles on spinal motor control, suggest that vertebral motion segments that have CSMC problems cause altered proprioceptive input, which alters multisensory processing, filtering and integration, along with both interoceptive and exteroceptive stimuli, resulting in abnormal sensorimotor integration of this spinal input. This impacts vertebral column motor control that could alter vertebral column movement/function, causing microtraumas to induce ongoing spinal dysfunction. These grey boxes are therefore seen as a self-perpetuating cycle of abnormal spinal column motor control, that over time, may lead to the development of recurrent and chronic spinal pain. When the spinal HVLA adjustment (yellow box) is applied to a CSMC problem, this may improve proprioceptive input, which in turn improves multisensory interoceptive and exteroceptive integration, thus improving motor control of the vertebral column. The orange box to the right highlights that CSMC problems and adjustments do not just impact the motor control of the spine (i.e. the grey boxes), but also appear to influence motor control of the rest of the body, as well as potentially impacting whole-body awareness, integration, adaptability, function, and wellbeing. The validity of this contemporary model and the degree to which it is supported by the literature is discussed in these invited reviews

The contemporary model of CSMC problems and spinal adjustments (depicted in Fig. 2) therefore now suggests that a CSMC problem can lead to abnormal multisensory processing and filtering of interoceptive and exteroceptive stimuli that can ultimately lead to poor motor control of the vertebral column (grey boxes in Fig. 2) as well as other muscles in the body (orange box of Fig. 2). This can, over time, lead to ongoing maladaptive changes and, with ongoing poor motor control, lead to repeated microtraumas that may ultimately be responsible for the development of musculoskeletal pain syndromes (Meier et al. 2018). This model also explains how spinal adjustments (yellow box in Fig. 2), i.e. HVLA thrusts delivered to segments with a CSMC problem, can improve vertebral column motor control (grey box of Fig. 2) by bombarding the CNS with mechanoreceptive input from the segment with a CSMC problem (Pickar and Wheeler 2001; Sung et al. 2005; Pickar and Kang 2006; Pickar et al. 2007; Cao et al. 2013; Reed et al. 2013a, b, 2014a, b, 2017a, b; Reed and Pickar 2015), yet also impact whole body functions as well (orange box in Fig. 2) (Haavik and Murphy 2011; Holt 2014). An attempt is made to explain how CSMC problems appear to impact motor control of the spine and limbs negatively and how the effects of spinal adjustments appear to improve the motor control of the spine and limbs. There is support in the literature that the proprioceptive input from the deep paraspinal muscles is essential for intervertebral control (MacDonald et al. 2006) (see Fig. 1). It is also known that the activity of deep back muscles is different in people with recurrent unilateral low back pain, despite the resolution of symptoms (MacDonald, Moseley, and Hodges 2009). There has also been a growing number of studies published that supports this contemporary model of CSMC problems, and the mechanisms of spinal adjustments depicted in Fig. 2 (Cramer et al. 2006; Taylor et al. 2010; Haavik and Murphy 2012; Niazi et al. 2015; Christiansen et al. 2018; Niazi et al. 2020; Lelic et al. 2016), all of which will be discussed in these two invited reviews.

The impact of spinal manipulation is also likely to induce a mechanoreceptive blast to the CNS but is unlikely to have the same impact as an adjustment that is directed at a CSMC problem, due to the maladaptive bioplastic changes that are known to occur at the level of a CSMC problem, i.e. at a level of the spine where biomechanical dysfunction exists with accompanying degenerative soft tissue changes. There are, for example, known maladaptive plastic changes in the deep paraspinal muscles following a spinal injury (Brown et al. 2011; Hodges et al. 2006, 2009, 2014, 2015; James et al. 2016). Rapid atrophy due to neural inhibition (Hodges et al. 2006, 2009), the development of muscle fibrosis, extensive fatty infiltration and changes in muscle fibre types (Brown et al. 2011; Hodges et al. 2014, 2015; James et al. 2016; Cooley et al. 2018) have all been found within the deep paraspinal muscles at various time-frames after a spinal injury. The rapid and progressive degeneration of the cervical multifidus muscles has also been found to occur after cervical spine injuries such as whiplash, which include fatty infiltration of these deep paraspinal muscles of the neck (Pedler et al. 2018; Elliott et al. 2015). These local paraspinal muscle changes coincide with ‘smudging’ within the primary sensorimotor cortices (Burns et al. 2016; Chang et al. 2019), and has led scientists to conclude that disrupted or reduced proprioceptive signalling from deep paraspinal muscles likely plays a pivotal role in driving the long-term cortical reorganisation and changes in the top-down control of the sensorimotor systems and that this plays a vital role in driving the recurrence and chronicity of back pain (Meier et al. 2018). Thus, the sensory information from deep paraspinal muscles around a CSMC problem is thought to be the driving factor in the widespread maladaptive neuroplastic changes within the CNS. With such clear evidence that maladaptive dysfunction of the deep paraspinal muscles can occur (Brown et al. 2011; Hodges et al. 2006, 2009, 2014, 2015; James et al. 2016; Elliott et al. 2015; Pedler et al. 2018), which is likely to reduce the ability of the CNS to accurately perceive what is going on at that level of the vertebral column (which over time is reflected by the blurring of the sensorimotor cortical areas Burns et al. 2016; Chang et al. 2019)), this is likely to lead to poor vertebral motor control, maintaining a central segmental motor control problem. Thus, an HVLA thrust directed at a CSMC problem that is surrounded by poorly functioning paraspinal muscles, e.g., following an earlier injury, is likely to have a different physiological response compared to spinal manipulation of a properly functioning vertebral segment with healthy paraspinal muscles and paraspinal tissues. Therefore, for the purposes of this review, careful delineation has been made between publications that have noted in their manuscript that HVLA thrusts were delivered towards a spinal segment with some form of biomechanical dysfunction and studies that have either not provided this evidence or have given other reasons for targeting a spinal segment with their HVLA thrust.

It has been speculated that CSMC problems change the sensory (afferent) input the CNS receives from the small, deep paraspinal muscles of the vertebral column (Alcantara et al. 2013; Haavik and Murphy 2012; Henderson 2012; Kent 1996; Haavik Taylor et al. 2010). This altered vertebral column afferent input appears to modulate the afferent “milieu” into which subsequent afferent feedback from the spine, limbs and other internal and external sensory inputs are acquired and processed. This leads to altered sensorimotor and multimodal integration of the afferent input and changes the accuracy of the inner body and external world schemas (see Figs. 1, 2) (Holt et al. 2016; Haavik Taylor et al. 2010). Over time, these changes in the awareness of the CNS of what is occurring inside the body and the world around it are thought to lead to maladaptive changes in neural function, as well as maladaptive changes in body structure and function, worsening its ability to adapt and respond to internal and environmental cues, thus leading to the development of less than ideal motor control, a variety of symptoms, diseases and disorders (see Figs. 1, 2) (Alcantara et al. 2013; Haavik and Murphy 2012; Kent 1996; Haavik Taylor et al. 2010; Henderson 2012). Adjustments of CSMC problems has been hypothesised to alter the afferent input from the ‘dysfunctional’ small paraspinal muscles close to the vertebrae and skull (see Figs. 1, 2) (Haavik and Murphy 2012; Haavik-Taylor and Murphy 2007a; Henderson 2012; Alcantara et al. 2013), and by doing so, activate or improve the function of these dysfunctional deep paraspinal muscles. This is, in turn, thought to affect how the CNS processes and integrates all subsequent sensory input. Hence, the brain more accurately perceives what is happening in and around the body, improving brain–body awareness, adaptability, coordination and motor control (see Figs. 1, 2) (Alcantara et al. 2013; Haavik-Taylor and Murphy 2007b; Haavik and Murphy 2012; Henderson 2012). The last few decades of basic science research suggests that spinal adjustments appear to improve the accuracy of the inner body and external world brain schemas, which improve limb, jaw and trunk motor control (see Figs. 1, 2) (Andrew et al. 2018; Baarbe et al. 2016, 2018; Daligadu et al. 2013; Farid et al. 2018; Haavik and Murphy 2011; Haavik et al. 2018a, b). For example, adjustments of neck CSMC problems have been shown to improve the accuracy of elbow joint position sense (Haavik and Murphy 2011), and 12 weeks of adjustments of CSMC problems have been shown to improve ankle joint position sense as well as improved accuracy of multisensory integration of visual and auditory inputs (Holt 2014; Holt et al. 2016). Improved proprioceptive awareness in the elbow and ankle after spinal adjustments suggests improved accuracy of inner body–brain schemas, and improved multisensory integration of visual and auditory sensory inputs suggests improved accuracy of external world brain schemas. Studies showing changes in jaw function (Haavik et al. 2018a, b) and female pelvic floor muscle function (Haavik et al. 2016a, b), and trunk muscle activation (Marshall and Murphy 2006) following spinal adjustments are also discussed below.

Multiple authors have suggested that vertebral column afferent input is responsible for poor motor control of the vertebrae, poor proprioception of the vertebral column, the development and recurrence of vertebral column pain, postural instability, as well as other symptoms, such as dizziness, visual disturbances and unsteadiness (Meier et al. 2018; Paulus and Brumagne 2008; Tong et al. 2017; Treleaven 2008, 2017). According to the literature, physical injury, pain, inflammation and acute or chronic physiological stress all appears capable of altering vertebral column proprioception (in particular head on neck) and motor control, by altering signalling from the deep paraspinal muscles or the central processing of such input (Hellström et al. 2005; Passatore and Roatta 2006; Brown et al. 2011; Butler and Moseley 2003; Hodges et al. 2006, 2009, 2014, 2015; James et al. 2016; Le Pera et al. 2001; Thunberg et al. 2001). It has, for example, been suggested that whiplash injuries change afferent input from the cervical spine that alters cervical reflex connections to the visual and vestibular systems and results in subsequent secondary disturbances, such as dizziness and visual disturbances (Solarino et al. 2009). However, it is not only cervical reflex connections that have been purported to change, as altered afferent input from the deep paraspinal muscles also appears to change the way various parts of the CNS integrates this afferent information with past memories and/or the current movement goal and impacts various anticipatory feedforward and/or feedback postural control mechanisms. This may impact the fine-tuning of movements or even the efference copies and/or the actual movement commands sent to the various muscles (see Fig. 1) (Marshall and Murphy 2006; Hodges and Moseley 2003; Meier et al. 2018; MacDonald et al. 2006). In any case, under any of these conditions that alter vertebral column afferent input, the CNS may not accurately sense what is occurring at that part of the vertebral column and may instead have to rely on past memories to co-ordinate vertebral motor control. This may lead to less than ideal motor control of the vertebral column and result in vertebral segmental microtraumas and self-perpetuating central segmental motor control problems that may, over time, result in recurrent spinal ache, pain or tension and the development of chronic vertebral column pain syndromes. Thus, any of these conditions, including physical injury, psychological stress, pain or inflammation, is thought to be able to initiate a central segmental motor control problem.

In summary, the mechanisms by which CSMC problems and spinal adjustments affect neuromuscular function has been explained over the past several decades by several models that converge towards the involvement of the CNS (“Practice Guidelines for Straight Chiropractic” 1992; Association of Chiropractic Colleges 1996; Christopher Kent 1996; Gatterman and Hansen 1994; Hart 2016; Lantz 1989; Leach 2004; Nelson 1997; Palmer 1910; Rosner 2016; Stephensen 1927). There is emerging evidence that altered vertebral sensory input from mechanically and/or chemically sensitive neurons in the paraspinal tissues (Bolton 2000; Haavik and Murphy 2012; Kent 1996; Pickar 2002) can modify central neural processing and integration of sensorimotor, multimodal, nociceptive and autonomic afferent information. These alterations are capable of changing sensorimotor, autonomic and visceromotor outputs (Alcantara et al. 2013; Bolton 2000; Haavik and Murphy 2012; Henderson 2012; Kent 1996; Pickar 2002; Taylor et al. 2010), likely by impacting the brains body schemas (see Figs. 1, 2) (Taylor et al. 2010; Holt et al. 2016a, b). There is also emerging evidence that improving paraspinal muscle function with spinal adjustments can rapidly alter central neural function in a variety of ways (see Figs. 1, 2) (Alcantara et al. 2013; Clark et al. 2011; Gyer et al. 2019; Haavik and Murphy 2012; Haavik-Taylor and Murphy 2007a; Henderson 2012; Hennenhoefer and Schmidt 2019; Kent 1996; Pickar 2002; Wirth et al. 2019) and that these changes outlast the altered changes of input, i.e. that they are neural plastic changes. It is unknown exactly how long the various neuroplastic changes last. Some changes are transient and only last between 20 and 30 min, such as N30 somatosensory-evoked potential (SEP) peak amplitude changes (Haavik-Taylor and Murphy 2007a), while others last at least 30 min, such as N20 SEP peak amplitude changes (Haavik-Taylor and Murphy 2007a). The N20 SEP peak changes in the study mentioned did not show any indication of ‘returning to baseline values’ as the N30 SEP peak amplitude changes did. Other studies, for example, Haavik et al. (2018a, b), have shown that muscle function changes following spinal adjustments, such as maximal bite force, may still be present a week after the adjustments were delivered. Thus, it appears that some of the neuroplastic changes that do occur following spinal adjustments appear to be transient, while others appear to last at least one week. Exactly how long the various central neural plastic changes last after adjustments needs to be further investigated in future studies. The second invited review will discuss in detail the central neural changes known to occur alongside vertebral column dysfunction as well as that which occurs after spinal adjustments or manipulations. The current review will now explore in more detail what direct evidence we have that vertebral column dysfunction, spinal adjustments or manipulations, can alter motor control, what sensory organs in the paravertebral tissues change following such mechanical perturbations and whether such changes in neuromuscular function occur due to changes at the spinal or supraspinal level of the CNS.

### Evidence for CSMC problems, spinal adjustments and spinal manipulation altering neuromuscular function, and whether this is due to spinal or supraspinal neuronal excitability changes

The main motor cortical and spinal output neuromuscular components that may be influenced by vertebral column dysfunction or HVLA adjustments and/or manipulations are the upper motor neuron (UMN), the lower motor neuron and its corresponding extrafusal muscle fibres, i.e. the motor units. The excitability of the UMN and single motor units (SMUs) can be influenced by many factors. The UMN is, for example, widely influenced by multiple pre-UMN networks that can have both an inhibitory and excitatory influence on the output of the UMN. To selectively assess the influence of vertebral dysfunction on the UMN or the SMUs themselves is not an easy task in humans and not yet possible in a non-invasive fashion. However, the entire corticomotor system can be assessed with several techniques. With carefully controlled experiments, it is possible to make educated conclusions about whether the function of the upper or the lower motor neuron has changed, or whether any changes are presynaptic to the corticospinal tract itself (AKA the pyramidal tract), or whether the changes in output are due to changes in the muscle contractile apparatus itself. Methods that have been used to assess whether early vertebral column dysfunction or the effects of spinal adjustments alter UMN or SMU outputs include the use of transcranial magnetic stimulation (TMS) (Haavik-Taylor and Murphy 2007b; Haavik et al. 2017; Haavik et al. 2016a, b; Haavik Taylor and Murphy 2008), the Hoffman reflex (H-reflex) (Christiansen et al. 2018; Holt et al. 2016a, b; Niazi et al. 2015), F waves (Haavik Taylor and Murphy 2008; Haavik-Taylor and Murphy 2007b), movement-related cortical potentials (MRCPs) (Haavik et al. 2017), V waves (Christiansen et al. 2018; Holt et al. 2016a, b; Niazi et al. 2015), surface electromyography (EMG) (both single electrodes and high density (HD) electrodes) (Haavik-Taylor and Murphy 2007b; Haavik Taylor and Murphy 2008; Haavik et al. 2017, 2018a, b), intramuscular EMG (Haavik et al. 2018a, b), fibre type analysis and force measures (Christiansen et al. 2018; Haavik et al. 2018a, b; Niazi et al. 2015; Holt et al. 2019). This section will discuss these studies and summarise the current state of the literature on this topic. It will focus on how the output of UMN and SMU can be assessed and will discuss the literature that has explored the effects that vertebral column function, dysfunction, spinal adjustments, and spinal manipulation has on their output.

### Direct strength or background muscle tone changes following spinal adjustment or manipulation

The ability of spinal adjustments or spinal manipulation to alter corticomotor excitability is supported by multiple studies that have shown changes in force output or background muscle activity following single or repeated sessions of spinal adjustments or manipulations (see Table 1) (Christiansen et al. 2018; Haavik et al. 2016a, b, 2018a, b; Niazi et al. 2015, 2020; Dunning and Rushton 2009; Cleland et al. 2004; Holt et al. 2019; Botelho and Andrade 2012; Hillermann et al. 2006; Vining et al. 2020; Keller and Colloca 2000; Humphries et al. 2013; Grindstaff et al. 2009; Fernandez-Carnero et al. 2008; Galindez-Ibarbengoetxea et al. 2017; Lo et al. 2019). As mentioned earlier (and highlighted in Table 1) the various authors of these publications may or may not have used the terminology ‘central segmental motor control (CSMC) problems’ to describe any dysfunctional spinal segments. They may or may not have used the term ‘spinal adjustment’ if HVLA thrusts were delivered to an area of spinal dysfunction. Therefore, to clarify whether or not the HVLA thrust was delivered to a CSMC problem, each publication discussed below has been classified as delivering spinal adjustments if the HVLA thrusts were directed at a dysfunctional segment. In contrast, other publications have been classified as delivering spinal manipulations if they describe HVLA thrusts that were directed at a segment of the spine for another reason or if it was not specified why they chose to deliver an HVLA thrust at all (see Table 1 for this). The exact wording of the original authors regarding their reasoning for choice of the segment that an HVLA thrust was directed at is identified in Table 1 (Column 3), and the page number of the original publication where this description is found in the original publication is noted in Column 3 along with the reference of that publication in Column 1. Examples of other reasons for delivering HVLA thrusts (as identified in Table 1**)** could include that the participants had pain at that level of the spine, or the segment was chosen at random or that they could have a segmental effect on the nerve roots or associated motor neuron pools. Thus, they may have chosen a lower cervical segment to manipulate, regardless of whether this segment displayed any clinical signs of joint dysfunction, simply because the nerve roots at that level innervate the upper limb. As discussed in this review, it is highly unlikely that applying HVLA thrusts at the spine alters transmission of information flow via the nerve roots at the level of manipulation, unless that individual displays radicular symptoms at that level (which is not the case in these publications, as outlined in Table 1). Other reasons could be because the authors believed spinal manipulation should be directed at a region of the spine that their subjects felt pain, in line with structural pathology models of pain. In this case, their reasoning behind applying HVLA thrusts to a spinal segment was that the manipulations in the regions that the subjects felt pain would be altering nociceptive inputs responsible for generating the pain feelings or have a direct impact on pain generating structures in that part of the spine. As discussed in this review, this is an outdated model of pain, particularly chronic pain, as pain is now known to be generated by the brain in response to tissue damage, or even just the potential for tissue damage. Finally, other authors give no justification for the site of manipulation at all, and they appear to have pre-determined a spinal level to direct their HVLA thrust for unknown reasons. As it is highly likely that thrusting at a CSMC problem will have a different neurophysiological impact and thus can influence its ability to change neuromuscular function, compared with thrusting at a relatively healthy spinal segment (i.e. a segment that does not display any clinical indicators of a CSMC problem), we have for the purposes of this review highlighted this difference by classifying studies into either ‘adjustment’ studies if they directed their thrust at a CSMC problem, or ‘manipulation’ studies if they directed their thrust at segments that do not display any clinical indicators of being a CSMC problem, or if the reason for choosing a specific segment to thrust on was not specified. The following discussion has therefore used this classification. Table 1 contains the original authors' justification for applying an HVLA thrust, or whether this detail was not provided at all.

**Table 1:**
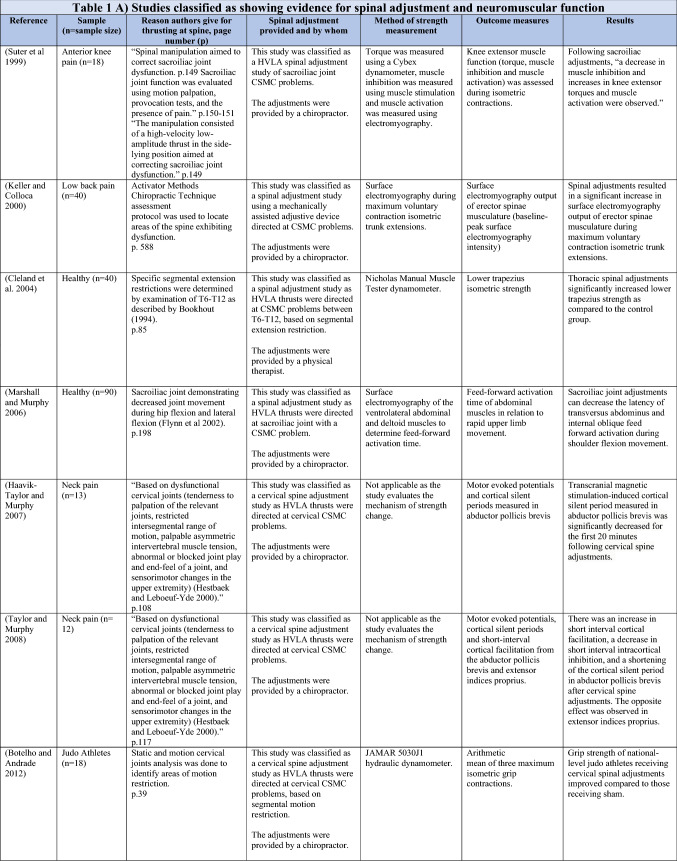

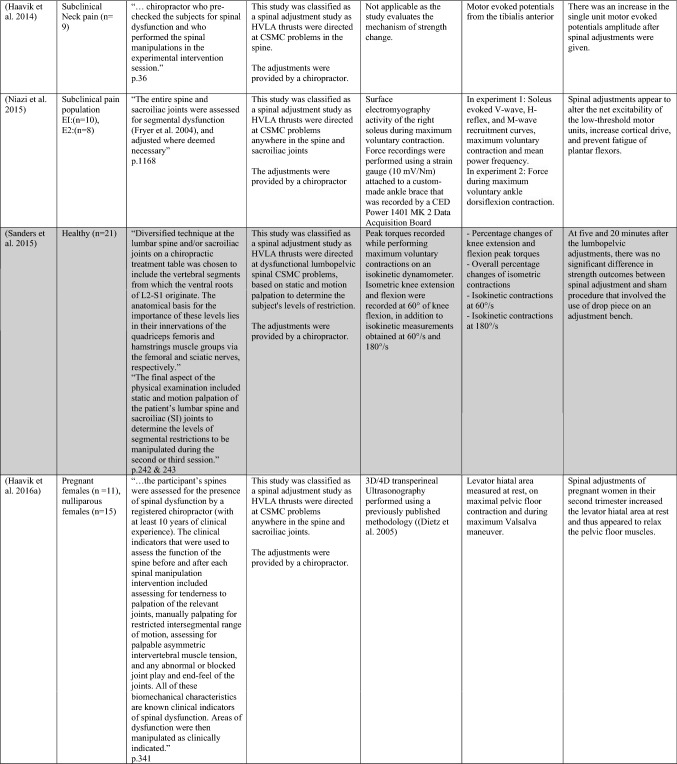

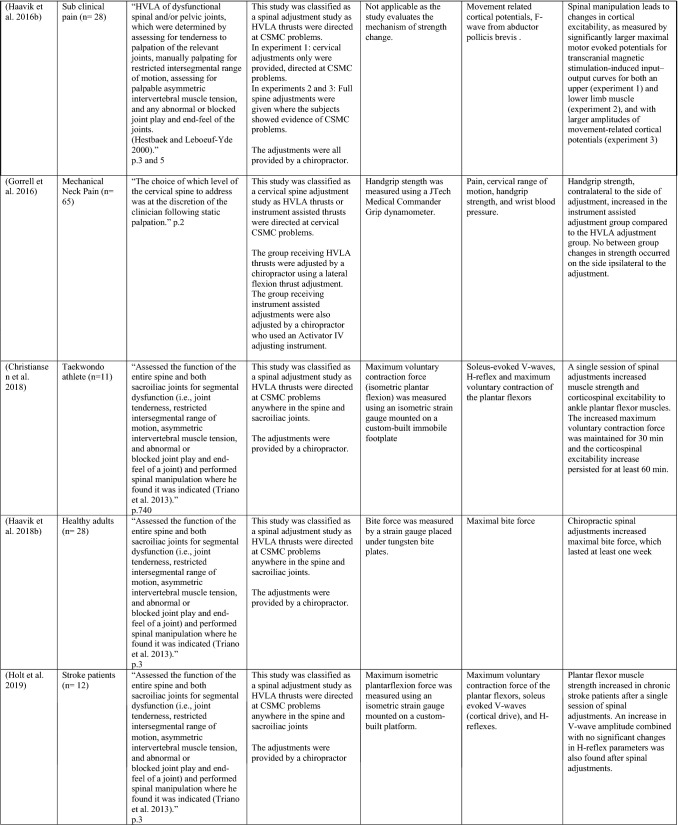

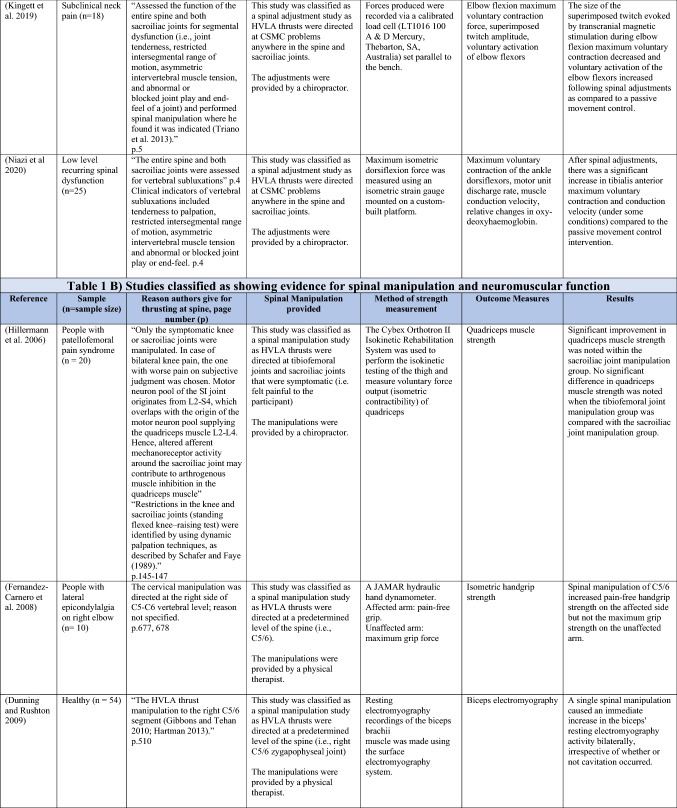

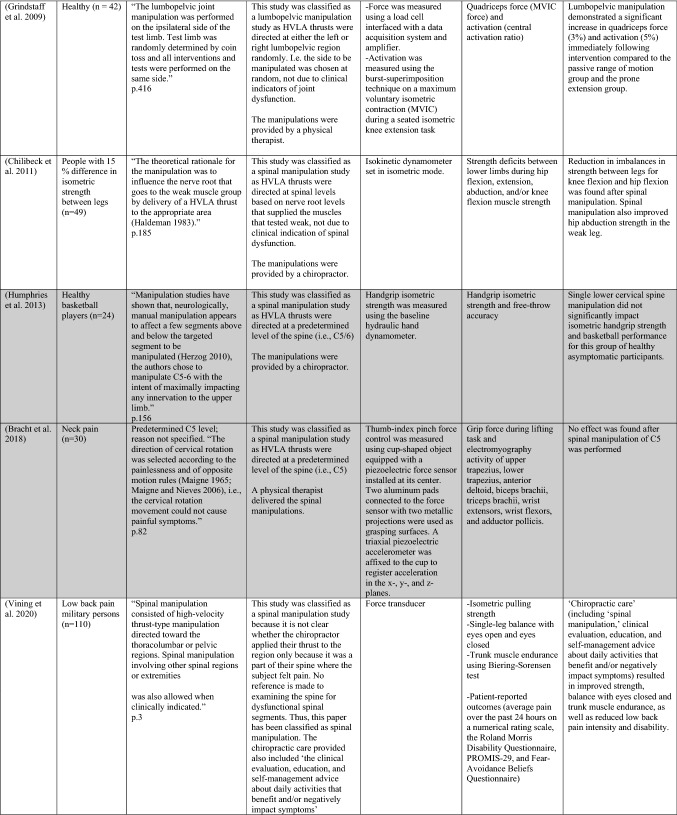
Studies showing evidence for either spinal adjustments or spinal manipulation altering neuromuscular function [The studies have been categorised (column 4) as delivering spinal adjustments (**A**) or spinal manipulation (**B**) based on the reason authors give for thrusting on the spine, with their exact wording presented in column 3]

Studies that found no change in outcome measures have been highlighted in grey. *EMG* electromyography, *HVLA* high-velocity low-amplitude thrust, *CSMC problems* central segmental motor control problems

When considering studies that have investigated upper limb muscle function, one study involving healthy asymptomatic individuals (Dunning and Rushton 2009) reported a significant increase in resting EMG activity of bilateral biceps brachii muscles following spinal manipulation of right lower cervical segments (C5/6) as compared to sham spinal manipulation (the spinal manipulation setup, but without the delivery of the HVLA thrust at the C5/6 segment) or no manual contact. In another study, using the interpolated twitch technique with TMS pre- and post-spinal adjustments, central cortical inhibition to the elbow flexor muscles was significantly reduced in 18 people with subclinical spinal pain (SCSP) (Kingett et al. 2019). SCSP refers to recurring, intermittent, mild spinal pain, ache, or tension for which treatment has not yet been sought. This analytical method found that voluntary activation of the elbow flexors increased immediately after one session of spinal adjustments (Kingett et al. 2019). The decrease in the amplitude of superimposed twitch during elbow flexion maximum voluntary contractions (MVC) following the spinal adjustments suggests facilitation of cortical motor output to the elbow flexors (Kingett et al. 2019). Another study found a significantly greater increase in lower trapezius muscle strength after a single session of spinal adjustments of thoracic spine CSMC problems as compared to a placebo intervention in asymptomatic individuals (Cleland et al. 2004). The application of a single session of spinal adjustments of CSMC problems has also been shown to alter handgrip strength measured using a hydraulic hand dynamometer (Botelho and Andrade 2012). This significant increase in handgrip strength was found in a group of judo athletes (Botelho and Andrade 2012). In people with mechanical neck pain, hand grip strength on the contralateral side to adjustment was noted after an instrument-assisted adjustment combined with stretching (Gorrell et al. 2016).

Interestingly, in people with lateral epicondylalgia, a single session of spinal manipulation of C5/6 increased the pain-free handgrip strength of the affected arm, while there was no change in the maximal grip strength of the unaffected arm (Fernandez-Carnero et al. 2008). However, in another study, a minimal change in isometric handgrip strength that did not reach statistical significance was noted in asymptomatic male recreational basketball players following a single session of spinal manipulation of the C5/6 spinal level (Humphries et al. 2013). Similarly, no changes were reported in grip force during a lifting task when the C5 level was manipulated (Bracht et al. 2018). It is possible that the handgrip strength in the basketball players did not reach significance because they were all manipulated at a pre-determined level (C5/6) regardless of whether this was clinically warranted or not. Some of them may well have had a CSMC problem at that level and this may have been why there was a slight increase in strength in these basketball players, but since others may not have had any dysfunction at this segment, an HVLA thrust at that level may not have altered their handgrip strength, meaning the slight average increase in strength was not significant (Humphries et al. 2013). This needs to be explored further in future studies.

Several studies have also shown increases in lower limb muscle strength, such as the plantar flexor muscles, after a single session of spinal adjustments of CSMC problems (Holt et al. 2019; Christiansen et al. 2018; Niazi et al. 2015). One of these studies reported a 16% increase in ankle plantar flexor strength after spinal adjustments in a group with SCSP (Niazi et al. 2015). Another study in elite taekwondo athletes reported a 7.6% increase in plantar flexor muscle strength following a single session of spinal adjustments (Christiansen et al. 2018). To explore the opposite end of the health spectrum, the same research group used the same research design in a chronic stroke patient population who had lost their ability to cortically activate their muscles and had ongoing plantar flexor muscle weakness (Holt et al. 2019). Despite that, these chronic stroke patients, with ongoing lower limb muscle weakness, showed a significant increase in plantarflexion muscle strength of 64.2% on average following a single session of spinal adjustments (Holt et al. 2019). The greater percentage increase in strength in this stroke study compared to previous studies may be due to the stroke patients having weaker muscles to begin with, so they had more opportunity to increase in strength. Other groups have also shown increases in strength in lower limb muscles following spinal manipulations. For example, a single session of spinal manipulation was shown to increase quadriceps strength in healthy individuals (Grindstaff et al. 2009) and people with patellofemoral pain syndrome (Hillermann et al. 2006). These results were similar to a previous study which showed an increase in quadriceps strength following adjustments of sacroiliac joints with a CSMC problem in participants with anterior knee pain (Suter et al. 1999).

A recent study using both HD sEMG and intramuscular EMG explored how muscle strength increases occur following spinal adjustments of CSMC problems (Niazi et al. 2020). They found that spinal adjustments of CSMC problems again resulted in significant increases in strength in the tibialis anterior (TA) muscle, and they found a significant increase in TA muscle motor unit action potential conduction velocity without changes in motor unit discharge rate in people with SCSP (Niazi et al. 2020). This suggested that the spinal adjustment-induced increase in strength was, in part, due to increased recruitment of larger, higher threshold motor units. However, it is difficult to be confident of this without measuring the recruitment threshold of many units. This finding could also be due to a reduction in antagonistic muscle activity (Niazi et al. 2020). Yet, not all studies have shown significant increases in strength following spinal adjustments (Sanders et al. 2015). Sanders and colleagues investigated the effect of manual spinal adjustments of lumbar spine and/or sacroiliac joint CSMC problems vs a sham drop table intervention on concentric knee extension and flexion forces in 21 asymptomatic, college-aged subjects (Sanders et al. 2015). There were no significant differences between the effects of lumbosacral adjustments or the sham intervention in the percentage changes of knee extension and flexion peak torques at 5 and 20 min post-intervention (Sanders et al. 2015). There are several reasons why this may be the case. The spinal adjustments in this study targeted lumbosacral CSMC problems only, due to the potential for aberrant afferent input from the lumbosacral CSMC problems or the adjustments at these levels impacting the relevant segmentally innervated lower limb musculature. Now that we know that spinal adjustments are more likely to alter central multimodal integration, it may be that there were other parts of the spine that actually needed to be adjusted, such as the upper cervical spine, to induce significant strength changes. The adjustments delivered in several of the latest studies showing significant increases in strength were to any CSMC problem anywhere in the spine, i.e. the chiropractors checked and adjusted the entire spine for CSMC problems (Niazi et al. 2020; Holt et al. 2019; Christiansen et al. 2018). It is also possible there was a type II error that occurred or that the sham in this study was not a true sham, as the overall strength changes showed a trend towards an increase post the adjustment vs. the sham (overall percentage changes of isometric contractions: spinal adjustment 4.0% ± 9.5% vs. sham 1.2% ± 6.3%, *p* = 0.067). The sham involved the drop of a table piece that would likely impact the paraspinal tissues, particularly paraspinal muscle spindles, due to the drop itself, despite there being no direct force application over the CSMC problem (Sanders et al. 2015). It is also possible that the effects of spinal manipulation depend on the state of the muscle prior to the HVLA thrust. Reductions in imbalances in strength between the legs for knee flexion, hip flexion and hip abduction have been reported following spinal manipulation that displayed at least a 15% difference in isometric strength between legs prior to the manipulation intervention (Chilibeck et al. 2011). These factors should be investigated in future studies, with more careful measurement and assessment of the subjects both prior to and after the spinal adjustment or manipulation interventions.

The effects of spinal adjustments on trunk neuromuscular function have also been explored. A preliminary clinical trial in people with low back pain found a significant increase in erector spinae isometric MVC muscle output measured via surface EMG following a single session of spinal adjustments of CSMC problems using the Activator Methods Chiropractic Technique (AMCT) assessment protocol or a sham treatment session or a control session with no intervention (Keller and Colloca 2000). These subjects were adjusted with the HVLA thrust delivered using an Activator II Adjusting Instrument (AAI II; Activator Methods International, Ltd, Phoenix, AZ) and the increase in surface EMG was recorded over the erector spinae musculature at L3 and L5 during an isometric trunk extension contraction, which was taken as an indication of improvement in paraspinal muscle strength (Keller and Colloca 2000). MVC strength and surface EMG activity are not equivalent; thus the reader needs to be cautious with their interpretation. EMG over a single muscle is not a method for determining the MVC of a particular joint. The MVC force comprises several agonistic and antagonistic muscles, i.e. measures net force produced by multiple muscles. However, this study does indicate a change in neuromuscular function of the erector spinae muscles following the adjustment session (Keller and Colloca 2000). Interestingly, this study has been followed up with a recent RCT in active-duty military personnel with low back pain that found improved isometric pulling strength from a semi-squat position following 4 weeks of chiropractic care that included thoracolumbar and/or pelvic manipulation, education and self-management advice about daily activities that may benefit as compared to a wait-list control group (Vining et al. 2020). This study did not specify whether or not the chiropractor applied their HVLA thrusts at dysfunctional spinal segments or not, but simply noted they provided manipulations at the lower back or other spinal regions or extremities as ‘clinically indicated’. However, they did not clarify what ‘clinically indicated’ meant. Thus, it is possible these HVLA thrusts were directed at CSMC problems. However, it is also possible they applied manipulations to regions of the spine or extremities where the subjects complained of pain. Isometric pulling strength, in this study, was measured by asking the participants to maintain a semi-squat position and gradually pull a bimanual handle attached to a force transducer until a maximum was reached. The mean maximum pulling force measured after 4 weeks of chiropractic care increased by 5.08 kg, whereas it decreased by 7.43 kg in the wait-list group. This study supports the notion that chiropractic care, which includes spinal manipulation, can increase trunk muscle strength in active-duty military personnel with low back pain. This study also highlights the need to clearly operationally define terms in such studies, as it is currently unclear from this study whether or not the choice of the segment to thrust at was based on the presence of spinal dysfunction or simply the presence of pain.

Another adjustment study has shown that adjusting sacroiliac CSMC problems can improve feedforward activation (FFA) times of deep abdominal muscles in relation to rapid upper limb movements in young, healthy males (Marshall and Murphy 2006). Those who met the criteria for delayed FFA (failure of deep abdominal activation within 50 ms of deltoid activation, which affected 17 of the 90 subjects in this study, i.e. almost 19%) were also reassessed 6 months later (Marshall and Murphy 2006). Thirteen of the original 17 were available to be remeasured at a 6-month follow-up and the latency of delayed FFA was found to be highly consistent with their baseline measures. These subjects then underwent sacroiliac adjustments on the side, which was found to have the greatest decrease in joint movement in all subjects. There was a significant improvement, by on average 38.4%, in FFA times for this group when remeasured immediately after the sacroiliac adjustments (Marshall and Murphy 2006). This suggests that such protective postural reflexes when absent do not ‘come right’ on their own over a 6-month period in healthy young males yet shows immediate improvements after a single adjustment of a sacroiliac CSMC problem. It is important to now explore how long such improved protective postural reflexes last following adjustments and whether this has any clinical impact preventing pain development. For example, Cholewicki et al. (2005) showed in a prospective observational study following 303 college students for 2–3 years that delayed trunk muscles reflexive responses significantly increased the odds of sustaining a low back injury during the study period. Furthermore, it is well documented in the literature that people with recurrent and/or chronic spinal pain have delayed or altered trunk muscle recruitment patterns, including poor postural feedforward protective reflexes (Silfies et al. 2009; Hodges 2001; Hodges and Richardson 1996, 1999; MacDonald et al. 2009; Radebold et al. 2000; Marshall and Murphy 2008). Therefore, as spinal adjustments appear to be capable of improving feedforward protective postural reflexes (Marshall and Murphy 2006), future studies should explore how long adjustment-induced changes last, and future clinical trials could explore whether a period of chiropractic care could improve protective postural reflexes as well preventing or reducing the odds of sustaining a low back injury and/or developing recurrent and/or chronic spinal pain.

Studies showing increases in muscle strength following spinal adjustments or manipulation have not been limited to the limbs or trunk muscles. A single session of spinal adjustments also increased pregnant women’s pelvic floor levator ani-hiatal area at rest, suggesting the spinal adjustments had altered the background activity of these muscles (Haavik et al. 2016a, b). The relaxation of the pelvic floor muscles was found in pregnant women in their second trimester. It did not occur in the nonpregnant control participants, suggesting that this effect may be pregnancy-related. In another study in people with SCSP, a single session of spinal adjustments significantly increased jaw strength as compared to sham spinal adjustments (Haavik et al. 2018a, b). The increase was maintained at one-week follow-up. Interestingly, the muscles involved with jaw clenching to produce maximum bite force, such as the masseter muscle, the temporalis, medial pterygoid, and lateral pterygoid, are all innervated by the anterior division of the mandibular division of the trigeminal nerve. This strongly suggests that the impact of the adjustments of CSMC problems must have a central neural impact, as they are changing the function of cranial nerve innervated muscles.

In summary, multiple previous studies have documented direct evidence for changes in neuromuscular function, including direct strength increases following spinal adjustments of CSMC problems in a variety of muscles and a variety of populations (see Table 1) (Christiansen et al. 2018; Haavik et al. 2018a, b; Holt et al. 2019; Keller and Colloca 2000; Niazi et al. 2015; Suter et al. 1999), with mixed results following spinal manipulation at a pre-determined cervical spinal level (Humphries et al. 2013; Dunning and Rushton 2009; Bracht et al. 2018). This suggests that manipulation of the vertebral column that is not based on the presence of clinical indicators of CSMC problems can at times be able to induce central neural plastic changes. However, it may have less of a central neural effect compared to adjustments of CSMC problems. It is also possible that some of the publications that did not specify how they chose to direct their HVLA thrusts did direct them at CSMC problems, and this may be the reason for the induced central neural plastic changes (e.g., Vining et al. (2020)). The significant increases in force that occur after adjustments of CSMC problems have been shown in various muscle groups, such as upper limb muscles (Cleland et al. 2004; Kingett et al. 2019), lower limb muscles (Christiansen et al. 2018; Holt et al. 2019; Niazi et al. 2015, 2020), trunk muscles (Keller and Colloca 2000), and jaw clenching muscles (Haavik et al. 2018a, b). Even the resting state of pelvic floor muscles of primigravid women in their second trimester has been shown to change after spinal adjustments (Haavik et al. 2016a, b). Therefore, these studies provide evidence for the ability of spinal adjustments and, to a lesser degree, spinal manipulation to directly change muscle strength and background tone. Future studies need to explore how long these changes in muscle strength last following adjustments and what clinical relevance they have.

### The mechanisms of strength changes following spinal adjustments or manipulation

To better understand the exact neuromuscular changes that occur following spinal adjustments or manipulation, multiple different neurophysiological techniques can be utilised, including the measurement of reflex responses, such as the H-reflex and V-wave. The H-reflex measures presynaptic inhibition and motoneuron excitability (Nordlund Ekblom 2010) and the V-wave measures changes in supraspinal input to the motor neuron pool (Vila-Chã et al. 2012). So far, three studies (Holt et al. 2019; Christiansen et al. 2018; Niazi et al. 2015) have evaluated the effect of spinal adjustments on H-reflex and V-wave responses based on current best practice for recording (Tucker et al. 2005) and analysing (Brinkworth et al. 2007) these measures.

In people with SCSP, a single session of spinal adjustments significantly reduced the threshold for eliciting the H-reflex, increased the V-wave amplitude and increased plantar flexor force by 16% (Niazi et al. 2015). This was accompanied by a lack of fatigue associated with repeated, maximal muscle contractions done while recording V-waves (Niazi et al. 2015). In comparison, participants in the control group became weaker and showed signs of fatiguing (Niazi et al. 2015). This indicates that spinal adjustments affect the H-reflex pathway, increase the cortical drive to muscles, prevent fatigue from developing during repeated maximum voluntary contractions and enable the CNS to produce greater muscle force (Niazi et al. 2015). Notably, the increase in strength was likely due to supraspinal changes, as there were significant V-wave changes, which reflects cortical drive to muscles. In contrast, the H-reflex changes that reflect changes at the level of the spinal cord were minimal (Niazi et al. 2015). Interestingly, similar supraspinal neuroplastic changes have previously been observed in a study investigating the effects of 3 weeks of strength training (Vila-Chã et al. 2012). In sedentary healthy individuals, 3 weeks of strength training significantly increased the V-wave amplitude (as measured by V/Mmax ratio) by just over 55%, increased the MVC of the right soleus (measured by sEMG) by 14.4%, and significantly decreased the H-reflex threshold by 4.7%. In comparison, Niazi et al. (2015) found that application of a single session of chiropractic adjustments in males with a history of subclinical spinal pain significantly increased V-wave amplitude (V/Mmax ratio) by 45%, increased the MVC of the right soleus by almost 60% (sEMG) and 16% (absolute force) and significantly decreased the H-reflex threshold by 8.5%. This indicates that the neuroplastic impact of a single session of adjusting CSMC problems was equivalent to what occurs in the brain following 3 weeks of strength training and suggests spinal adjustments may have a similar mechanism to that of strength training. This should be explored further in future research studies.

H-reflexes and V-waves have also been measured in a group of elite taekwondo athletes (Christiansen et al. 2018) and chronic stroke survivors (Holt et al. 2019). In both these populations, a single session of spinal adjustments caused significant changes in V-wave amplitude without any change in the H-reflex. This was accompanied by increased average plantar flexor strength of 7.6% and 64.2% in elite taekwondo athletes (Christiansen et al. 2018) and chronic stroke survivors (Holt et al. 2019), respectively. These findings further support the ability of spinal adjustments to change cortical drive (Christiansen et al. 2018; Holt et al. 2019). It would be interesting to see what effect spinal manipulation, that is not based on the presence of clinical indicators, has on the H-reflex and V waves and should be followed up in future studies. It is also critical to ascertain how long these immediate changes in strength last, and whether or not these strength changes impact the individuals clinically, or professionally in sports populations, as well as whether such strength changes occur for other muscles and for other populations.

**Fig. 3:**
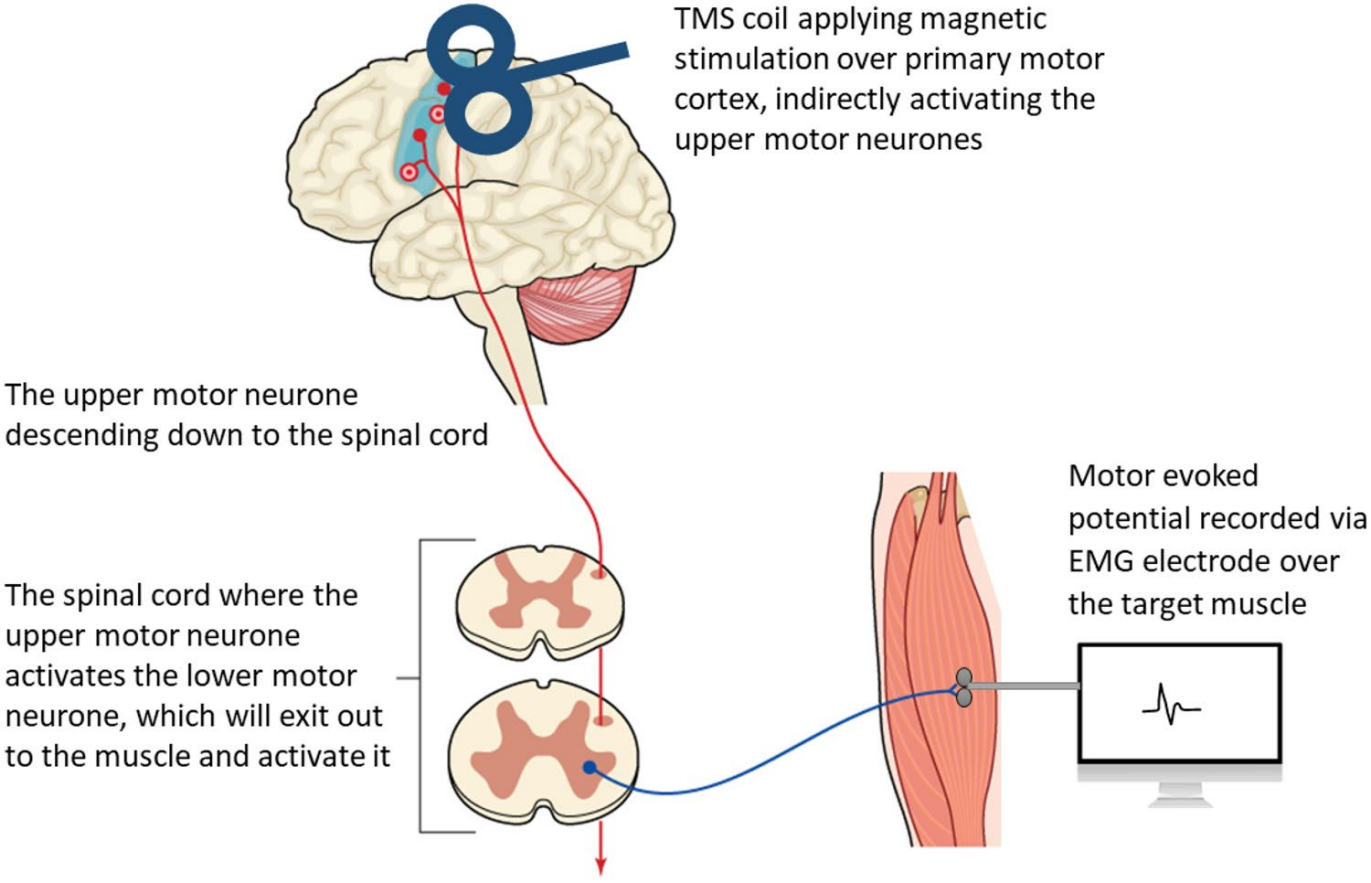
A diagram illustrating how transcranial magnetic stimulation (TMS) over the primary motor cortex (M1) indirectly activates the upper and lower motor neurons of the corticospinal pathway to cause a muscle contraction which can be recorded as a motor-evoked potential (MEP) using EMG electrodes

Another method that can help investigate the mechanisms of strength changes that occur following spinal adjustments and manipulation is transcranial magnetic stimulation (TMS). The effect of spinal adjustments on corticomotor excitability has been evaluated by recording TMS-induced motor-evoked potentials (MEPs), cortical silent periods (CSPs), short-interval intracortical inhibition (SICI), short-interval intracortical facilitation (SICF) and stimulus–response curve (SR curves, also known as recruitment curve or input–output curves) pre- and post-adjustments of CSMC problems (Haavik et al. 2017, 2018a, b; Haavik-Taylor and Murphy 2007b; Haavik Taylor and Murphy 2008). To our knowledge, no study has yet explored the effects of spinal manipulation of spinal segments (i.e. HVLA thrusts that is not clinically warranted) using TMS. TMS is a non-invasive technique (Haavik et al. 2018a, b; Haavik-Taylor and Murphy 2007b; Haavik Taylor and Murphy 2008; Barker et al. 1985; Merton and Morton 1980; Haavik et al. 2017) that delivers a rapidly changing magnetic field to produce electrical currents in brain tissues (Barker et al. 1991; Cadwell 1990) and thus, activates the human cortex (Geddes and Bourland 1983). Studies have shown that TMS does activate the same neurons that are activated during voluntary movements (Bawa and Lemon 1993). The activation of these muscles can be recorded and measured with EMG over the target muscle (Bestmann et al. 2008; Julkunen et al. 2009). The potentials evoked and measured over the target muscle are called MEPs (Rothwell 1997) (refer to Fig. 3). The size of the MEPs is thought to reflect the net excitability of both excitatory and inhibitory pre-upper motor neuron networks and their ability to activate the corticospinal tract originating in M1 projecting to the target muscle (Muellbacher et al. 2000; Rothwell 1997). When the magnetic stimulus is delivered during active contraction of the tested muscle, the MEP is followed by a silent period (Inghilleri et al. 1993; Rossini 1990; Wilson et al. 1993), where there is minimal muscle activity. This is known as the TMS-induced CSP. Any change in the size of the MEP or length of the CSP reflects a change in motor control (Fritz et al. 1997; Kukowski and Haug 1992).

Two repeated measures studies evaluated the effect of spinal manipulation on MEPs and CSPs in 13 and 12 individuals with SCSP (Haavik-Taylor and Murphy 2007b; Haavik Taylor and Murphy 2008). Both studies recorded MEPs and CSPs from the abductor pollicis brevis (APB) muscle of the thumb, pre and post a cervical spine adjustment session and on another day, pre and post a passive head movement control session, with the order of the two interventions randomised (Haavik-Taylor and Murphy 2007b; Haavik Taylor and Murphy 2008). Both these studies showed a consistent and significant shortening of the CSP following spinal manipulation only, with no changes in the MEP amplitude (Haavik-Taylor and Murphy 2007b; Haavik Taylor and Murphy 2008). As is the case in many studies involving a manual intervention, it is not possible to rule out a placebo effect because the participants were not blinded to which intervention was applied. However, this study indicated that adjusting CSMC problems appears to alter the way the corticomotor system controls the thenar muscles of the thumb. This may relate to changes occurring in the way the cortex processes proprioceptive information from the thenar area of the thumb, as measured with alterations in cortical SEP peak amplitudes (Haavik-Taylor and Murphy 2007b; Haavik Taylor and Murphy 2010a, b; Lelic et al. 2016) and most likely involves the prefrontal cortex (Lelic et al. 2016). This will be discussed in greater detail in the second invited review.

Although these CSP changes were initially assumed to be inhibitory motor control phenomena based on the literature at the time (Cantello et al. 1992; Chen and Garg 2000; Inghilleri et al. 1993; Kukowski and Haug 1992), work by Türker and colleagues (Turker and Cheng 1994), constructing peristimulus frequencygrams (PSF) from single motor unit recordings, demonstrated that evoked potentials previously thought to be inhibitory, were in fact excitatory and vice versa (Turker and Cheng 1994; Turker and Powers 2005). Therefore, a third study was conducted to investigate the changes in CSP in the TA muscle, using single motor unit data and a combination of surface EMG (sEMG), peristimulus time histograms (PSTH) and PSF analyses to explore whether the shortening of the CSP seen after spinal adjustments was in fact inhibitory in nature (Haavik et al. 2016a, b). This study confirmed that spinal adjustments induced a consistent shortening of the CSP and increased the amplitude of individual I-waves, i.e. TMS-evoked descending corticospinal activity originating from indirect or trans-synaptic activation of the pyramidal tract or corticospinal tract UMN’s. Thus, the shortening of the CSP found after spinal adjustments are in fact clearly excitatory events because the discharge rate underlying them was higher than the background SMU firing rate (Haavik et al. 2016a, b). Individual peaks were seen in the PSTH that were separated by a few milliseconds around the latency one would normally record an MEP (Haavik et al. 2016a, b). These individual peaks were clearly observed in all the single motor units tested, and as they were observed around the latency that the MEP is usually recorded, they were interpreted to reflect human I-waves, as previous scientists have also done (Awiszus and Feistner 1994).

Interestingly, the changes in I-wave amplitudes following the spinal manipulation intervention were shown to be genuine excitatory events as the discharge rates underlying these peaks were higher than the background firing rates (Haavik et al. 2016a, b). These studies, therefore, suggest that more low-threshold motor units are recruited after spinal adjustments, while no changes in the motor unit firing rates were observed. Using this method does not allow for the exploration of effects of higher-threshold motor units because at higher contraction levels it is not possible to identify single motor units, as they are superimposed on top of each other. Thus, to explore what happens in higher-threshold motor units, other techniques need to be applied, such as TMS-induced stimulus response curves.

Spinal adjustments have also been shown to impact other TMS-evoked paired-pulse measures, such as SICF and SICI. These types of paired-pulse TMS techniques have for the past few decades been utilised to non-invasively investigate the excitability of various inhibitory (Chen and Garg 2000; Ilic et al. 2002; Kujirai et al. 1993) and excitatory (Hanajima et al. 2016; Tokimura et al. 1996; Ziemann et al. 1998) neuronal networks at the motor cortical level. It was found that application of spinal adjustments in 12 participants with SCSP decreased SICI in the APB muscle and increased SICI in the extensor indicis proprius (EIP) muscle (Haavik Taylor and Murphy 2008). In contrast, SICF increased in the APB, and decreased in the EIP following the spinal adjustment intervention only (i.e. not following the control intervention) (Haavik Taylor and Murphy 2008). This indicates that spinal function or input to the CNS from the spine can impact the background corticomotor excitability to muscles of the upper limb in a muscle-specific manner.

The effects of spinal adjustments on corticomotor excitability can also be evaluated using the TMS-induced SR curve (Haavik et al. 2017). The SR curve reflects recruitment patterns of the lower motor neuron pool (Devanne et al. 1997). Several measures can be made from these recruitment curves, such as the threshold at which a MEP response occurs and the steepness of the slope of the SR curve (Devanne et al. 1997). The steeper the slope, the faster motor neurons are activated at each increasing stimulation level (Devanne et al. 1997). The top of the SR curve, or plateau level, reflects the maximum output you can get from TMS over that particular target muscle (Devanne et al. 1997). This plateau, also known as MEPmax, reflects the maximum net output of all excitatory or inhibitory inputs to the pyramidal tract neurons responsible for the TMS-induced SR curve (Devanne et al. 1997). The effect of spinal manipulation on recruitment patterns of lower motor neurons has been evaluated using the TMS-induced SR curves for an upper limb muscle (the APB), along with F waves, before and after either spinal adjustments or a control intervention for the same SCSP subjects on two different days (Haavik et al. 2017). On two additional days, lower limb TMS-induced SR curves and movement-related cortical potentials (MRCPs are also known as bereitschaftpotentials) were recorded from the TA pre and post-spinal adjustments (Haavik et al. 2017). Spinal adjustments resulted in a 54.5% ± 93.1% increase in the maximum MEP. The plateau of the SR curve (MEPmax) for both the upper and lower limb muscle increased significantly (by 54.5% ± 93.1% and 44.6% ± 69.6%, respectively), and there was a significant increase for all components of the MRCP [the early bereitschaftpotential (EBP), late bereitschaftpotential (LBP) and also the peak negativity (PN)]. The change in MRCP noted after the spinal adjustment intervention indicates a change in motor preparatory activity occurring primarily within the supplementary motor area of the brain (Haavik et al. 2017). The results of this study indicate that the changes in muscle force output following spinal adjustments is at least in part occurring at the cortical level, because it leads to significantly larger MEPmax for TMS-induced input–output curves for both an upper and lower limb muscle, as well as due to the larger amplitudes of MRCP components post-adjustment, while no changes were observed in the spinal measures (i.e. in this case F wave amplitudes or persistence).

In summary, these studies indicate that the changes in neuromuscular function that occur after spinal adjustments of CSMC problems impact the CNS and are primarily due to supraspinal excitability changes, and to a lesser degree, due to spinal cord excitability changes (see Table 1). The exact nature of such supraspinal changes will be explored in the second invited review. Much less is known about the effects of spinal manipulation of spinal segments that do not have CSMC problems (i.e. where there is no clinical evidence of spinal dysfunction) on the excitability of the UMN or SMU. This should be explored in future studies, as manipulation of freely moving, potentially better functioning vertebral segments may well have a different neurophysiological impact on the CNS that may be relevant to clinical practice. For example, it may be that manipulating segments that do not have CSMC problems results in fewer beneficial clinical outcomes for the patient because they may have a smaller or insignificant neurophysiological impact on the brain and neuromuscular motor control and function. Alternatively, it may not matter whether you manipulate the spine at levels with no clinical indictors of dysfunction or carefully determine dysfunctional segments, in which case this should then inform education and clinical practice. This current review will now focus on what sensory organs in the paraspinal tissues are affected by vertebral column dysfunction, spinal adjustments and spinal manipulation.

### Sensory receptors that could contribute to, or are known to be involved in, neuromuscular functional changes due to vertebral column dysfunction, spinal adjustments or spinal manipulation

It is well known in the literature that the CNS receives sensory receptor inputs originating both from inside and from outside the body and both can influence and alter motor control and neuromuscular function (Tagliabue and McIntyre 2014). The CNS utilises different sensory modalities to create the various maps that are used for various functions (Harris et al. 2015). For example, when the brain creates a map of sound localisation, the sound localisation map is influenced by somatosensory, visual, vestibular, and auditory information, as well as from proprioceptive and mechanoreceptive information and from efference copies from the brain itself, all of which can impact neuromuscular function (Harris et al. 2015; Bellan et al. 2017). This review covers the science behind how vertebral proprioceptive and mechanoreceptive information can be altered if there is spinal dysfunction present, and also discusses the evidence for spinal adjustments or manipulations being capable of altering such paraspinal mechanoreceptive input to the CNS. In the second invited review the literature showing that spinal adjustments or manipulations also appear to alter these maps will be discussed. For example, research has shown that people with a history of recurrent neck ache, pain or tension, even on pain-free days, are less accurately able to process visual and auditory information compared with a healthy control group that had no spinal problems (Farid et al. 2018). Another clinical trial has shown that a period of 12 weeks of spinal adjustments can improve the accuracy of simultaneous auditory and visual information processing in older adults (Holt et al. 2016a, b). These studies suggest that the barrage of mechanoreceptive input from paraspinal tissues after adjustments of CSMC problems can influence these brain maps. As will shortly be discussed, studies have shown that spinal manipulations in animal models appear to particularly influence the proprioceptive signalling from the deep paraspinal muscles (Pickar 2002; Pickar and Bolton 2012; Pickar and Kang 2006; Pickar et al. 2007; Pickar and Wheeler 2001). Thus, it is most likely changes in this afferent signalling that influences these various maps of the body, altering the way the brain perceives sensory information.

Several brain structures are involved in the creation of these maps, including brainstem centres, the insular cortex and other interoceptive centres, primary and secondary sensory cortices for exteroceptive inputs, frontal cortical areas, including the prefrontal cortex, as well as the cerebellum, the vestibular cortex, the autonomic ganglia and many limbic areas (Pickar 2002; Pickar and Bolton 2012; Pickar and Kang 2006; Pickar et al. 2007; Pickar and Wheeler 2001; Critchley and Harrison 2013; Kassab and Alexandre 2015; Lucci and Pazzaglia 2015; Craig 2002, 2003; Craig and Craig 2009). Together, these areas are critical for coordinated everyday movements of all kinds, as well as a host of other functions, such as homeostatic regulation of the body, how you feel emotionally, how your body functions and feels, and they even influence your motivations and behaviours (Pickar 2002; Pickar and Bolton 2012; Pickar and Kang 2006; Pickar et al. 2007; Pickar and Wheeler 2001; Critchley and Harrison 2013; Kassab and Alexandre 2015; Lucci and Pazzaglia 2015). The following sections of this review will focus on the evidence we have to date about how vertebral column dysfunction and spinal adjustments or spinal manipulation impact the various sensory organs in the paraspinal tissues (muscles and other connective tissues).

### Altered deep muscle mechanoreceptive afferents due to vertebral dysfunction, spinal adjustments or manipulation

The ability to accurately sense self-position (joint position sense) and movement (kinaesthesia) in the absence of visual inputs (Sherrington 1952; Gilman 2002), known as proprioception, is an important component of sensorimotor integration and multimodal integration within the CNS and is therefore vital for the creation of the various brain maps and the inner body schema (see Fig. 1) (Johnson et al. 2008; Proske and Gandevia 2012). Recently, the proprioceptive system has also been recognised as playing a role beyond postural and movement control (Bornstein et al. 2021). In particular, the proprioceptive system has been implicated in musculoskeletal biology and development, such as regulating spinal alignment and joint development (Bornstein et al. 2021). Of particular importance is the presence and number of the major mechanotransducers of mammalian proprioceptors, the ion channel called Piezo2 (Bornstein et al. 2021), which are known to be expressed in dorsal root ganglia neurons with muscle spindle and GTO endings (Woo et al. 2015). Muscle spindles (tiny stretch receptors within muscles), mechanoreceptors in joint capsules and cutaneous tactile receptors are sources of afferent information required for accurate joint position sense (Blum et al. 2017; Brumagne et al. 2000; Burgess et al. 1982; Cordo et al. 2002; Gilman 2002). Proprioceptive sensory information to the CNS is essential for coordinating appropriate motor output and plays an essential role during motor learning and adaptation (Bosco and Poppele 2001). Disruption in proprioceptive feedback affects the ability to predict and correct errors during movement, leading to severe defects in fine motor control without affecting the ability to move, as shown by animal studies where sensory neurons have been genetically or surgically ablated (also see Fig. 1) (Freeman and Wyke 1966), and human studies where patients have sensory neuropathies (Bosco and Poppele 2001; Ghez et al. 1995; Gordon et al. 1995). Animal studies have also shown that proprioception is important for inter-joint limb coordination, as well as the ability to adapt locomotor behaviours when confronted with uneven terrains (Abelew et al. 2000; Akay et al. 2014; Windhorst 2007). The influence of vertebral proprioceptive information has recently been proposed to play a vital role in the cortical reorganisation that has been shown to occur in people with chronic low back pain (Meier, Vrana, and Schweinhardt 2018). Such chronic pain conditions may partially be maintained because the CNS controls the movement patterns of the body incorrectly based on an inaccurate body schema, and maladaptive central neural plastic changes that occur due to this, such as central sensitisation and maladaptations within cortico-limbic and spinal circuitry.

Muscle spindles, specifically in the deep, small intervertebral muscles, are essential for how the brain controls posture and vertebral movement patterns (Du Rose and Breen 2016; Park et al. 2017). Therefore, muscle spindles are considered to play a critical role in establishing a CSMC problem. If the CNS is unable to accurately sense the current location and movement of a part of the vertebral column, it will also be unable to appropriately control the movement pattern of that part of the vertebral column. Once a central segmental motor control problem exists, and the CNS is not able to accurately perceive where the vertebral structures are, then the CNS will not be able to feedforward-activate the spine appropriately, nor appropriately integrate expected sensory responses with efference copies or with the actual sensory feedback generated from a vertebral movement, and the actual sensory feedback generated by the movements is likely to lead to increased error signals (see Fig. 1). Altered proprioceptive input from the deep paraspinal muscles, once a central segmental motor control problem is established, is therefore likely to be enough to maintain the abnormal central segmental movement pattern of that part of the vertebral column (see Fig. 2). Animal studies have shown that, in particular, deep paraspinal muscle spindle afferent input is very sensitive to vertebral movement and that zygapophyseal joint afferents are not particularly sensitive to such movements (Bolton and Holland 1996, 1998). This suggests that deep, intervertebral muscle spindle afferent input is the main source of altered afferent input that arises from and maintains the central segmental motor control problem.

Proprioceptive information from the paraspinal muscles has also been found to be particularly vital for maintaining the alignment of the vertebral column (Blecher et al. 2017). For instance, it has been found that people with adolescent idiopathic scoliosis have reduced muscle spindle concentration in their paraspinal muscles (Ford et al. 1988). For any movement of the body to take place, the CNS relies on somatosensory information to define the starting posture of the body and to monitor the progress of the movement in order to perform corrections to the movement as it takes place (Fiehler et al. 2004; Simoneau et al. 1995). Thus, for all weight-bearing movements, the CNS needs to feedforward activate protective core muscles to stabilise the vertebral column and prevent loss of balance (Allison et al. 2008; Cavallari et al. 2016; Fujiwara et al. 2003; Gibson and McCarron 2004; Santos et al. 2010), all of which require the CNS to know what is going on at the level of the vertebral column (see Fig. 1). This feedforward activation of core muscles is referred to as anticipatory postural adjustments which are made by the CNS for the maintenance of balance and protection of the vertebral column (Klous et al. 2011; Piscitelli et al. 2017). These anticipatory postural adjustments will also adapt and change depending on how much you choose to move and exercise (Yiou et al. 2012). Most of the required somatosensory information for such postural adaptations is derived from proprioceptors within deep paraspinal muscles (Amonoo-Kuofi 1983; Blecher et al. 2017; Boyd-Clark et al. 2002; Cooper and Daniel 1963; Kulkarni et al. 2001; Loeb et al. 1999), making the signalling from these deep paraspinal muscles vitally important (see Fig. 1).

The deep paraspinal muscles play a significant role in proprioception. This is because they are rich in muscle spindles, particularly the upper cervical or suboccipital deep paraspinal muscles which have a high concentration and density of muscle spindles and motor units (Amonoo-Kuofi 1983; Kulkarni et al. 2001; Boyd-Clark et al. 2002; Cooper and Daniel 1963). High proprioceptive content makes them ideal for sensing position and movement of craniovertebral joints (Kulkarni et al. 2001). Large spindle densities have been found in small muscles required for fine motor control, while those recruited for gross movement have comparatively lower spindle density (Boyd-Clark et al. 2002). In addition, the lack of tendon organs in suboccipital muscles makes them functionally capable of sensing length changes, i.e. they sense movement but not contractile tensions (Kulkarni et al. 2001). However, muscle spindle characteristics represent only one aspect of the many factors contributing to proprioceptive regulation in skeletal muscle (Boyd-Clark et al. 2002). Due to the known convergence of inputs from the neck proprioceptors, vestibular, oculomotor and visual system at various levels of the neuroaxis, the sense of movement from the suboccipital muscles is likely to be handled in a very complex manner (Kulkarni, Chandy, and Babu 2001).

Maladaptive plastic changes in the deep paraspinal muscles are known to occur with spinal injury (Brown et al. 2011; Hodges et al. 2006, 2009, 2014, 2015; James et al. 2016). Early on, after experimentally induced disc injury in animal models, the deep paraspinal muscles, such as the multifidi, undergo rapid atrophy due to neural inhibition (Hodges et al. 2006, 2009). During the subacute to early chronic period, these deep muscles have been shown to undergo additional maladaptive bioplastic changes, such as a development of muscle fibrosis, extensive fatty infiltration and changes in muscle fibre types, from slow-to-fast twitch (Brown et al. 2011; Hodges et al. 2014, 2015; James et al. 2016). Human studies have also shown early multifidus muscle atrophy (Hides et al. 1994), and later, fatty infiltration has been found (Alaranta et al. 1993; Fortin et al. 2016). Similarly, in herniated disc patients, multifidus muscle atrophy accompanies chronic disc degeneration (Zhao et al. 2000). As mentioned, these paraspinal muscle changes are likely to be driving the recurrence and chronification of back pain though maladaptive central neural mechanisms (et al. 2016; Chang et al. 2019; Meier et al. 2018). It is thought that these adapted motor control strategies might have long-term consequences, such as increased spinal loading that has been linked with degeneration of intervertebral discs and other tissues, potentially maintaining some types of recurrent or chronic low back pain (Meier et al. 2018). Regardless, there is clear evidence that maladaptive dysfunction of the deep paraspinal muscles can occur (Brown et al. 2011; Hodges et al. 2006, 2009, 2014, 2015; James et al. 2016), which is likely to reduce the ability of the CNS to accurately perceive what is going on at that level of the vertebral column, which over time is reflected in the blurring of the sensorimotor cortical areas (Burns et al. 2016; Chang et al. 2019), which is likely to lead to poor vertebral motor control, maintaining a central segmental motor control problem.

There is strong evidence of impaired vertebral proprioception in chronic, idiopathic neck pain and low back pain patients from systematic reviews and meta-analyses (Stanton et al. 2016; Tong et al. 2017). Multiple studies have shown that people with spinal dysfunction to the point of having chronic neck pain of no apparent cause are worse than asymptomatic controls at head-to-neutral repositioning tests (Stanton et al. 2016). This is likely due to changed proprioceptive processing due to altered afferent input from the neck from the paraspinal tissues and/or altered multimodal integration of this afferent information due to the pain itself, and is most likely a combination of both (Stanton et al. 2016). Interestingly, although muscle vibration studies, selectively activating the muscle spindles within muscles (Burke et al. 1976), reduces the accuracy of vertebral joint position sense in healthy participants, it actually improves the vertebral joint position sense acuity in people with neck pain (Beinert et al. 2015), people with low back pain (Brumagne et al. 2000), and even in subclinical neck pain patients (Paulus and Brumagne 2008). This may be because previous spinal trauma, pain, inflammation or psychological stress has led to the development of the neck and/or low back pain, partially because of the changes in afferent signalling from the deep paraspinal muscles or the changes of central processing of their muscle spindle information (Butler and Moseley 2003; Hellström et al. 2005; Passatore and Roatta 2006; Brown et al. 2011; Hodges et al. 2006, 2009, 2014, 2015; James et al. 2016; Le Pera et al. 2001). Physical injury to the spinal disc can, as mentioned earlier, lead to atrophy of the deep paraspinal muscles (Brown et al. 2011; Hodges et al. 2006, 2009, 2014, 2015; James et al. 2016). Also, as mentioned earlier, scientists have suggested that disrupted or reduced proprioceptive signalling from deep paraspinal muscles likely plays a pivotal role in driving the long-term cortical reorganisation and changes in the top-down control of the sensorimotor systems and that this plays a vital role in driving the recurrence and chronicity of back pain (Meier et al. 2018). If there is a reduction in proprioceptive signalling from the deep paraspinal muscles, this would explain the poor spinal joint position sense acuity that is found in neck and low back pain populations (Stanton et al. 2016; Tong et al. 2017), and it would also explain why spinal proprioception is improved by vibration which is an effective method of selectively activating the muscle spindles (Burke et al. 1976). Spinal manipulation also appears to selectively impact the muscle spindle activity of the deep paraspinal muscles and can improve vertebral column proprioception, both of which will be discussed in greater detail shortly. This may explain why spinal adjustments of CSMC problems have a more significant impact on neuromuscular function than spinal manipulation of vertebral segments that lack clinical indicators of dysfunction. If CSMC problems are associated with atrophied paraspinal muscles, applying an HVLA thrust directed at these segments may stimulate these muscles back into a better functioning state. This should be studied further in future studies. In summary, the vertebral column should be viewed as a functional unit, and any change in muscle feedback in any part of the column will impact on other parts of the unit. This is similar to the ‘referred pain’ phenomenon. Pain causing changes in, let us say the thoracic section of the unit, may reflect to the part that is most heavily used and hence most vulnerable to fatigue and pain (i.e. neck and low back sections of the column).

Multiple studies have explored the neurophysiological impacts of an HVLA thrust applied to the vertebral column (see Table 2) (Gyer et al. 2019; Pickar 2002; Taylor et al. 2010; Wirth et al. 2019; Cao and Pickar 2014; Reed et al. 2013a, b, 2017a, b; Clark et al. 2011). Animal studies that have explored the effects of spinal adjustments and spinal manipulation have provided both direct and indirect measures of altered proprioception (Cao et al. 2013; Pickar and Kang 2006; Pickar et al. 2007; Pickar and Wheeler 2001; Sung et al. 2005; Reed et al. 2013a, b, 2014a, b, 2017a, b; Reed and Pickar 2015; Colloca et al. 2006, 2008, 2012; Song et al. 2006, 2016; Duarte et al. 2019). When an HVLA thrust is applied to the vertebral column, this will stretch the deep paraspinal muscles. Studies have shown that muscle spindles and Golgi tendon organs with receptive endings in the paraspinal muscles in anaesthetised animals respond to vertebral loads whose force–time profiles are similar to that of a load delivered during spinal adjustments and manipulation (see Table 2) (Pickar and Bolton 2012; Pickar and Wheeler 2001; Cao and Pickar 2014; Cao et al. 2013; Pickar and Kang 2006; Pickar et al. 2007; Sung et al. 2005; Reed et al. 2013a, b, 2014a, b; 2017a, b). Studies using animal models of spinal dysfunction, including creating hypermobile and hypomobile segments, have also clearly shown that HVLA spinal adjustments of these CSMC problem segments results in changes in paraspinal muscle spindle afferents (Reed et al. 2013a, b; Reed and Pickar 2015). This suggests that it is the deep intervertebral paraspinal muscles in particular that respond to the adjustive or manipulative HVLA thrusts and that it is their proprioceptive afferent information that is signalled to the CNS during an adjustment (as depicted in Figs. 1, 2) (also see Table 2) (Pickar and Bolton 2012; Pickar and Wheeler 2001). Multiple animal and human studies have clearly shown that HVLA thrusts evoke short-lasting EMG responses in paraspinal skeletal muscle (Herzog 1996; Herzog et al. 1999; Colloca et al. 2003, 2006, 2008, 2012; Nougarou et al. 2013; Pagé et al. 2014), demonstrating that HVLA adjustments definitely have a central neuromuscular impact (as depicted in Figs. 1, 2). Thus, altering paraspinal afferent information can affect the CNS and is most likely responsible for the majority of CNS changes that occur following adjustments of CSMC problems. Colloca et al. (2008, 2012) demonstrated in multiple studies, using a chronic disc injury sheep model, that HVLA spinal adjustments of the segment adjacent to the chronic disc degeneration was associated with a reduction (20–30%) in the intramuscular EMG responses from the deep paraspinal muscles compared to spinal manipulation of spinal segments that were not identified as being dysfunctional, again highlighting a different neurophysiological effect of HVLA thrusts that either target a CSMC problem or a normally functioning vertebral segment. This must be further explored in future research, to clarify the clinical implications in various human populations.

**Table 2:**
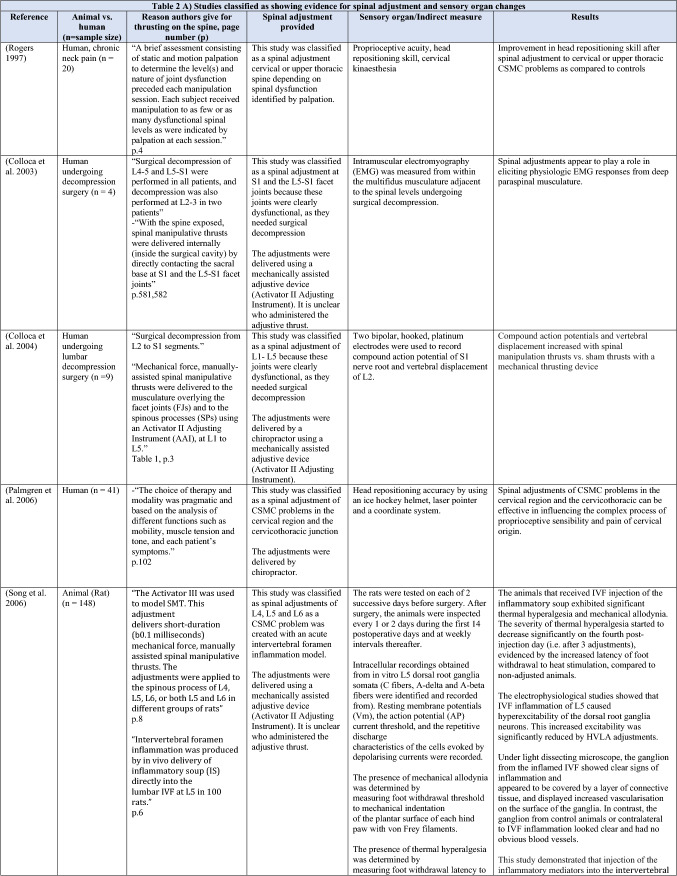

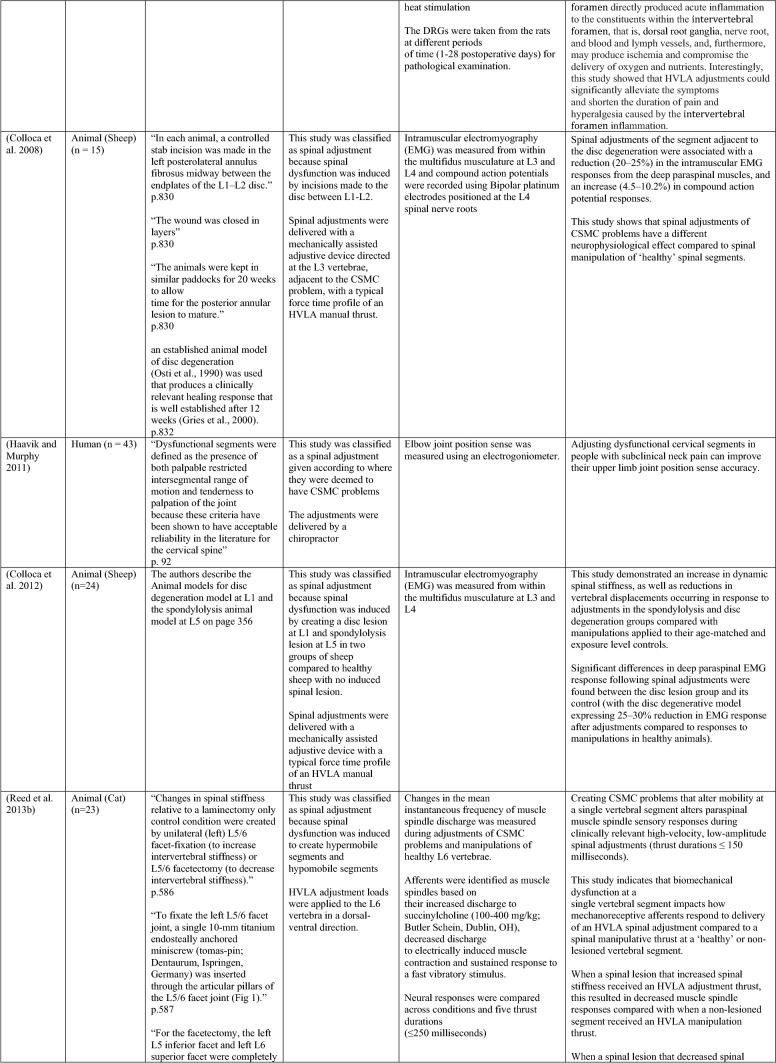

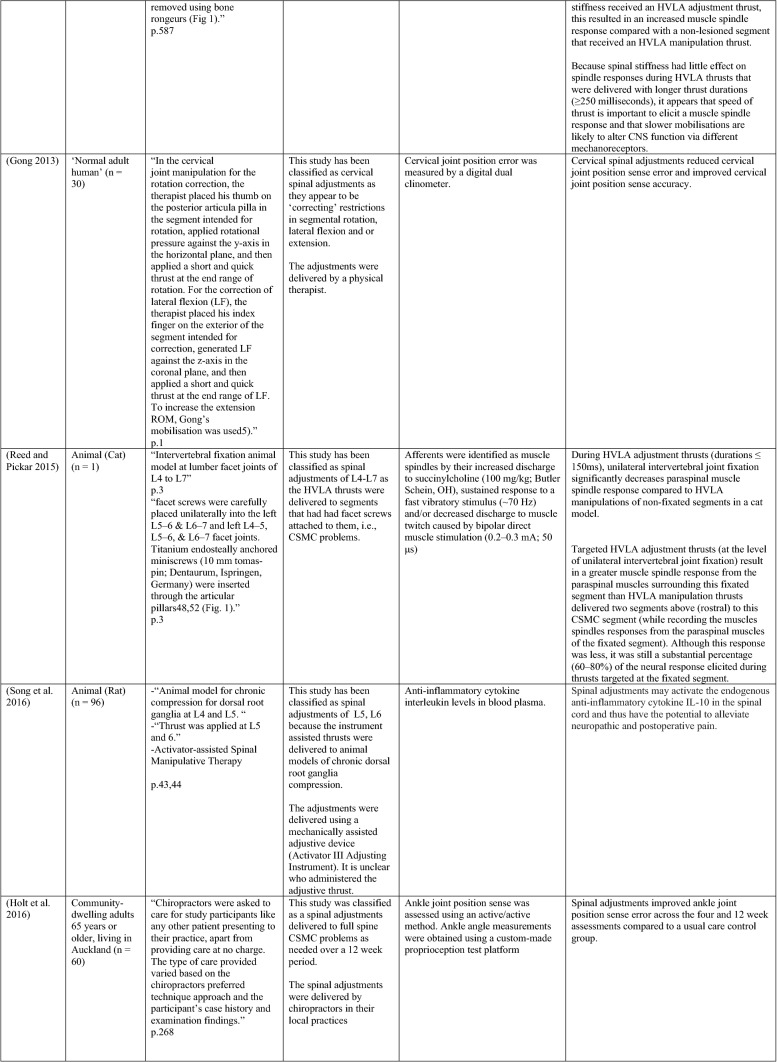

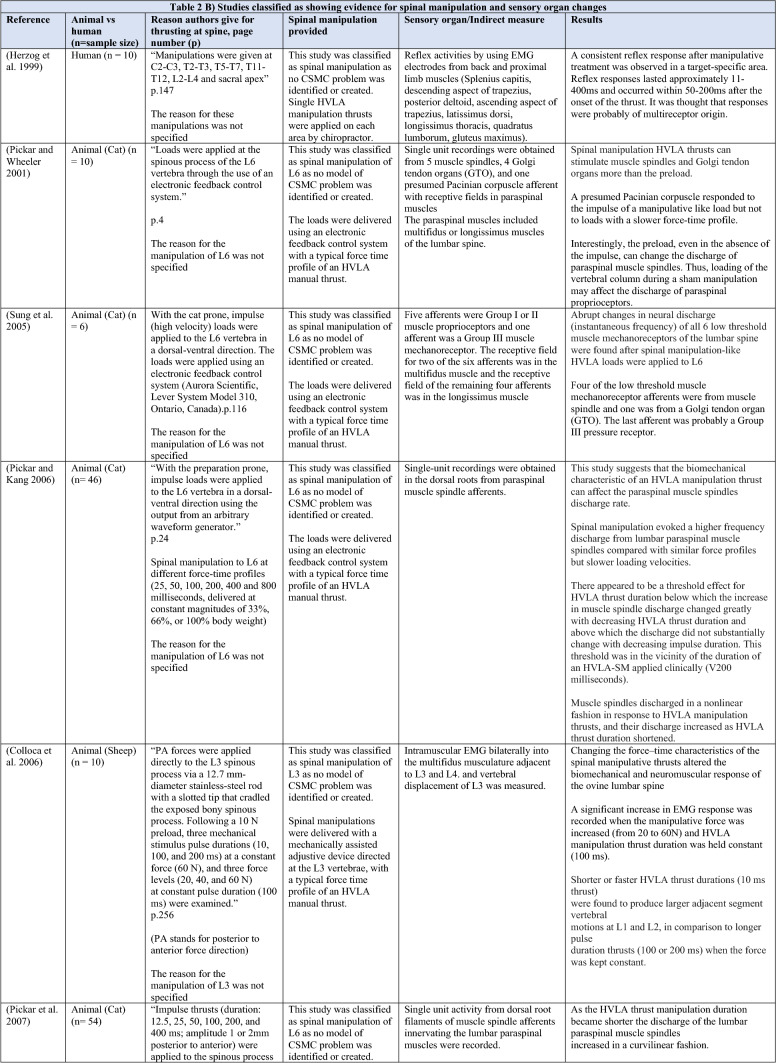

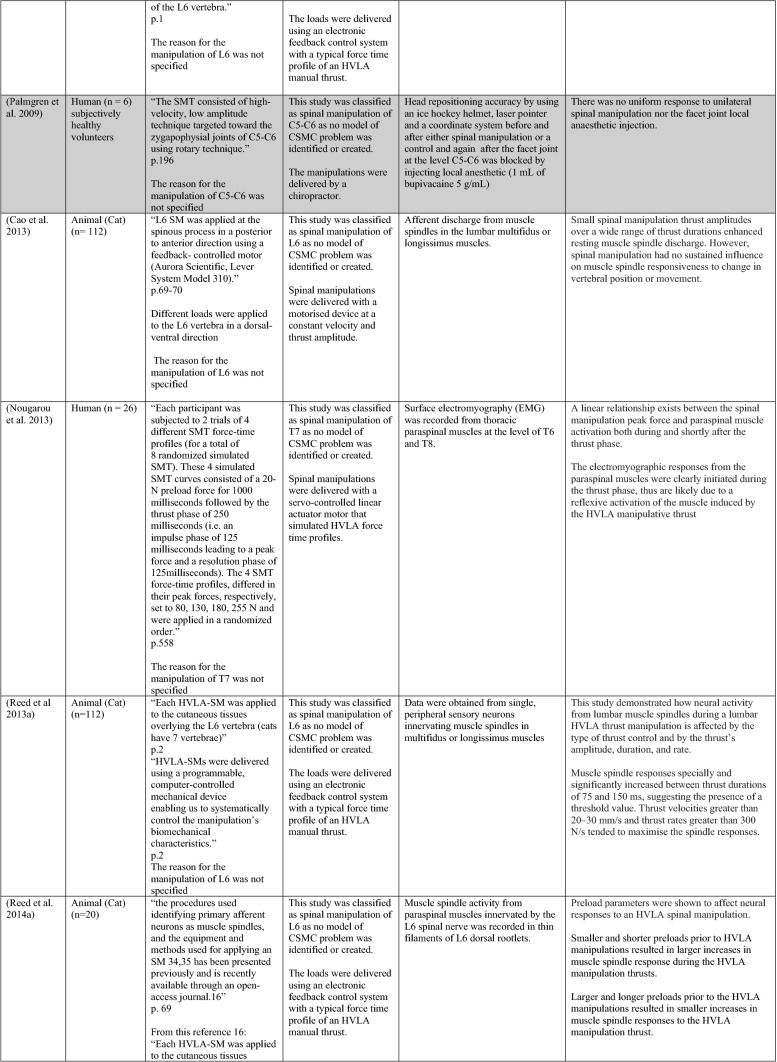

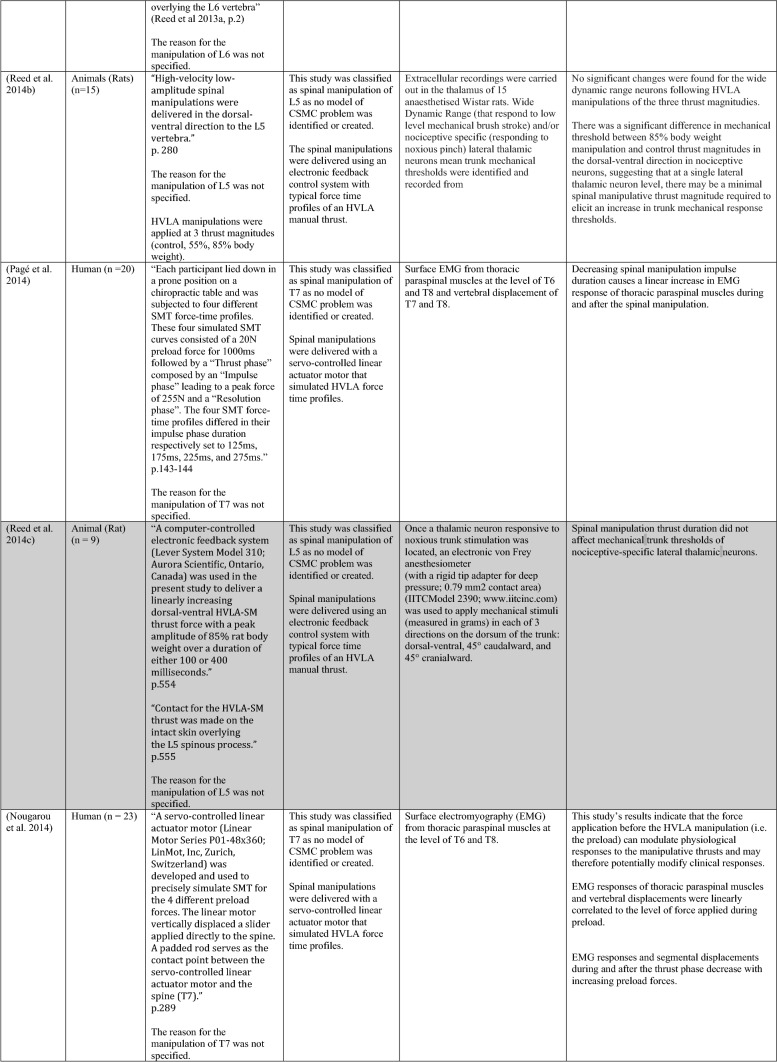

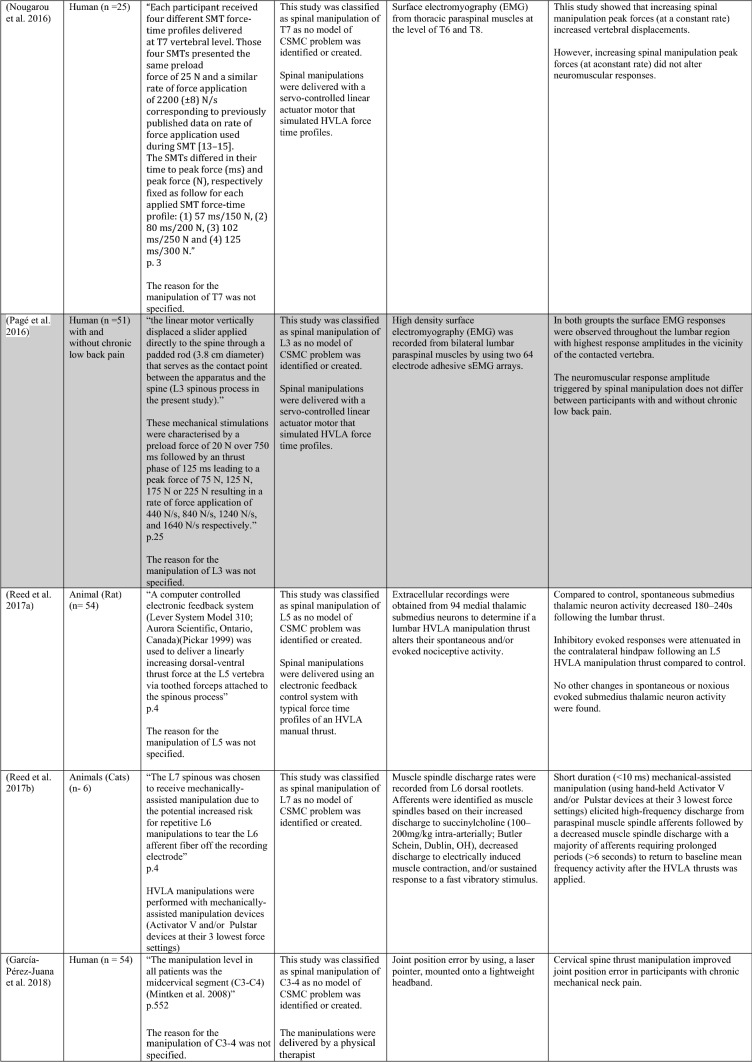

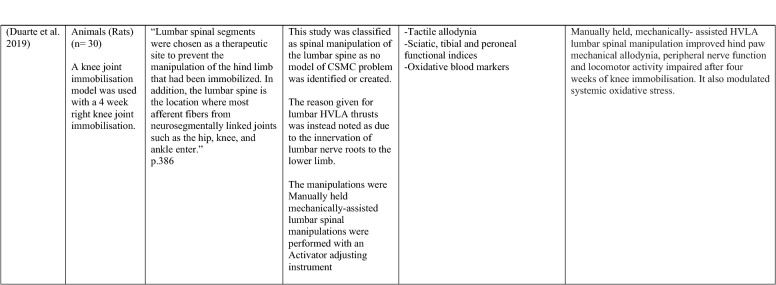
Summary of the evidence for sensory organ changes due to vertebral dysfunction, spinal adjustments or manipulation [The studies have been categorised as delivering spinal adjustment (**A**) or spinal manipulation (**B**) based on the reason authors give for thrusting on the spine]

Studies that found no change in outcome measures have been highlighted in grey. *EMG* electromyography, *HVLA* high-velocity low-amplitude thrust, *CSMC problems* central segmental motor control problems

Recently, the preliminary results were presented from a study done in a human population that investigated whether an HVLA adjustive thrust delivered with an Activator adjusting instrument to a CSMC in the cervical spine had different neurophysiological outcomes to an HVLA thrust to a segment that was deemed to be functioning normally (‘Association of Chiropractic Colleges Research Agenda Conference 2021 Abstracts of Proceedings’ 2021). The Activator hand-held adjusting instrument was used to ensure the HVLA thrusts were identical in both conditions. This study included 96 participants with evidence of a CSMC problem in their upper cervical spine and recorded the participants’ N30 SEP peak amplitudes as the primary outcome measure. The preliminary results from this study revealed a significant decrease in N30 SEP peak amplitude (*p* < 0.1, − 16.76% ± 28.32%) in the group that received the HVLA thrust directed at a CSMC problem. This decrease is similar to those previously observed following HVLA manual spinal adjustments to CSMC problems (Haavik Taylor and Murphy 2007; Lelic et al. 2016). In contrast, the thrusts delivered to a normally functioning vertebral segment (i.e. one that did not display clinical indicators of a CSMC problem) resulted in a non-significant increase (*p* = 0.4, 19.58% ± 55.09%) in N30 SEP peak amplitudes. These between group differences were significant (*p* < 0.1) and provide direct evidence that a spinal adjustive thrust from a hand-held activator adjusting instrument directed at a CSMC problem results in different neurophysiological outcomes to a similar HVLA thrust directed at a normally functioning vertebral segment in humans.

Spinal adjustments have recently also been shown to alter intersegmental range of motion (Anderst et al. 2018), which in turn will also alter the ongoing stretch of these small intervertebral muscles, thus also induce changes in the sensory signalling from this part of the vertebral column even after the adjustment ends. Knowing this, and considering that both the velocity and relative position of the vertebral displacement appears to be encoded by afferent nerve activity from intervertebral muscles and that afferent nerves innervating the zygapophyseal joints do not contribute significantly to the signalling of vertebral displacement (Bolton and Holland 1996, 1998), all this supports the contemporary view that CSMC problems lead to ongoing altered input from mechanoreceptors, such as muscle spindles and Golgi tendon organs, from the deep intervertebral muscle afferents to the CNS and that the main impact of spinal adjustments and manipulation is also due to altered afferent input from muscle spindles and Golgi tendon organs, from the deep intervertebral muscle both during and after the controlled HVLA vertebral thrusts (Alcantara et al. 2013; Haavik and Murphy 2012; Henderson 2012; Kent 1996; Taylor et al. 2010; Reed et al. 2017a, b; Cao and Pickar 2014). Further evidence for this model comes from studies that have shown proprioceptive improvements occur following spinal adjustments (Haavik and Murphy 2011; Holt et al. 2016a, b; Palmgren et al. 2006, 2009). Improved head repositioning accuracy has been demonstrated several times following chiropractic care (Palmgren et al. 2006, 2009; Rogers 1997; Gong 2013; García-Pérez-Juana et al. 2018), suggesting that spinal adjustments can improve vertebral column proprioception.

The two studies using animal models of CSMC problems demonstrated there are differences in proprioceptive afferent input to the CNS from HVLA adjustments of these dysfunctional segments compared to HVLA manipulation thrusts at non-lesioned vertebral segments (Reed and Pickar 2015; Reed et al. 2013a, b). For example, Reed et al. 2013a, b demonstrated that induced biomechanical dysfunction at a single vertebral segment impacts how mechanoreceptive afferents respond to delivery of an HVLA spinal adjustment compared to a spinal manipulative thrust at a non-lesioned vertebral segment. When a spinal lesion that increased spinal stiffness received an HVLA adjustment thrust this resulted in decreased muscle spindle responses compared with when a non-lesioned segment received an HVLA manipulation thrust (Reed et al. 2013a, b). Furthermore, when a spinal lesion that decreased spinal stiffness received an HVLA adjustment thrust this resulted in an increased muscle spindle response compared with a non-lesioned segment that received an HVLA manipulation thrust (Reed et al. 2013a, b). As the authors highlight, this is relevant to clinical practice, as both increased or decreased mobility of a spinal segment appears capable of altering CNS afferent input, and adjusting either of these will have different effects on the neural input to the CNS during the adjustment (Reed et al. 2013a, b). In another study, also using an animal model that created CSMC problems by fixating lumbar facet joints of anaesthetised cats, Reed and Pickar (2015) demonstrated that HVLA adjustment thrusts (with durations ≤ 150 ms) significantly decreased paraspinal muscle spindle responses compared to HVLA manipulations of non-fixated segments. This is interesting as it clearly demonstrates that adjustments of dysfunctional segments will have a different neurophysiological effects on neural input to the CNS compared to manipulating non-lesioned segments. It makes sense that fixated or hypomobile vertebral segments would result in smaller paraspinal muscle spindle responses, compared with manipulating more freely moving vertebral segments. The central and clinical effects of this must be further studied, to clarify what this means for movement, function and neuromuscular control in humans.

Reed and Pickar (2015) also noted that HVLA adjustment thrusts targeted at the level of unilateral intervertebral joint fixation resulted in a greater muscle spindle response from the paraspinal muscles surrounding this fixated segment compared with HVLA manipulation thrusts delivered 2 segments above (rostral) the CSMC segment (while recording the muscles spindles responses from the paraspinal muscles of the fixated segment). Although the response from the HVLA manipulation of non-fixated segment, two segments above the fixated segment, resulted in a smaller muscle spindle afferent response at the fixated level, it was still a substantial percentage of 60–80% of the neural response elicited during thrusts targeted at the fixated segment itself. This has clinical implications, as also highlighted by the authors, as it suggests that even if you target a vertebral level above (and also possibly below) a hypomobile segment, you will still activate a significant portion of the muscle spindles at the fixated vertebral segment (Reed and Pickar 2015). This indicates that an adjustment targeted at the CSMC problem segment would have a greater neural afferent impact than a general manipulation that thrusts at levels surrounding the fixated segment, but not specifically targeting the fixated segment. Reed et al. (2013a, b) also noted that varying spinal stiffness levels prior to the HVLA thrusts had little effect on spindle responses during HVLA thrusts that were delivered with longer thrust durations (≥ 250 ms) (Reed et al. 2013a, b). Thus, it appears that speed of thrust is important to elicit a muscle spindle response, and that slower mobilisations are therefore likely to alter CNS function via different mechanoreceptors (Reed et al. 2013a, b).

Multiple other studies have confirmed that speed of HVLA thrust greatly impacts muscle spindle responses during a manipulation (Reed et al. 2013a, b; Reed and Pickar 2015; Pickar and Kang 2006; Pickar et al. 2007). Even the preload, prior to the HVLA thrust appears to impact the neural afferent response of muscles spindles in the paraspinal tissues to the HVLA manipulation (Pickar and Wheeler 2001; Reed et al. 2014a, b). Smaller and shorter preloads prior to HVLA manipulations have been shown to result in larger increases in muscle spindle response during the HVLA manipulation thrusts, with larger and longer preloads prior to the HVLA manipulations resulting in smaller increases in muscle spindle responses to the HVLA manipulation thrust (Reed et al. 2014a, b). Cao et al. (2013) showed that HVLA spinal manipulation of L6 in anaesthetised cats had no sustained influence on muscle spindle responsiveness to changes in vertebral position or movement after the manipulation. It is possible this is because the manipulations were applied to non-lesioned vertebral segments. Future studies should explore if HVLA adjustments of CSMC problems, for example in animal models of chronic disc injuries, leads to sustained changes in muscle spindle afferent input after the adjustment. Anderst’s et al. (2018) recent study supports this notion, as they demonstrated in humans that spinal adjustments alter the intersegmental range of motion of vertebral segments after the adjustment.

All of the above animal studies suggest that rapid stretch of the deep, small paraspinal muscles, that occurs during an HVLA adjustment, play a major role in the mechanisms of spinal adjustments, by bombarding the CNS with mechanoreceptive and proprioceptive input (Boal and Gillette 2004; Evans 2002; Haavik and Murphy 2012; Haavik 2014; Henderson 2012; Pickar 2002; Pickar and Bolton 2012; Potter et al. 2013; Cao et al. 2013; Pickar and Wheeler 2001; Pickar and Kang 2006; Pickar et al. 2007; Sung et al. 2005; Reed et al. 2013a, b, 2014a, b, 2017a, b; Reed and Pickar 2015). This explains why spinal adjustments improve vertebral column proprioception (Palmgren et al. 2006, 2009; Rogers 1997; Gong 2013; García-Pérez-Juana et al. 2018). Interestingly, not only do spinal adjustments improve vertebral column proprioception, but studies have shown that spinal adjustments also can improve upper and lower limb proprioception (Haavik and Murphy 2011; Holt et al. 2016a, b). Thus, this mechanoreceptive blast to the CNS from the spine appears to change the accuracy by which the CNS perceives where the spinal structures are and can also improve the accuracy by which the CNS perceives where the limbs are, and/or improves the CNS’s ability to feedforward activate the spine appropriately, and/or improves the way the CNS integrates expected sensory responses with efference copies with the actual sensory feedback generated from a vertebral movement, enabling it to more appropriately error check and correct movements as they are occurring (as depicted in Fig. 1).

The effects of improving vertebral motion segment function on upper limb proprioception was investigated in 25 participants with SCSP compared to 18 healthy control participants (Haavik and Murphy 2011). It was found that the SCSP group had reduced elbow joint position sense compared with those who had no history of any neck complaints (Haavik and Murphy 2011). Application of cervical spine adjustments of CSMC problems improved the accuracy of the SCSP groups’ elbow joint position sense (Haavik and Murphy 2011). This suggests that neck dysfunction, to the point of experiencing recurring neck ache, pain or tension, can impair the way that proprioceptive information from the upper limb is processed. It also suggests that improving vertebral motion segment function with spinal adjustments leads to more appropriate and accurate processing and integration of such proprioceptive input. This notion was supported by another study that explored ankle proprioception in 60 older adults, which showed that after both 4 and 12 weeks of chiropractic care, that consisted of adjusting CSMC problems, there was an improvement in their ankle joint position sense (Holt et al. 2016a, b). It is important to note that the participants’ ankles were not adjusted in this study (Holt et al. 2016a, b), which again suggests that vertebral column function impacts the brain’s inner maps of the body and influences the way the brain perceives proprioceptive information from the limbs. Traditionally, the main focus of chiropractic care has been to locate, analyse and correct CSMC problems (Rosner 2016). These studies suggest that the vertebral column likely acts as a core reference point for limb motor control (Haavik and Murphy 2011; Holt et al. 2016a, b), and this may be why good spinal function is vital (Bellan et al. 2017).

On a final note, it is essential also to recognise that recent research suggests that the proprioceptive system seems to be important for far more than just the control and coordination of movement and posture (Bornstein et al. 2021). Bornstein et al. (2021) have recently highlighted that the proprioceptive system is also implicated in regulating a wide range of developmental and physiological processes (Bornstein et al. 2021). For example, they discuss the role and function of the Piezo2 ion channel that is the major mechanotransducer of mammalian proprioceptors, including proprioceptors found in neurons ending in muscle spindles and GTOs (Bornstein et al. 2021). In particular, they note that the loss of Piezo2 ion channels in animal research can lead to spinal malalignment (equivalent to scoliosis in humans) and hip dysplasia and suggest that future research is likely to identify additional aspects of musculoskeletal biology that is regulated by the proprioceptive system (Bornstein et al. 2021). Furthermore, they discuss that the loss of proprioception, that we have suggested occurs with CSMC problems, can lead to uncoordinated movement and altered muscle activation patterns, which in turn can lead to abnormal stressors on joints and that “such abnormal stressors on joints can, in turn, lead to abnormal mechanical signals in joint cells, affecting joint integrity and resulting in aberrant joint morphology” (Bornstein et al. 2021, p.85). This novel research supports the wider implications of the contemporary model of CSMC problems depicted in Fig. 2 of this review. It also highlights the need for further investigation of the physiological and clinical implications of the CSMC problem and spinal adjustment in future studies.

In summary, accurate proprioception is crucial for sensorimotor integration, multimodal integration and the creation of inner body and external world schema within the CNS. This is because proprioceptive sensory inputs are responsible for coordinating motor outputs, predicting accurate movement and correcting errors. Disruption in proprioceptive inputs causes severe deficits in motor control, inter-joint coordination, motor learning and adaptation without affecting the ability to move (Bosco and Poppele 2001; Ghez et al. 1995; Gordon et al. 1995; Abelew et al. 2000; Akay et al. 2014; Windhorst 2007; Freeman and Wyke 1966). Proprioceptive inputs from the spine play an important role in postural control (Blecher et al. 2017; Cavallari et al. 2016). Figure 1 depicts the role of the afferent input from the deep paraspinal muscles and how it can alter neuromuscular function in multiple ways, by impacting the motor plan itself, the motor command messages, the predicted sensory feedback the CNS will expect and therefore the integration of the predicted and actual sensory feedback created by the moving muscles as well as feedforward postural control of the vertebral column. The small and deep paraspinal muscles of the spine, rich in muscle spindles, are responsible for maintaining spinal alignment and feedforward activation of the spine (Amonoo-Kuofi 1983; Blecher et al. 2017; Boyd-Clark et al. 2002; Cavallari et al. 2016). Altered proprioceptive input from paraspinal muscles around CSMC problems leads to maladaptive changes within the CNS as it cannot accurately perceive what is going on at the level of the spine (see Fig. 1). Application of spinal adjustments rapidly stretches the deep, small paraspinal muscles so that the CNS is bombarded with mechanoreceptive and proprioceptive inputs (see Fig. 1) (Boal and Gillette 2004; Evans 2002; Haavik and Murphy 2012; Haavik 2014; Henderson 2012; Pickar 2002; Pickar and Bolton 2012; Potter et al. 2013). This changes the accuracy by which the CNS perceives the position of the vertebrae and improves the ability to produce feedforward postural adjustments, thus explaining the mechanism of the correction of CSMC problems by spinal adjustments. The impacts of spinal manipulation of vertebral column segments that are not dysfunctional are therefore likely to be different as the paraspinal muscles surrounding a vertebral segment that is functioning well will not be atrophied, have fatty infiltration, be fibrous or have changes in muscle fibre types that occur with CSMC problems. Thrusting on a vertebral segment that is not dysfunctional may still influence the CNS, but is likely to have reduced neurophysiological effects compared to those that occur when a CSMC problem is adjusted. This needs further investigation in future studies.

### Altered skin and joint receptors due to vertebral dysfunction, spinal adjustments or manipulation

Vertebral column motor control can be altered by various conditions such as physical injury, pain, inflammation and acute or chronic physiological or psychological stress (Le Pera et al. 2001; Thunberg et al. 2001). As discussed, both CSMC problems and spinal adjustments and manipulation are known to involve changes in deep muscle mechanoreceptive afferent input to the CNS (see Table 2). However, there are other sensory organs in the paraspinal tissues that may also be changed due to vertebral dysfunction and/or spinal adjustments and/or manipulations. Injury to the vertebral column is known to cause local inflammation, for example, which would also be signalled to the CNS via slow-conducting (group III and IV) afferents in the paravertebral tissues (Grigg et al. 1986). It has been shown that patients with non-specific acute and chronic low back pain have elevated levels of some inflammatory mediators compared to control subjects (Colombi and Testa 2019; Teodorczyk-Injeyan et al. 2018). For instance, increased levels of inflammatory mediators such as tumour necrosis factor α, interleukin-1, and the neurotransmitter substance P have been noted in people with discogenic back pain (Burke et al. 2002). Post-spinal injury inflammation not only changes the muscle afferent input from the deep paraspinal muscles, but also changes other slow-conducting (group III and IV) afferent input from the zygapophyseal joints and paravertebral tissues (Le Pera et al. 2001; Thunberg et al. 2001). There is some evidence that the application of mechanical forces on zygapophyseal joints, para-articular tissues, or both can activate such neurons (see Table 2) (Bogduk and Marsland 1988). Some of these high-threshold, mechanical afferents (group IV) could be relaying signals that result in the sensation of pain due to vertebral joint injury or inflammation (Cavanaugh et al. 2006; Bogduk and Marsland 1988) thus they may also impact motor control. It is clear from multiple studies that any changes in afferent input from joint ligaments, capsules, fascia and deep intervertebral muscles are all critically involved in the central motor control of the spines stabilising muscles (Benjamin 2009; Holm et al. 2002; Kang et al. 2002; Le et al. 2009; Loeb et al. 1999; Stubbs et al. 1998; Yahia et al. 1992). More specifically, both pain and inflammation are well known in the literature to alter neuromuscular function in a variety of ways, and result in maladaptive changes in motor control (Brumagne et al. 2019; Hodges and Moseley 2003; Hodges and Tucker 2011; Hodges et al. 2019; Jull and Richardson 2000; van Vliet and Heneghan 2006; van Dieën et al. 2018). Aging may reduce the capacity of the joint receptors in the vertebral column to signal proprioceptive information to the CNS due to calcification of the joint surfaces, hence altering the cortical body image.

A recent study has provided support for the proposal that macrophages and tumour necrosis factor (TNF), a pro-inflammatory cytokine, play an active role in the subacute and early chronic phase of the known maladaptive plastic changes of the deep paraspinal muscles following disc injuries (James et al. 2018). Macrophages are known to regulate inflammation, tissue integrity and pain after muscle injury (Gong et al. 2016; Gregory et al. 2016; Leung et al. 2016). Through cytokine expression, macrophage subtypes affect collagen synthesis and other processes that regulate muscle structure (Mann et al. 2011; Villalta et al. 2009; Wehling-Henricks et al. 2010). The two main subtypes of macrophages are called M1, which are pro-inflammatory, and M2, which are anti-inflammatory, and both contribute at different times during healing. In animal models of experimental intervertebral disc injury, there is no direct injury to the deep paraspinal muscles themselves, yet pro-inflammatory cytokine gene expression and structural maladaptations do occur (Hodges et al. 2014, 2015; James et al. 2016). A greater proportion of M1 macrophages are found in such paraspinal muscles 3 and 6 months after experimental disc lesions (James et al. 2018). A greater proportion of M1 macrophages are also found in adipose tissue 6 months after disc injury. It is, therefore, possible that modified macrophage subtype and cytokine expression may provide a novel explanation for the dramatic muscle changes that have been observed after experimentally induced disc injury (James et al. 2018). Much work is still needed in this area. For example, if spinal adjustments do improve deep paraspinal muscle function, then this should be measurable using the same disc injury animal models that have shown how these muscles dysfunction over time (Brown et al. 2011; Hodges et al. 2014, 2015; James et al. 2016). Adjusting the injured segment should, in theory, prevent these maladaptive plastic changes from taking place or should reverse them once they have happened. Using these animal models of disc injury, it would be possible to measure the degree of deep paraspinal muscle atrophy, muscle fibrosis, fatty infiltration and changes in muscle fibre types, from slow-to-fast twitch (Brown et al. 2011; Hodges et al. 2014, 2015; James et al. 2016) and compare those animals who received spinal adjustments around the injured segment, or manipulations at a non-lesioned vertebral level compared to no HVLA thrusts provided. Macrophage types and concentrations could also be recorded. These types of studies would elucidate the impact of spinal adjustment vs manipulation and provide evidence regarding their application for paraspinal muscle function.

The most convincing favourable inter-practitioner reliability evidence for identifying CSMC problems in a clinical setting is the elicitation of pain or tenderness when a practitioner presses gently over the specific vertebral level or region (Triano et al. 2013). Most studies show that pain or ‘tenderness to touch’ can be reliably elicited, which includes tenderness to palpation of spinous and transverse processes, as well as identifying larger areas that are painful (Triano et al. 2013). This suggests that locally, at the area of a CSMC problem, where the CNS may not be fully aware of what is going on and therefore is not accurately controlling the movement pattern appropriately, there may be higher levels of inflammation present, that makes this part of the spine tender to touch. This may be because the CNS is not accurately aware of the movement from this particular vertebral segment (Tresch et al. 2002), thus the CNS may be controlling that part of the spine in an abnormal way (Nava and Röder 2011), which regularly causes microtrauma at that level of the spine, which is enough to cause increased levels of local inflammation that elicit pain or tenderness upon touch or the application of slight mechanical pressure (Keating et al. 2001). Increased local inflammation has been a part of CSMC problem theories for many decades (Kent 1996), and there is some evidence that supports this. It has been found that people with non-specific acute or chronic low back pain have a greater concentration of some inflammatory mediators as compared to control groups (Colombi and Testa 2019; Teodorczyk-Injeyan et al. 2018). This may be due to the microtrauma-induced inflammation at the level of CSMC problems or spinal dysfunction.

Considering that animal models of intervertebral disc injury show greater gene expression of pro-inflammatory cytokines, such as TNF and interleukin-1β, within the deep paraspinal multifidus muscles (Hodges et al. 2014, 2015; James et al. 2016), these studies implicate the activity of such proinflammatory mediators in the pathogenesis of back pain that is due to intervertebral disc degeneration (Burke et al. 2002; Hodges et al. 2014, 2015; James et al. 2016). In addition, it is known that these mediators are important for the regulation of inflammatory responses at both local and systemic levels (Lotz et al. 1988; Metwali et al. 2004; Suffredini et al. 1999). Further evidence for disc degeneration increasing local inflammation in the surrounding tissues, including the deep paraspinal muscles, is provided by studies that have found higher disc degeneration rates at the vertebral levels next to surgically fused vertebral segments (Hilibrand et al. 1999). This indicates that stopping the movement of the spine following spinal fusion transfers the load onto adjacent spinal segments that leads to earlier disc degeneration-induced local inflammatory responses in the surrounding tissues, in particular the deep paraspinal muscles (Hilibrand et al. 1999; Burke et al. 2002; Hodges et al. 2014, 2015; James et al. 2016). Therefore, this research suggests that both altered intervertebral movement at the level of the CSMC problem and/or repeated microtrauma due to faulty vertebral motor control could both cause increased local inflammation around the area of a CSMC problem, that would be tender to the touch and could result in ongoing alterations in afferent input from slow-conducting (group III and IV) afferents in addition to changes in type I and type II deep muscle mechanoreceptive input.

Further support for this notion comes from a study that has shown that 2 weeks of spinal adjustments of CSMC problems decreased inflammatory mediators, indicating the potential for spinal adjustments to alter the inflammation at the area of CSMC problems (Teodorczyk-Injeyan et al. 2018). Other studies have suggested that adjustments of CSMC problems influence cortisol levels (Christian et al. 1988; Colombi and Testa 2019; Kovanur-Sampath et al. 2017; Plaza-Manzano et al. 2014). Recently, two reviews reported moderate-quality evidence that spinal adjustments alter cortisol and interleukin levels (Colombi and Testa 2019; Kovanur-Sampath et al. 2017). Cortisol, a glucocorticoid important for modulating the immune response, inhibits cytokines and inflammation (Buckingham et al. 1996; Chrousos 1995; Godbout and Glaser 2006; Herkenham and Kigar 2017; Mulla and Buckingham 1999). Interleukins are a type of cytokine that have pro-inflammatory and anti-inflammatory properties (Buckingham et al. 1996; Mulla and Buckingham 1999; Pearce et al. 2001; Silverman et al. 2005). Thus, the mechanism by which adjusting CSMC problems alters neuromuscular function may be by reducing pain and inflammation, by influencing cortisol and various cytokine levels (Colombi and Testa 2019; Kovanur-Sampath et al. 2017). However, the reductions in inflammation that have been shown to occur with spinal adjustments (Teodorczyk-Injeyan et al. 2018), may also be due to alterations in the processing of the prefrontal cortex (Lelic et al. 2016), which can directly activate the cholinergic anti-inflammatory pathway via the vagus nerve (Ahern et al. 2001; Moench and Wellman 2015; Thayer 2009) and because it inhibits proinflammatory effects of the sympathetic nervous system and the neuroendocrine hypothalamic–pituitary–adrenal (HPA) axis (Diorio et al. 1993). This will be discussed further in the second review.

In summary, multiple sensory receptors are known to influence motor control. The CNS integrates sensory information from multiple sensory modalities to build accurate maps, representations, or internal body and external world schemas of our internal and external environment (Tagliabue and McIntyre 2014; Harris et al. 2015). There is now a growing body of evidence that shows vertebral dysfunction and spinal adjustments can impact sensory receptors that could contribute to, or are known to influence, neuromuscular function (see Table 2) (Hodges et al. 2006, 2015; Meier et al. 2018; Burns et al. 2016; Chang et al. 2019; Reed and Pickar 2015; Pickar and Wheeler 2001; Pickar and Bolton 2012; Reed et al. 2015; Wirth et al. 2019). Good spinal function appears to be vital for the brain to accurately predict, monitor and execute the movement of the whole body (Bellan et al. 2017; Haavik and Murphy 2011; Holt et al. 2016a, b). When the spine is not moving properly, i.e. when CSMC problems are present, then the altered afferent input associated with these abnormal vertebral movement patterns appears to affect the ability of the brain to accurately update its internal maps of the body and the world around us, which can impact the way the brain controls the body’s movements and functions, that may lead to accidents and/or microtraumas and/or changes in other bodily functions. There is certainly plenty of evidence that supports the notion that spinal adjustments can influence or improve several aspects of motor control and neuromuscular performance (see Table 1) (Andrew et al. 2018; Christiansen et al. 2018; Daligadu et al. 2013; Haavik-Taylor and Murphy 2007b; Haavik et al. 2016a, b, 2017; 2018a, b; Haavik Taylor and Murphy 2008; Holt et al. 2019; Marshall and Murphy 2006; Niazi et al. 2015; Özyurt et al. 2019). This highlights the importance of good spinal function by adjusting CSMC problems to enable the brain to make accurate predictions, correct movement errors and move the whole body accurately. Altered sensory inputs from the abnormal spinal movement that occurs with a CSMC problem appear to impair the brain’s ability to accurately update the internal body schema within the brain and spinal cord. If the brain executes bodily movements while relying on an impaired or less than accurate internal body schema, it may result in injuries and/or microtraumas that can cause the development of symptoms. These maladaptive changes may cause central pain sensitisation and impair motor control of the spine, trunk, pelvic floor, head and limbs, which, when prolonged, can result in chronic pain disorders.

One might ask why the CNS does not adjust or ‘down-regulate’ input from these deep paraspinal muscles using input from more superficial muscles and/or other sensory modalities that could provide more accurate input and create more accurate brain maps that are not maladaptive. It is highly likely that the CNS does down-regulate the importance that it places on this “untrustworthy” deep muscle afferent input and this may indeed be part of the problem. We cannot visualise our deep paraspinal muscles, so we cannot use vision as a “back-up” system to know where these muscles are when muscle spindle feedback is inaccurate. In contrast, with our limbs, we can see them, so we have other ways of knowing if the positioning is faulty. This may in fact be part of the problem that perpetuates the presence of the CSMC problem. Recent work by Bornstein indicates that the proprioceptive system is important for far more than just the control and coordination of movement and posture (Bornstein et al. 2021). Faulty proprioception can lead to uncoordinated movement and altered muscle activation patterns, which in turn can lead to abnormal stressors on joints and that “such abnormal stressors on joints can in turn lead to abnormal mechanical signals in joint cells, affecting joint integrity and resulting in aberrant joint morphology” (Bornstein et al. 2021, p.85). This, therefore, provides a clear mechanism by which self-perpetuating central segmental motor control problem areas arise and are maintained.

The CNS may to some degree be able to use input from more superficial vertebral muscles to maintain spinal movement control, when the input from these deep paraspinal muscles is compromised (i.e. when there are CSMC problems). However, under certain conditions (for example stress), or due to certain personality traits, this may not be possible for everyone’s CNS to do. There might be some conditions, such as when a person is under a lot of psychological or physiological stress and/or during high attention-demanding situations, that such factors interfere with this process as these are known to influence various aspects of motor control (Hodges and Moseley 2003; Marras et al. 2000; Singaravelu et al. 2021; Mehta and Rhee 2017; Bertilsson 2019). It is also possible that the presence of fear and/or pain and even your personality might influence spinal motor control and its consequences (Moseley 2003; van Dieën et al. 2018, 2019). Singaravelu et al. (2021) have recently shown in an animal model that rats who were stressed repeatedly and then given a single dose of nerve growth factor (NGF) injected into a low back muscle caused sensitisation of their dorsal horn nerve cells from their deep lumbar multifidus muscles. This response was different than the response resulting from either stress alone or from the NGF injection alone (Singaravelu et al. 2021; Marras et al. 2000), clearly demonstrating that only when both stress and the NGF injection appeared at the same time did the sensitisation of their dorsal horn nerve cells from their deep lumbar multifidus muscles occur. Others have shown that psychological stress can alter trunk muscle activation during a lifting task (Marras et al. 2000), but in different ways for different people, possibly due to their personality types. Moseley et al. (2004) showed that adding stress to an attention-demanding task affected the postural activation of only the deep trunk muscles while not affecting the superficial trunk muscles or the deltoid muscle. This suggests that there may be certain conditions and certain personality types that predispose a person to a more negative influence on the motor control of the deep paraspinal muscles, which in turn for them may cause more trouble with their overall motor control compared with others. It may be that the CNS is, under certain conditions (such as less stress and less attention-demanding tasks) and for certain personality types (more positive, optimistic people) able to adjust or down-regulate the ‘faulty’ input from deep paraspinal muscles when they have a CSMC problem, thus resulting in less negative impact on their motor control. It is also possible that because of the higher density of muscle spindles in the deep paraspinal muscles, once activated by the rapid stretch from an adjustment, this bombardment of this mechanoreceptive input to the CNS has such a big impact, precisely because it has been missing and/or down-regulated due to the biomechanical problems or injury surrounding a CSMC problem segment.

## Summary and conclusion

This review has explored how vertebral column dysfunction, known as central segmental motor control (CSMC) problems, as well as how spinal adjustments and spinal manipulation alters neuromuscular function. Multiple studies have shown increases in force measures and prevention of fatigue building during strong repeated muscle contractions after spinal adjustments (see Table 1) (Christiansen et al. 2018; Holt et al. 2019; Haavik et al. 2018a, b; Niazi et al. 2015). Studies using TMS (Haavik et al. 2017; Haavik et al. 2018a, b; Haavik Taylor and Murphy 2008; Haavik-Taylor and Murphy 2007b), the H-reflex (Holt et al. 2019; Christiansen et al. 2018; Niazi et al. 2015), F waves (Haavik Taylor and Murphy 2008; Haavik-Taylor and Murphy 2007b), MRCPs (Haavik et al. 2017), V waves (Holt et al. 2019; Christiansen et al. 2018; Niazi et al. 2015), surface EMG (both single electrodes and high density (HD) electrodes) (Haavik-Taylor and Murphy 2007b; Haavik Taylor and Murphy 2008; Haavik et al. 2017, 2018a, b), and intramuscular EMG (Haavik et al. 2018a, b) indicate spinal adjustments predominantly alter supraspinal excitability (see Table 1). The evidence showing how spinal adjustments alter supraspinal multimodal integration and motor control will be covered in the second invited review. However, this current review has summarised the current contemporary model that provides a biologically plausible explanation for how the vertebral column’s central neural motor control can dysfunction, leading to a self-perpetuating central segmental motor control problem (see Figs. 1, 2).

According to the literature, physical injury, pain, inflammation and acute or chronic physiological or psychological stress all appear capable of altering vertebral column (in particular head on neck) proprioception and thus can influence vertebral column motor control by altering signalling from the deep paraspinal muscles or the central processing of such input (Hellström et al. 2005; Passatore and Roatta 2006; Brown et al. 2011; Butler and Moseley 2003; Hodges et al. 2006, 2009, 2014, 2015; James et al. 2016; Le Pera et al. 2001; Thunberg et al. 2001). There is evidence from animal models that changes in vertebral motion segment movement are, for the most part, signalled to the CNS via deep paraspinal muscle afferents (type I and II) (see Table 2) (Bolton and Holland 1996, 1998; Bolton 2000). However, there is also evidence that afferent input from a CSMC problem involves group III and IV afferents signalling local inflammation from the tissues surrounding the CSMC problem (see Table 2) (Burke et al. 2002; Hilibrand et al. 1999), possibly due to microtraumas occurring at that segment due to the poor central segmental motor control. It is known that whiplash-induced injuries to the cervical spine are capable of changing cervical paraspinal afferent input that can permanently change cervical reflex connections to the visual and vestibular systems and result in subsequent secondary disturbances, such as dizziness and visual disturbances (Solarino et al. 2009). However, as this review has discussed, it is not only cervical reflex connections that can change due to altered afferent input from the deep paraspinal muscles, as other studies have shown, this can also change the way various parts of the CNS integrates this afferent information with memories and/or the current movement goal, and that it can impact various anticipatory feedforward and/or feedback postural control mechanisms, and thus may also impact the fine-tuning of movements or even the efference copies and/or the actual movement commands sent to the various muscles (see Fig. 1) (Marshall and Murphy 2006; Hodges and Moseley 2003; Meier et al. 2018; MacDonald et al. 2006).

This review has also discussed the contemporary biologically plausible understanding of how spinal adjustments, i.e. the high-velocity, low-amplitude thrust directed at a CSMC problem, impacts human neuromuscular function. Evidence from animal studies, where the high-velocity, low amplitude thrusts were directed at a pre-determined level, i.e. spinal manipulation, have been discussed. These studies indicate that spinal manipulation activates the deep muscle afferents from paravertebral tissues, particularly activating the muscle spindles and potentially Golgi tendon organs (see Table 2) (Reed et al. 2017a, b; Kent 1996; Henderson 2012; Cao and Pickar 2014; Taylor et al. 2010; Haavik and Murphy 2012; Pickar and Bolton 2012; Pickar and Wheeler 2001). The evidence that suggests that such changes alter supraspinal multimodal integration centres will be discussed in the second invited review. Throughout this review, the many gaps in the literature have also been identified, along with suggestions for future studies.

As we have highlighted, there are multiple gaps in the literature regarding the exact mechanisms by which CSMC problems and spinal adjustments and manipulations alter afferent input to the CNS, how the CNS integrates this information and changes motor control and neuromuscular function. As noted, there appears to be different central neurophysiological effects from an adjustment, i.e. an HVLA thrust directed at a CSMC problem, compared with a manipulation of a vertebral segment with no evidence of (‘Association of Chiropractic Colleges Research Agenda Conference 2021 Abstracts of Proceedings’ 2021). This should be further explored in future studies, to better understand what impact this may have on clinical outcomes. Some studies have not specified the reasons for applying HVLA thrusts in their manuscripts, complicating this issue. There are also limitations in human studies where spinal adjustments or manipulations are applied due to the inherent difficulty in blinding the subjects and health care providers. These limitations make it very difficult to know what components of the therapeutic intervention induce the changes observed in these studies. This must be kept in mind when interpreting the results from any spinal adjustment or manipulation intervention study.

Future studies should also explore whether spinal adjustments could prevent or treat the maladaptive plastic changes that occur within the deep intervertebral paraspinal muscles following a disc injury. Finally, we still lack a clear idea about which exact sensory organs are responsible for maintaining a CSMC problem and which are involved in the mechanisms of spinal adjustments and manipulations. We do not yet know for sure whether only the deep muscle mechanoreceptors are involved or whether mechanoreceptors in surrounding tissues also play a role. We suspect with future experiments that additional mechanoreceptors in other tissues surrounding the CSMC problem will be identified as capable of impacting central CNS processing, integration and thus neuromuscular function. Mechanoreceptors in fascia have recently been shown to play a much larger role in force transmission to neighbouring structures within a limb (between synergists) and along muscle-fascia chains, such as between legs and the trunk [for review see (Wilke et al. 2018)]. The posterior myofascial plane extends from the occiput down to the toes and consists of the lumbar fascia/erector spinae muscles, the sacrotuberous ligament, the hamstring muscles, the gastrocnemius muscle, the Achilles tendon and the plantar aponeurosis (for review see (Wilke et al. 2018). This in-series arrangement of the components, suggests direct continuity between head and toes and may eventually explain why lower limb excitability changes are so much larger than upper limb neuromuscular changes following spinal adjustments. And recent research is already highlighting that the proprioceptive system seems to be important for far more than just the control and coordination of movement and posture (Bornstein et al. 2021). It is now implicated in the regulation of a wide range of developmental and physiological processes, meaning the wider implications of CSMC problems beyond movement control of the spine are yet to be discovered.

Thus, there is a long way to go to fully understand the mechanisms by which altered afferent input from the vertebral column impacts the human neuromuscular system, let alone the clinical implications of such changes. However, it is clear from this review that physical injury, pain, inflammation and acute or chronic physiological or psychological stress can alter signalling from the deep paraspinal muscles to the CNS (Hellström et al. 2005; Passatore and Roatta 2006; Brown et al. 2011; Butler and Moseley 2003; Hodges et al. 2006, 2009, 2014, 2015; James et al. 2016; Le Pera et al. 2001; Thunberg et al. 2001). There is supporting evidence from animal models that changes in vertebral motion segment movement is, for the most part, signalled to the CNS via deep paraspinal muscle afferents (type I and II) (Bolton and Holland 1996, 1998; Bolton 2000). However, there is also evidence that afferent input from a CSMC problem involves group III and IV afferents signalling local inflammation from the tissues surrounding the CSMC problem (Burke et al. 2002; Hilibrand et al. 1999), possibly due to microtraumas occurring at that segment due to the poor central motor control of that segment. What is clear from this review is that any of the following conditions, such as physical injury, pain, inflammation, acute or chronic physiological or psychological stress, can alter vertebral column afferent input that may also, for certain vulnerable people, impede their CNS’s ability to accurately sense what is occurring at that part of the spine, which in turn can alter the way it controls that part of the vertebral column, in other words, can result in vertebral segmental microtraumas and self-perpetuating central segmental motor control problems that may over time result in recurrent spinal ache, pain or tension and the development of chronic vertebral column pain syndromes. Thus, any of these conditions, including physical injury, psychological stress, pain or inflammation, is thought to be able to initiate a central segmental motor control problem.

Furthermore, it is clear from this review that the high-velocity, low-amplitude thrusts directed at the spine are capable of activating the deep muscle afferents from paravertebral tissues, particularly activating the muscle spindles and potentially Golgi tendon organs (Reed et al. 2017a, b; Kent 1996; Henderson 2012; Cao and Pickar 2014; Taylor et al. 2010; Haavik and Murphy 2012; Pickar and Bolton 2012; Pickar and Wheeler 2001) It is also clear from this review that spinal adjustments of CSMC problems do impact motor control in a variety of ways, but in particular, by increasing muscle force measures and prevention of fatigue building during strong repeated muscle contractions (Christiansen et al. 2018; Holt et al. 2019; Haavik et al. 2018a, b; Niazi et al. 2015). Other studies strongly suggest that these changes in neuromuscular function most likely occur due to changes in supraspinal excitability (Haavik et al. 2017, 2018a, b; Haavik Taylor and Murphy 2008; Haavik-Taylor and Murphy 2007b; Holt et al. 2019; Christiansen et al. 2018; Niazi et al. 2015), which will be discussed in greater detail in the second invited review. Finally, this review has presented and discussed the current contemporary model of the CSMC problem and the mechanisms of spinal adjustments that provide a biologically plausible explanation for how the vertebral column’s central neural motor control can end up dysfunctional, leading to a self-perpetuating central segmental motor control problem, and how HVLA spinal adjustments can improve neuromuscular function.

## Abbreviations

AMCT: Activator methods chiropractic technique
ASMT: Activator-assisted spinal manipulative therapy
APB: Abductor pollicis brevis
CSMC: Central segmental motor control
CNS: Central nervous system
CSP: Cortical silent period
EIP: Extensor indicis proprius
EMG: Electromyography
EBP: Early bereitschaftpotential
FFA: Feed-forward activation
GTO: Golgi tendon organ
HD: High density
HVLA: High-velocity, low-amplitude
IVF: Intervertebral foramina
LBP: Late bereitschaftpotential
M1: Primary motor cortex
MEP: Motor-evoked potential
MRCPs: Movement-related cortical potentials
MS: Muscle spindle
MVC: Maximum voluntary contractions
PN: Peak negativity
PSF: Peristimulus frequencygrams
PSTH: Peristimulus time histogram
SA: Spinal adjustment
SCSP: Subclinical spinal pain
SCNP: Subclinical neck pain
sEMG: Surface electromyography
SICI: Short-interval intracortical inhibition
SICF: Short-interval intracortical facilitation
SM: Spinal manipulation
SMUs: Single motor units
SNAG: Sustained natural apophyseal glides
SR: Stimulus response
TA: Tibialis anterior
TMS: Transcranial magnetic stimulation
TNF: Tumour necrosis factor
UMN: Upper motor neuron

## References

[CR1] Abelew TA, Miller MD, Cope TC, Nichols TR (2000). Local loss of proprioception results in disruption of interjoint coordination during locomotion in the cat. J Neurophysiol.

[CR2] Adams J, Peng W, Cramer H, Sundberg T, Moore C, Amorin-Woods L, Sibbritt D, Lauche R (2017). The prevalence, patterns, and predictors of chiropractic use among us adults: results from the 2012 national health interview survey. Spine.

[CR3] Ahern GL, Sollers JJ, Lane RD, Labiner DM, Herring AM, Weinand ME, Hutzler R, Thayer JF (2001). Heart rate and heart rate variability changes in the intracarotid sodium amobarbital test. Epilepsia.

[CR4] Akay T, Tourtellotte WG, Arber S, Jessell TM (2014). Degradation of mouse locomotor pattern in the absence of proprioceptive sensory feedback. Proc Natl Acad Sci.

[CR5] Alaranta H, Tallroth K, Soukka A, Heliovaara M (1993). Fat content of lumbar extensor muscles and low back disability: a radiographic and clinical comparison. J Spinal Disord.

[CR6] Alcantara J, Alcantara JD, Alcantara J, Anrig C, Plaugher G (2013). Spinal subluxation. Pediatric chiropractic.

[CR7] Allison GT, Morris SL, Lay B (2008). Feedforward responses of transversus abdominis are directionally specific and act asymmetrically: implications for core stability theories. J Orthopaedic Sports Phys Therapy.

[CR8] Amonoo-Kuofi HS (1983). The density of muscle spindles in the medial, intermediate and lateral columns of human intrinsic postvertebral muscles. J Anat.

[CR9] Anderst WJ, Gale T, LeVasseur C, Raj S, Gongaware K, Schneider M (2018) Intervertebral kinematics of the cervical spine before, during and after high velocity low amplitude manipulation. Spine J 10.1016/j.spinee.2018.07.026 30142458

[CR10] Andrew D, Yielder P, Haavik H, Murphy B (2018). The effects of subclinical neck pain on sensorimotor integration following a complex motor pursuit task. Exp Brain Res.

[CR11] Association of Chiropractic Colleges (1996) The association of chiropractic colleges position paper # 1. July 1996. ', *ICA Rev*, November/December

[CR12] Association of Chiropractic Colleges Research Agenda Conference 2021 Abstracts of Proceedings. 2021. J Chiropractic Educ 35: 81-94 10.7899/JCE-20-25 PMC7958663 33825893

[CR13] Awiszus F, Feistner H (1994). Quantification of D-and I-wave effects evoked by transcranial magnetic brain stimulation on the tibialis anterior motoneuron pool in man. Exp Brain Res.

[CR14] Baarbe JK, Holmes MW, Murphy HE, Haavik H, Murphy BA (2016). Influence of subclinical neck pain on the ability to perform a mental rotation task: a 4-week longitudinal study with a healthy control group comparison. J Manipulative Physiol Ther.

[CR15] Baarbé JK, Yielder P, Haavik H, Holmes MWR, Murphy BAnn (2018). Subclinical recurrent neck pain and its treatment impacts motor training-induced plasticity of the cerebellum and motor cortex. PLoS ONE.

[CR16] Barker AT, Freeston IL, Jalinous R, Merton PA (1985). Magnetic stimulation of the human brain. J Physiol.

[CR17] Barker AT, Garnham CW, Freeston IL (1991). Magnetic nerve stimulation: the effect of waveform on efficiency, determination of neural membrane time constants and the measurement of stimulator output. Electroencephalography Clin Neurophysiol Suppl.

[CR18] Bawa P, Lemon R (1993). Recruitment of motor units in response to transcranial magnetic stimulation in man. J Physiol (london).

[CR19] Beinert K, Keller M, Taube W (2015). Neck muscle vibration can improve sensorimotor function in patients with neck pain. Spine J.

[CR20] Bellan V, Wallwork SB, Gallace A, Spence C, Lorimer Moseley G (2017). Integrating self-localization proprioception, pain, and performance. J Dance Med Sci: off Publ Int Assoc Dance Med Sci.

[CR21] Benjamin M (2009). The fascia of the limbs and back–a review. J Anat.

[CR22] Bertilsson J (2019) Human motor control, autonomic and decision processes under physical and psychological stress. Instinctive, reflexive and adaptive aspects. Lund University

[CR23] Bestmann S, Ruff CC, Blankenburg F, Weiskopf N, Driver J, Rothwell JC (2008). Mapping causal interregional influences with concurrent TMS–fMRI. Exp Brain Res.

[CR24] Blanke O, Slater M, Serino A (2015). Behavioral, neural, and computational principles of bodily self-consciousness. Neuron.

[CR25] Blecher R, Krief S, Galili T, Biton IE, Stern T, Assaraf E, Levanon D, Appel E, Anekstein Y, Agar G (2017). The proprioceptive system masterminds spinal alignment: insight into the mechanism of scoliosis. Dev Cell.

[CR26] Blum KP, D’Incamps BL, Zytnicki D, Ting LH (2017). Force encoding in muscle spindles during stretch of passive muscle. PLOS Comput Biol.

[CR27] Boal RW, Gillette RG (2004). Central neuronal plasticity, low back pain and spinal manipulative therapy. J Manipulative Physiol Ther.

[CR28] Bogduk N, Marsland A (1988). The cervical zygapophyseal joints as a source of neck pain. Spine.

[CR29] Bolton PS (2000). Reflex effects of vertebral subluxations: the peripheral nervous system. An update. J Manipulative Physiol Ther.

[CR30] Bolton PS, Holland CT (1996). Afferent signaling of vertebral displacement in the neck of the cat. Soc Neurosci Abstr.

[CR31] Bolton PS, Holland CT (1998). An in vivo method for studying afferent fibre activity from cervical paravertebral tissue during vertebral motion in anaesthetised cats. J Neurosci Methods.

[CR32] Borenstein DG, O'Mara JW, Boden SD, Lauerman WC, Jacobson A, Platenberg C, Schellinger D, Wiesel SW (2001). The value of magnetic resonance imaging of the lumbar spine to predict low-back pain in asymptomatic subjects: a seven-year follow-up study. JBJS.

[CR33] Bornstein B, Konstantin N, Alessandro C, Tresch MC, Zelzer E (2021) More than movement: The proprioceptive system as a new regulator of musculoskeletal biology. Curr Opinion Physiol

[CR34] Bosco G, Poppele RE (2001). Proprioception from a spinocerebellar perspective. Physiol Rev.

[CR35] Botelho MB, Andrade BB (2012). Effect of cervical spine manipulative therapy on judo athletes' grip strength. J Manipulative Physiol Ther.

[CR36] Boyd-Clark LC, Briggs CA, Galea MP (2002). Muscle spindle distribution, morphology, and density in *Longus colli* and multifidus muscles of the cervical spine. Spine.

[CR37] Bracht MA, Coan ACB, Yahya A, Dos Santos MJ (2018). Effects of cervical manipulation on pain, grip force control, and upper extremity muscle activity: a randomized controlled trial. J Man Manip Ther.

[CR38] Brinkworth RSA, Tuncer M, Tucker KJ, Jaberzadeh S, Türker KS (2007). Standardization of H-reflex analyses. J Neurosci Methods.

[CR39] Brown SH, Gregory DE, Carr JA, Ward SR, Masuda K, Lieber RL (2011). ISSLS prize winner: adaptations to the multifidus muscle in response to experimentally induced intervertebral disc degeneration. Spine.

[CR40] Brumagne S, Cordo P, Lysens R, Verschueren S, Swinnen S (2000). The role of paraspinal muscle spindles in lumbosacral position sense in individuals with and without low back pain. Spine.

[CR41] Brumagne S, Diers M, Danneels GL, Moseley L, Hodges PW (2019). Neuroplasticity of sensorimotor control in low back pain. J Orthopaedic Sports Phys Therapy.

[CR42] Buckingham JC, Loxley HD, Christian HC, Philip JG (1996). Activation of the HPA axis by immune insults: roles and interactions of cytokines, eicosanoids, and glucocorticoids. Pharmacol Biochem Behav.

[CR43] Burgess PR, Wei JY, Clark FJ, Simon J (1982). Signaling of kinesthetic information by peripheral sensory receptors. Annu Rev Neurosci.

[CR44] Burke DAVID, Hagbarth KARL-ERIK, Löfstedt L, Wallin BG (1976). The responses of human muscle spindle endings to vibration of non-contracting muscles. J Physiol.

[CR45] Burke JG, Watson RWG, McCormack DRWG, Dowling FE, Walsh MG, Fitzpatrick JM (2002). Intervertebral discs which cause low back pain secrete high levels of proinflammatory mediators. J Bone Joint Surg British.

[CR46] Burns E, Chipchase LS, Schabrun SM (2016). Primary sensory and motor cortex function in response to acute muscle pain: a systematic review and meta-analysis. Eur J Pain.

[CR47] Butler D, Lorimer Moseley G (2003). Explain pain.

[CR48] Cadwell J (1990) Principles of magnetoelectric stimulation. Magn Stimulation Clin Neurophysiol 13–32

[CR49] Cantello R, Gianelli M, Civardi C, Mutani R (1992). Magnetic brain stimulation: the silent period after the motor evoked potential. Neurology.

[CR50] Cao D-Y, Pickar JG (2014). Effect of spinal manipulation on the development of history-dependent responsiveness of lumbar paraspinal muscle spindles in the cat. J Can Chiropr Assoc.

[CR51] Cao DY, Reed WR, Long CR, Kawchuk GN, Pickar JG (2013). Effects of thrust amplitude and duration of high-velocity, low-amplitude spinal manipulation on lumbar muscle spindle responses to vertebral position and movement. J Manipulative Physiol Ther.

[CR52] Cavallari P, Bolzoni F, Bruttini C, Esposti R (2016). The organization and control of intra-limb anticipatory postural adjustments and their role in movement performance. Front Hum Neurosci.

[CR53] Cavanaugh JM, Ying Lu, Chen C, Kallakuri S (2006). Pain generation in lumbar and cervical facet joints. JBJS.

[CR54] Chang W-J, Buscemi V, Liston MB, McAuley JH, Hodges PW, Schabrun SM (2019). Sensorimotor cortical activity in acute low back pain: a cross-sectional study. J Pain.

[CR55] Chen R, Garg R (2000). Facilitatory I wave interaction in proximal arm and lower limb muscle representations of the human motor cortex. J Neurophysiol.

[CR56] Chen WG, Schloesser D, Arensdorf AM, Simmons JM, Cui C, Valentino R, Gnadt JW, Nielsen L, Hillaire-Clarke CS, Spruance V, Horowitz TS, Vallejo YF, Langevin HM (2021). The emerging science of interoception: sensing, integrating, interpreting, and regulating signals within the self. Trends Neurosci.

[CR57] Chilibeck PD, Cornish SM, Schulte Al, Jantz N, Magnus CRA, Schwanbeck S, Juurlink BHJ (2011). The effect of spinal manipulation on imbalances in leg strength. J Can Chiropr Assoc.

[CR58] Cholewicki J, Silfies SP, Shah RA, Greene HS, Reeves NP, Alvi K, Goldberg B (2005). Delayed trunk muscle reflex responses increase the risk of low back injuries. Spine.

[CR59] Christian GF, Stanton GJ, Sissons D, How HY, Jamison J, Alder B, Fullerton M, Funder JW (1988). Immunoreactive ACTH, beta-endorphin, and cortisol levels in plasma following spinal manipulative therapy. Spine.

[CR60] Christiansen TL, Niazi IK, Holt K, Nedergaard RW, Duehr J, Allen K, Marshall P, Türker KS, Hartvigsen J, Haavik H (2018). The effects of a single session of spinal manipulation on strength and cortical drive in athletes. Eur J Appl Physiol.

[CR61] Christopher Kent DC (1996). Models of vertebral subluxation: a review. J Vertebral Subluxation Res.

[CR62] Chrousos GP (1995). The hypothalamic–pituitary–adrenal axis and immune-mediated inflammation. N Engl J Med.

[CR63] Clark BC, Goss DA, Walkowski S, Hoffman RL, Ross A, Thomas JS (2011). Neurophysiologic effects of spinal manipulation in patients with chronic low back pain. BMC Musculoskelet Disord.

[CR64] Cleland J, Selleck B, Stowell T, Browne L, Alberini S, StCyr H, Caron T (2004). Short-term effects of thoracic manipulation on lower trapezius muscle strength. J Manual Manipulative Ther.

[CR65] Colloca CJ, Keller TS, Gunzburg R (2003). Neuromechanical characterization of in vivo lumbar spinal manipulation. Part II. Neurophysiological response. J Manipulative Physiol Ther.

[CR66] Colloca CJ, Keller TS, Harrison DE, Moore RJ, Gunzburg R, Harrison DD (2006). Spinal manipulation force and duration affect vertebral movement and neuromuscular responses. Clin Biomech (bristol, Avon).

[CR67] Colloca CJ, Keller TS, Moore RJ, Gunzburg R, Harrison DE (2008). Effects of disc degeneration on neurophysiological responses during dorsoventral mechanical excitation of the ovine lumbar spine. J Electromyogr Kinesiol.

[CR68] Colloca CJ, Gunzburg R, Freeman BJ, Szpalski M, Afifi M, Moore RJ (2012). Biomechanical quantification of pathologic manipulable spinal lesions: an in vivo ovine model of spondylolysis and intervertebral disc degeneration. J Manipulative Physiol Ther.

[CR69] Colombi A, Testa M (2019). The effects induced by spinal manipulative therapy on the immune and endocrine systems. Medicina.

[CR70] Cooley JR, Walker BF, Ardakani EM, Kjaer P, Jensen TS, Hebert JJ (2018). Relationships between paraspinal muscle morphology and neurocompressive conditions of the lumbar spine: a systematic review with meta-analysis. BMC Musculoskelet Disord.

[CR71] Cooper S, Daniel PM (1963). Muscle sindles in man; their morphology in the lumbricals and the deep muscles of the neck. Brain.

[CR72] Cooperstein R, Gleberzon B (2004). Technique systems in chiropractic.

[CR73] Cooperstein R, Haneline M, Young M (2010) The reliability of cervical motion palpation using continuous analysis and confidence ratings. In Association of Chiropractic Colleges Educational Conference—Research Agenda Conference (ACC-RAC). Las Vegas, Nevada: The Journal of Chiropractic Education Vol 24 (1) p88.

[CR74] Cooperstein R, Young M, Haneline M (2013). Interexaminer reliability of cervical motion palpation using continuous measures and rater confidence levels. J Can Chiropr Assoc.

[CR75] Cordo PJ, Flores-Vieira C, Verschueren SM, Inglis JT, Gurfinkel V (2002). Position sensitivity of human muscle spindles: single afferent and population representations. J Neurophysiol.

[CR76] Coulter ID, Shekelle PG (2005). Chiropractic in North America: a descriptive analysis. J Manipulative Physiol Ther.

[CR77] Craig AD (2002). How do you feel? Interoception: the sense of the physiological condition of the body. Nat Rev Neurosci.

[CR78] Craig AD (2003). Interoception: the sense of the physiological condition of the body. Curr Opin Neurobiol.

[CR79] Craig AD, Craig AD (2009) How do you feel—now? The anterior insula and human awareness. Nat Rev Neurosci 10 10.1038/nrn2555 19096369

[CR80] Cramer G, Budgell B, Henderson C, Khalsa P, Pickar J (2006). Basic science research related to chiropractic spinal adjusting: the state of the art and recommendations revisited. J Manipulative Physiol Ther.

[CR81] Critchley HD, Garfinkel SN (2017). Interoception and emotion. Curr Opin Psychol.

[CR82] Critchley HD, Harrison NA (2013). Visceral influences on brain and behavior. Neuron.

[CR83] Daligadu J, Haavik H, Yielder P, Baarbe J, Murphy B (2013). Alterations in cortical and cerebellar motor processing in sub-clinical neck pain patients following spinal manipulation. J Manipulative Physiol Ther.

[CR84] Devanne H, Lavoie BA, Capaday C (1997). Input-output properties and gain changes in the human corticospinal pathway. Exp Brain Res.

[CR85] Diorio D, Viau V, Meaney MJ (1993). The role of the medial prefrontal cortex (cingulate gyrus) in the regulation of hypothalamic-pituitary-adrenal responses to stress. J Neurosci.

[CR86] Du Rose A, Breen A (2016) Relationships between paraspinal muscle activity and lumbar inter-vertebral range of motion. In Healthcare, 4. Multidisciplinary Digital Publishing Institute 10.3390/healthcare4010004 PMC4934538 27417592

[CR87] Duarte FCK, Kolberg C, Riffel APK, Souza JA, Belló-Klein A, Partata WA (2019). Spinal manipulation therapy improves tactile allodynia and peripheral nerve functionality and modulates blood oxidative stress markers in rats exposed to knee-joint immobilization. J Manipulative Physiol Ther.

[CR88] Dunning J, Rushton A (2009). The effects of cervical high-velocity low-amplitude thrust manipulation on resting electromyographic activity of the biceps brachii muscle. Man Ther.

[CR89] Ebrall P, Draper B, Repka A (2008). Towards a 21 century paradigm of chiropractic: stage 1, redesigning clinical learning. J Chiropr Educ.

[CR90] Ekblom N, Maria M (2010). Improvements in dynamic plantar flexor strength after resistance training are associated with increased voluntary activation and V-to-M ratio. J Appl Physiol.

[CR91] Elliott JM, Mark Courtney D, Rademaker A, Pinto D, Sterling MM, Parrish TB (2015). The rapid and progressive degeneration of the cervical multifidus in whiplash: a MRI study of fatty infiltration. Spine.

[CR92] Evans DW (2002). Mechanisms and effects of spinal high-velocity, low-amplitude thrust manipulation: previous theories. J Manipulative Physiol Ther.

[CR93] Farid B, Yielder P, Holmes M, Haavik H, Murphy BA (2018). Association of subclinical neck pain with altered multisensory integration at baseline and 4-week follow-up relative to asymptomatic controls. J Manipulative Physiol Ther.

[CR94] Fernandez-Carnero J, Fernandez-de-las-Penas C, Cleland JA (2008). Immediate hypoalgesic and motor effects after a single cervical spine manipulation in subjects with lateral epicondylalgia. J Manipulative Physiol Ther.

[CR95] Fiehler K, Ullsperger M, Von Yves D, Cramon.  (2004). Neural correlates of error detection and error correction: is there a common neuroanatomical substrate?. Eur J Neurosci.

[CR96] Ford DM, Bagnall KM, Clements CA, McFadden KD (1988). Muscle spindles in the paraspinal musculature of patients with adolescent idiopathic scoliosis. Spine.

[CR97] Fortin M, Lazáry À, Varga PP, McCall I, Battié MC (2016). Paraspinal muscle asymmetry and fat infiltration in patients with symptomatic disc herniation. Eur Spine J.

[CR98] Freeman MAR, Wyke B (1966). Articular contributions to limb muscle reflexes. The effects of partial neurectomy of the knee-joint on postural reflexes. Br J Surg.

[CR99] Fritz C, Braune HJ, Pylatiuk C, Pohl M (1997). Silent period following transcranial magnetic stimulation: a study of intra- and inter-examiner reliability. Electroencephalogr Clin Neurophysiol.

[CR100] Fujiwara K, Toyama H, Kunita K (2003). Anticipatory activation of postural muscles associated with bilateral arm flexion in subjects with different quiet standing positions. Gait Posture.

[CR101] Galindez-Ibarbengoetxea X, Setuain I, Andersen LL, Ramirez-Velez R, Gonzalez-Izal M, Jauregi A, Izquierdo M (2017). Effects of cervical high-velocity low-amplitude techniques on range of motion, strength performance, and cardiovascular outcomes: a review. J Altern Complement Med.

[CR102] García-Pérez-Juana D, Fernández-de-las-Peñas C, Arias-Buría JL, Cleland JA, Plaza-Manzano G, Ortega-Santiago R (2018). Changes in cervicocephalic kinesthetic sensibility, widespread pressure pain sensitivity, and neck pain after cervical thrust manipulation in patients with chronic mechanical neck pain: a randomized clinical trial. J Manipulative Physiol Ther.

[CR103] Gatterman ML (1995). Foundations of chiropractic: subluxation.

[CR104] Gatterman MI, Hansen DT (1994). Development of chiropractic nomenclature through consensus. J Manipulative Physiol Ther.

[CR105] Geddes LA, Bourland JD (1983) Fundamentals of Eddy-Current (Magnetic) stimulation. In: S. Chokroverty (ed.), Magnetic stimulation in clinical neurophysiology

[CR106] Ghez C, Gordon J, Ghilardi MF (1995). Impairments of reaching movements in patients without proprioception. II. Effects of visual information on accuracy. J Neurophysiol.

[CR107] Gibson J, McCarron T (2004). Feedforward muscle activity: an investigation into the onset and activity of internal oblique during two functional reaching tasks. J Bodyw Mov Ther.

[CR108] Gilman S (2002). Joint position sense and vibration sense: anatomical organisation and assessment. J Neurol Neurosurg Psychiatry.

[CR109] Godbout JP, Glaser R (2006). Stress-induced immune dysregulation: implications for wound healing, infectious disease and cancer. J Neuroimmune Pharmacol.

[CR110] Gong W (2013). Effects of cervical joint manipulation on joint position sense of normal adults. J Phys Ther Sci.

[CR111] Gong W-Y, Abdelhamid RE, Carvalho CS, Sluka KA (2016). Resident macrophages in muscle contribute to development of hyperalgesia in a mouse model of noninflammatory muscle pain. J Pain.

[CR112] Gordon JAMES, Ghilardi MF, Ghez C (1995). Impairments of reaching movements in patients without proprioception. I. Spatial errors. J Neurophysiol.

[CR113] Gorrell LM, Beath K, Engel RM (2016). Manual and instrument applied cervical manipulation for mechanical neck pain: a randomized controlled trial. J Manipulative Physiol Ther.

[CR114] Gregory NS, Brito RG, Fusaro MC, Sluka KA (2016). ASIC3 is required for development of fatigue-induced hyperalgesia. Mol Neurobiol.

[CR115] Grigg P, Schaible H-G, Schmidt RF (1986). Mechanical sensitivity of group III and IV afferents from posterior articular nerve in normal and inflamed cat knee. J Neurophysiol.

[CR116] Grindstaff TL, Hertel J, Beazell JR, Magrum EM, Ingersoll CD (2009). Effects of lumbopelvic joint manipulation on quadriceps activation and strength in healthy individuals. Man Ther.

[CR117] Grostic JD (1988). Dentate ligament-cord distortion hypothesis. Chiropr Res J.

[CR118] Gyer G, Michael J, Inklebarger J, Tedla JS (2019). Spinal manipulation therapy: is it all about the brain? A current review of the neurophysiological effects of manipulation. J Integr Med.

[CR119] Haavik H (2014). The reality check: A quest to understand chiropractic from the inside out.

[CR120] Haavik H, Murphy B (2011). Subclinical neck pain and the effects of cervical manipulation on elbow joint position sense. J Manipulative Physiol Ther.

[CR121] Haavik H, Murphy B (2012). The role of spinal manipulation in addressing disordered sensorimotor integration and altered motor control. J Electromyogr Kinesiol.

[CR122] Haavik H, Niazi IK, Duehr J, Kinget M, Uginicius P, Sebik O, Yilmaz G, Navid MS, Türker KS (2016a) Chiropractic alters TMS induced I-wave excitability and cortical silent period duration. In International MotoNeuron Conference. Istanbul: International MotoNeuron Society

[CR123] Haavik H, Murphy B, Kruger J (2016). Effect of spinal manipulation on pelvic floor functional changes in pregnant and nonpregnant women: a preliminary study. J Manipulative Physiol Ther.

[CR124] Haavik H, Niazi I, Jochumsen M, Sherwin D, Flavel S, Türker K (2017). Impact of spinal manipulation on cortical drive to upper and lower limb muscles. Brain Sci.

[CR125] Haavik H, Niazi IK, Jochumsen M, Uginčius P, Sebik O, Yılmaz G, Navid MS, Özyurt MG, Türker KS (2018). Chiropractic spinal manipulation alters TMS induced I-wave excitability and shortens the cortical silent period. J Electromyogr Kinesiol.

[CR126] Haavik H, Özyurt MG, Niazi IK, Holt K, Nedergaard RW, Yilmaz G, Türker KS (2018). Chiropractic manipulation increases maximal bite force in healthy individuals. Brain Sci.

[CR127] Haavik-Taylor H, Murphy B (2007). Cervical spine manipulation alters sensorimotor integration: a somatosensory evoked potential study. Clin Neurophysiol.

[CR128] Haavik-Taylor H, Murphy B (2007). Transient modulation of intracortical inhibition following spinal manipulation. Chiropractic J Australia.

[CR129] Haavik Taylor H, Murphy B (2008). Altered sensorimotor integration with cervical spine manipulation. J Manipulative Physiol Ther.

[CR130] Haavik Taylor H, Murphy B (2010). Altered central integration of dual somatosensory input following cervical spine manipulation. J Manipulative Physiol Ther.

[CR131] Haavik Taylor H, Murphy B (2010). The effects of spinal manipulation on central integration of dual somatosensory input observed following motor training: a crossover study. J Manipulative Physiol Ther.

[CR132] Haavik Taylor H, Holt K, Murphy B (2010). Exploring the neuromodulatory effects of the vertebral subluxation and chiropractic care. Chiropractic J Australia.

[CR133] Hanajima R, Tsutsumi R, Shirota Y, Shimizu T, Tanaka N, Ugawa Y (2016) Cerebellar dysfunction in essential tremor. Movement Disorders 10.1002/mds.26629 27062434

[CR134] Harris LR, Carnevale MJ, D’Amour S, Fraser LE, Harrar V, Hoover AEN, Mander C, Pritchett LM (2015) How our body influences our perception of the world. Front Psychol 6 10.3389/fpsyg.2015.00819 PMC4464078 26124739

[CR135] Hart J (2016). Analysis and Adjustment of Vertebral Subluxation as a Separate and Distinct Identity for the Chiropractic Profession: A Commentary. J Chiropr Humanit.

[CR136] Hellström F, Roatta S, Thunberg J, Passatore M, Djupsjöbacka M (2005). Responses of muscle spindles in feline dorsal neck muscles to electrical stimulation of the cervical sympathetic nerve. Exp Brain Res.

[CR137] Henderson CN (2012). The basis for spinal manipulation: chiropractic perspective of indications and theory. J Electromyogr Kinesiol.

[CR138] Hennenhoefer K, Schmidt D (2019). Toward a theory of the mechanism of high-velocity, low-amplitude technique: a literature review. J Am Osteopath Assoc.

[CR139] Herkenham M, Kigar SL (2017). Contributions of the adaptive immune system to mood regulation: mechanisms and pathways of neuroimmune interactions. Prog Neuropsychopharmacol Biol Psychiatry.

[CR140] Herzog W, Lawrence DJ, Cassidy JD, McGregor M, Meeker WC, Vernon HT (1996). Mechanical, physiologic, and neuromuscular considerations of chiropractic treatment. Advances in chiropractic.

[CR141] Herzog W, Scheele D, Conway PJ (1999). Electromyographic responses of back and limb muscles associated with spinal manipulative therapy. Spine.

[CR142] Hides JA, Stokes MJ, Saide M, Jull GA, Cooper DH (1994). Evidence of lumbar multifidus muscle wasting ipsilateral to symptoms in patients with acute/subacute low back pain. Spine.

[CR143] Hilibrand AS, Carlson GD, Palumbo MA, Jones PK, Bohlman HH (1999). Radiculopathy and myelopathy at segments adjacent to the site of a previous anterior cervical arthrodesis. J Bone Joint Surg Am.

[CR144] Hillermann B, Gomes AN, Korporaal C, Jackson D (2006). A pilot study comparing the effects of spinal manipulative therapy with those of extra-spinal manipulative therapy on quadriceps muscle strength. J Manipulative Physiol Ther.

[CR145] Hodges PW (2001). Changes in motor planning of feedforward postural responses of the trunk muscles in low back pain. Exp Brain Res.

[CR146] Hodges P, Richardson C (1996). Inefficient muscular stabilisation of the lumbar spine associated with low back pain: a motor control evaluation of transversus abdominis. Spine.

[CR147] Hodges PW, Richardson CA (1999). Altered trunk muscle recruitment in people with low back pain with upper limb movement at different speeds. Arch Phys Med Rehabil.

[CR148] Hodges PW, Tucker K (2011). Moving differently in pain: a new theory to explain the adaptation to pain. Pain.

[CR149] Hodges PW, Lorimer Moseley G (2003). Pain and motor control of the lumbopelvic region: effect and possible mechanisms. J Electromyogr Kinesiol.

[CR150] Hodges P, Holm AK, Hansson T, Holm S (2006). Rapid atrophy of the lumbar multifidus follows experimental disc or nerve root injury. Spine.

[CR151] Hodges PW, Galea MP, Holm S, Holm AK (2009). Corticomotor excitability of back muscles is affected by intervertebral disc lesion in pigs. Eur J Neurosci.

[CR152] Hodges PW, James G, Blomster L, Hall L, Schmid AB, Shu C, Little C, Melrose J (2014). Can proinflammatory cytokine gene expression explain multifidus muscle fiber changes after an intervertebral disc lesion?. Spine.

[CR153] Hodges PW, James G, Blomster L, Hall L, Schmid A, Shu C, Little C, Melrose J (2015). Multifidus muscle changes after back injury are characterized by structural remodeling of muscle, adipose and connective tissue, but not muscle atrophy: molecular and morphological evidence. Spine.

[CR154] Hodges PW, Barbe MF, Loggia ML, Nijs J, Stone LS (2019). Diverse role of biological plasticity in low back pain and its impact on sensorimotor control of the spine. J Orthopaedic Sports Phys Therapy.

[CR155] Holm S, Indahl A, Solomonow M (2002). Sensorimotor control of the spine. J Electromyogr Kinesiol.

[CR156] Holt KR (2014) Effectiveness of chiropractic care in improving sensorimotor function associated with falls risk in older people. University of Auckland 10.1016/j.jmpt.2016.02.003 27050038

[CR157] Holt K, Haavik H, Murphy B, Chi Lun Lee A, Elley CR (2016). Effectiveness of chiropractic care to improve sensorimotor function associated with falls risk in older people: a randomized controlled trial. J Manipulative Physiol Ther.

[CR158] Holt KR, Haavik H, Lee AC, Murphy B, Elley CR (2016b) Effectiveness of chiropractic care to improve sensorimotor function associated with falls risk in older people: a randomized controlled trial. J Manipulative Physiol Ther 10.1016/j.jmpt.2016.02.003 27050038

[CR159] Holt K, Niazi IK, Nedergaard RW, Duehr J, Amjad I, Shafique M, Anwar MN, Ndetan H, Turker KS, Haavik H (2019). The effects of a single session of chiropractic care on strength, cortical drive, and spinal excitability in stroke patients. Sci Rep.

[CR160] Howe JF, Loeser JD, Calvin WH (1977). Mechanosensitivity of dorsal root ganglia and chronically injured axons: a physiological basis for the radicular pain of nerve root compression. Pain.

[CR161] Hubbard RD, Winkelstein BA (2008). Dorsal root compression produces myelinated axonal degeneration near the biomechanical thresholds for mechanical behavioral hypersensitivity. Exp Neurol.

[CR162] Humphries KM, Ward J, Coats J, Nobert J, Amonette W, Dyess S (2013). Immediate effects of lower cervical spine manipulation on handgrip strength and free-throw accuracy of asymptomatic basketball players: a pilot study. J Chiropr Med.

[CR163] Ilic TV, Meintzschel F, Cleff U, Ruge D, Kessler KR, Ziemann U (2002). Short-interval paired-pulse inhibition and facilitation of human motor cortex: the dimension of stimulus intensity. J Physiol.

[CR164] Inghilleri M, Berardelli A, Cruccu G, Manfredi M (1993). Silent period evoked by transcranial stimulation of the human cortex and cervicomedullary junction. J Physiol.

[CR165] James G, Blomster L, Hall L, Schmid AB, Shu CC, Little CB, Melrose J, Hodges PW (2016). Mesenchymal stem cell treatment of intervertebral disc lesion prevents fatty infiltration and fibrosis of the multifidus muscle, but not cytokine and muscle fiber changes. Spine.

[CR166] James G, Sluka KA, Blomster L, Hall L, Schmid AB, Shu CC, Little CB, Melrose J, Hodges PW (2018). Macrophage polarization contributes to local inflammation and structural change in the multifidus muscle after intervertebral disc injury. Eur Spine J.

[CR167] Johnson EO, Babis GC, Soultanis KC, Soucacos PN (2008). Functional neuroanatomy of proprioception. J Surg Orthop Adv.

[CR168] Julkunen P, Säisänen L, Danner N, Niskanen E, Hukkanen T, Mervaala E, Könönen M (2009). Comparison of navigated and non-navigated transcranial magnetic stimulation for motor cortex mapping, motor threshold and motor evoked potentials. Neuroimage.

[CR169] Jull GA, Richardson CA (2000). Motor control problems in patients with spinal pain: a new direction for therapeutic exercise. J Manipulative Physiol Ther.

[CR170] Kang Y-M, Choi W-S, Pickar JG (2002). Electrophysiologic evidence for an intersegmental reflex pathway between lumbar paraspinal tissues. Spine.

[CR171] Kassab R, Alexandre F (2015) Integration of exteroceptive and interoceptive information within the hippocampus: a computational study. Front Syst Neurosci 9 10.3389/fnsys.2015.00087 PMC4456570 26097448

[CR172] Keating L, Lubke C, Powell V, Young T, Souvlis T, Jull G (2001). Mid-thoracic tenderness: a comparison of pressure pain threshold between spinal regions, in asymptomatic subjects. Man Ther.

[CR173] Keller TS, Colloca CJ (2000). Mechanical force spinal manipulation increases trunk muscle strength assessed by electromyography: a comparative clinical trial. J Manipulative Physiol Ther.

[CR174] Kent C (1996). Models of vertebral subluxation: a review. J Vertebral Subluxation Res.

[CR175] Kilteni K, Engeler P, Ehrsson HH (2019) Efference copy is necessary for the attenuation of self-generated touch. bioRxiv 823575 10.1016/j.isci.2020.100843 PMC6997587 32058957

[CR176] Kingett M, Holt K, Niazi IK, Nedergaard RW, Lee M, Haavik H (2019). Increased voluntary activation of the elbow flexors following a single session of spinal manipulation in a subclinical neck pain population. Brain Sci.

[CR177] Klous M, Mikulic P, Latash ML (2011). Two aspects of feedforward postural control: anticipatory postural adjustments and anticipatory synergy adjustments. J Neurophysiol.

[CR178] Kovanur-Sampath K, Mani R, Cotter J, Gisselman AS, Tumilty S (2017). Changes in biochemical markers following spinal manipulation-a systematic review and meta-analysis. Musculoskelet Sci Practice.

[CR179] Kujirai T, Caramia MD, Rothwell JC, Day BL, Thompson PD, Ferbert A, Wroe S, Asselman P, Marsden CD (1993). Corticocortical inhibition in human motor cortex. J Physiol.

[CR180] Kukowski B, Haug B (1992). Quantitative evaluation of the silent period, evoked by transcranial magnetic stimulation during sustained muscle contraction, in normal man and in patients with stroke. Electromyogr Clin Neurophysiol.

[CR181] Kulkarni V, Chandy MJ, Babu KS (2001) Quantitative study of muscle spindles in suboccipital muscles of human foetuses. In Neurol India, 355–9 11799407

[CR182] Lantz CA (1989). The vertebral subluxation complex part 1: an introduction to the model and kinesiological component. Chiro Res J.

[CR183] Le B, Davidson B, Solomonow D, Zhou BH, Yun Lu, Patel V, Solomonow M (2009). Neuromuscular control of lumbar instability following static work of various loads. Muscle Nerve.

[CR184] Leach RA (1986) Manipulation terminology in the chiropractic, osteopathic, and medical literature. In: The chiropractic theories: a synopsis of scientific research. Williams and Wilkins: Baltimore

[CR185] Leach RA (2004). The chiropractic theories: a textbook of scientific research.

[CR186] Lelic D, Niazi IK, Holt K, Jochumsen M, Dremstrup K, Yielder P, Murphy B, Drewes AM, Haavik H (2016). Manipulation of dysfunctional spinal joints affects sensorimotor integration in the prefrontal cortex: a brain source localization study. Neural Plast.

[CR187] Leung A, Gregory NS, Allen LA, Sluka KA (2016). Regular physical activity prevents chronic pain by altering resident muscle macrophage phenotype and increasing interleukin-10 in mice. Pain.

[CR188] Lo CN, Ng J, Au CK, Lim ECW (2019). The effectiveness of spinal manipulation in increasing muscle strength in healthy individuals: a systematic review and meta-analysis. J Manipulative Physiol Ther.

[CR189] Loeb GE, Brown IE, Cheng EJ (1999). A hierarchical foundation for models of sensorimotor control. Exp Brain Res.

[CR190] Lolignier S, Eijkelkamp N, Wood JN (2015). Mechanical allodynia. Pflugers Arch.

[CR191] Lotz M, Vaughan JH, Carson DA (1988). Effect of neuropeptides on production of inflammatory cytokines by human monocytes. Science.

[CR192] Lucci G, Pazzaglia M (2015). Towards multiple interactions of inner and outer sensations in corporeal awareness. Front Hum Neurosci.

[CR193] Lund JP, Donga R, Widmer CG, Stohler CS (1991). The pain-adaptation model: a discussion of the relationship between chronic musculoskeletal pain and motor activity. Can J Physiol Pharmacol.

[CR194] MacDonald DA, Moseley GL, Hodges PW (2006). The lumbar multifidus: does the evidence support clinical beliefs?. Man Ther.

[CR195] MacDonald D, Moseley GL, Hodges PW (2009). Why do some patients keep hurting their back? Evidence of ongoing back muscle dysfunction during remission from recurrent back pain. Pain.

[CR196] Mann CJ, Perdiguero E, Kharraz Y, Aguilar S, Pessina P, Serrano AL, Munoz-Canoves P (2011). Aberrant repair and fibrosis development in skeletal muscle. Skelet Muscle.

[CR197] Marras WS, Davis KG, Heaney CA, Maronitis AB, Allread WG (2000). The influence of psychosocial stress, gender, and personality on mechanical loading of the lumbar spine. Spine.

[CR198] Marshall P, Murphy B (2006). The effect of sacroiliac joint manipulation on feed-forward activation times of the deep abdominal musculature. J Manipulative Physiol Ther.

[CR199] Marshall PWM, Murphy BA (2008). Muscle activation changes following exercise rehabilitation for chronic low back pain. Arch Phys Med Rehabil.

[CR200] McMorland G, Suter E, Casha S, Du Plessis SJ, Hurlbert RJ (2010). Manipulation or microdiskectomy for sciatica A prospective randomized clinical study. J Manipulative Physiol Ther.

[CR201] Mehta RK, Rhee J (2017). Age-specific neural strategies to maintain motor performance after an acute social stress bout. Exp Brain Res.

[CR202] Meier ML, Vrana A, Schweinhardt P (2018) Low back pain: the potential contribution of supraspinal motor control and proprioception. The Neuroscientist. 1073858418809074 10.1177/1073858418809074 PMC6900582 30387689

[CR203] Merton PA, Morton HB (1980). Stimulation of the cerebral cortex in the intact human subject. Nature.

[CR204] Metwali A, Blum AM, Elliott DE, Setiawan T, Weinstock JV (2004). Cutting edge: hemokinin has substance P-like function and expression in inflammation. J Immunol.

[CR205] Moench KM, Wellman CL (2015). Review article: Stress-induced alterations in prefrontal dendritic spines: implications for post-traumatic stress disorder. Neurosci Lett.

[CR206] Morishita Y, Hida S, Naito M, Arimizu J, Matsushima U, Nakamura A (2006). Measurement of the local pressure of the intervertebral foramen and the electrophysiologic values of the spinal nerve roots in the vertebral foramen. Spine.

[CR207] Moseley GL (2003). A pain neuromatrix approach to patients with chronic pain. Man Ther.

[CR208] Moseley GL, Nicholas MK, Hodges PW (2004). Pain differs from non-painful attention-demanding or stressful tasks in its effect on postural control patterns of trunk muscles. Exp Brain Res.

[CR209] Muellbacher W, Facchini S, Boroojerdi B, Hallett M (2000). Changes in motor cortex excitability during ipsilateral hand muscle activation in humans. Clin Neurophysiol.

[CR210] Mulla A, Buckingham JC (1999). Regulation of the hypothalamo–pituitary–adrenal axis by cytokines. Best Pract Res Clin Endocrinol Metab.

[CR211] Nava E, Röder B (2011) Adaptation and maladaptation: insights from brain plasticity. In Progress in brain research (Elsevier) 10.1016/B978-0-444-53752-2.00005-9 21741552

[CR212] Nelson C (1997). The subluxation question. J Chiropr Humanit.

[CR213] Niazi IK, Türker KS, Flavel S, Kinget M, Duehr J, Haavik H (2015). Changes in H-reflex and V-waves following spinal manipulation. Exp Brain Res.

[CR214] Niazi IK, Kamavuako EN, Holt K, Janjua TA, Kumari N, Amjad I, Haavik H (2020) The effect of spinal manipulation on the electrophysiological and metabolic properties of the tibialis anterior muscle. Healthcare 8 10.3390/healthcare8040548 PMC7764559 33321904

[CR215] Nougarou F, Dugas C, Deslauriers C, Pagé I, Descarreaux M (2013). Physiological responses to spinal manipulation therapy: investigation of the relationship between electromyographic responses and peak force. J Manipulative Physiol Ther.

[CR216] Organization World Health (2005) WHO guidelines on basic training and safety in chiropractic

[CR217] Özyurt MG, Haavik H, Nedergaard RW, Topkara B, Şenocak BS, Göztepe MB, Niazi IK, Türker KS (2019). Transcranial magnetic stimulation induced early silent period and rebound activity re-examined. PLoS ONE.

[CR218] Pagé I, Nougarou F, Dugas C, Descarreaux M (2014). The effect of spinal manipulation impulse duration on spine neuromechanical responses. J Can Chiropr Assoc.

[CR012] Pagé I, Nougarou F, Descarreaux M (2016) Neuromuscular response amplitude to mechanical stimulation using large-array surface electromyography in participants with and without chronic low back pain. J Electromyogr Kinesiol 27:2–-29. 10.1016/j.jelekin.2016.01.004 10.1016/j.jelekin.2016.01.004 26874078

[CR219] Palmer DD (1910). Text-book of the science, art and philosophy of chiropractic.

[CR220] Palmgren PJ, Sandstrom PJ, Lundqvist FJ, Heikkila H (2006). Improvement after chiropractic care in cervicocephalic kinesthetic sensibility and subjective pain intensity in patients with nontraumatic chronic neck pain. J Manipulative Physiol Ther.

[CR221] Palmgren PJ, Lindeberg A, Nath S, Heikkila H (2009). Head repositioning accuracy and posturography related to cervical facet nerve blockage and spinal manipulative therapy in healthy volunteers: a time series study. J Manipulative Physiol Ther.

[CR222] Park K-N, Kwon O-Y, Kim S-J, Kim S-H (2017). Asymmetry of neck motion and activation of the cervical paraspinal muscles during prone neck extension in subjects with unilateral posterior neck pain. J Back Musculoskelet Rehabil.

[CR223] Passatore M, Roatta S (2006). Influence of sympathetic nervous system on sensorimotor function: whiplash associated disorders (WAD) as a model. Eur J Appl Physiol.

[CR224] Paulus I, Brumagne S (2008). Altered interpretation of neck proprioceptive signals in persons with subclinical recurrent neck pain. J Rehabil Med.

[CR225] Pearce BD, Biron CA, Miller AH (2001) Neuroendocrine-immune interactions during viral infections. In Advances in Virus Research (Academic Press). 10.1016/s0065-3527(01)56036-4 11450310

[CR226] Pedler A, McMahon K, Galloway G, Durbridge G, Sterling M (2018). Intramuscular fat is present in cervical multifidus but not soleus in patients with chronic whiplash associated disorders. PLoS ONE.

[CR227] Pera Le, Domenica T-N, Valeriani M, Oliviero A, Di Lazzaro V, Tonali PA, Arendt-Nielsen L (2001). Inhibition of motor system excitability at cortical and spinal level by tonic muscle pain. Clin Neurophysiol.

[CR228] Pickar JG (2002). Neurophysiological effects of spinal manipulation. Spine J.

[CR229] Pickar JG, Bolton PS (2012). Spinal manipulative therapy and somatosensory activation. J Electromyogr Kinesiol.

[CR230] Pickar JG, Kang YM (2006). Paraspinal muscle spindle responses to the duration of a spinal manipulation under force control. J Manipulative Physiol Ther.

[CR231] Pickar JG, Wheeler JD (2001). Response of muscle proprioceptors to spinal manipulative-like loads in the anesthetized cat. JMPT.

[CR232] Pickar JG, Sung PS, Kang YM, Ge W (2007). Response of lumbar paraspinal muscles spindles is greater to spinal manipulative loading compared with slower loading under length control. Spine J.

[CR233] Piscitelli D, Falaki A, Solnik S, Latash ML (2017). Anticipatory postural adjustments and anticipatory synergy adjustments: preparing to a postural perturbation with predictable and unpredictable direction. Exp Brain Res.

[CR234] Plaza-Manzano G, Molina F, Lomas-Vega R, Martínez-Amat A, Achalandabaso A, Hita-Contreras F (2014). Changes in biochemical markers of pain perception and stress response after spinal manipulation. J Orthopaedic Sports Phys Therapy.

[CR235] Potter L, McCarthy C, Oldham J (2013). Physiological effects of spinal manipulation: a review of proposed theories. Phys Therapy Rev.

[CR236] Practice Guidelines for Straight Chiropractic. In. 1992. *Wyndham Conference*. Chandler, Arizona

[CR237] Proske U, Gandevia SC (2012). The proprioceptive senses: their roles in signaling body shape, body position and movement, and muscle force. Physiol Rev.

[CR238] Quadt L, Critchley HD, Garfinkel SN (2018). The neurobiology of interoception in health and disease. Ann N Y Acad Sci.

[CR239] Radebold A, Cholewicki J, Panjabi MM, Patel TC (2000). Muscle response pattern to sudden trunk loading in healthy individuals and in patients with chronic low back pain. Spine.

[CR240] Reed WR, Pickar JG (2015). Paraspinal muscle spindle response to intervertebral fixation and segmental thrust level during spinal manipulation in an animal model. Spine.

[CR241] Reed WR, Long CR, Pickar JG (2013). Effects of unilateral facet fixation and facetectomy on muscle spindle responsiveness during simulated spinal manipulation in an animal model. J Manipulative Physiol Ther.

[CR242] Reed WR, Dong-Yuan Cao, CR Long, GN Kawchuk, JG Pickar (2013b) Relationship between biomechanical characteristics of spinal manipulation and neural responses in an animal model: effect of linear control of thrust displacement versus force, thrust amplitude, thrust duration, and thrust rate. Evidence-based complementary and alternative Medicine 2013 10.1155/2013/492039 PMC3563165 23401713

[CR013] Reed WR, Sozio R, Pickar JG, Onifer SM (2014) Effect of spinal manipulation thrust duration on trunk mechanical activation thresholds of nociceptive-specific lateral thalamic neurons. J Manipulative Physiol Ther 37:552–560. 10.1016/j.jmpt.2014.08.006 10.1016/j.jmpt.2014.08.006 PMC4394198 25220757

[CR243] Reed WR, Long CR, Kawchuk GN, Pickar JG (2014). Neural responses to the mechanical parameters of a high-velocity, low-amplitude spinal manipulation: effect of preload parameters. J Manipulative Physiol Ther.

[CR244] Reed WR, Pickar JG, Sozio RS, Long CR (2014). Effect of spinal manipulation thrust magnitude on trunk mechanical activation thresholds of lateral thalamic neurons. J Manipulative Physiol Ther.

[CR245] Reed WR, Long CR, Kawchuk GN, Pickar JG (2015). Neural responses to the mechanical characteristics of high velocity, low amplitude spinal manipulation: effect of specific contact site. Man Ther.

[CR246] Reed WR, Cranston JT, Onifer SM, Little JW, Sozio RS (2017). Decreased spontaneous activity and altered evoked nociceptive response of rat thalamic submedius neurons to lumbar vertebra thrust. Exp Brain Res.

[CR247] Reed WR, Pickar JG, Sozio RS, Liebschner MAK, Little JW, Gudavalli MR (2017). Characteristics of Paraspinal muscle spindle response to mechanically assisted spinal manipulation: a preliminary report. J Manipulative Physiol Ther.

[CR248] Rodine RJ, Vernon H (2012). Cervical radiculopathy: a systematic review on treatment by spinal manipulation and measurement with the Neck Disability Index. J Can Chiropr Assoc.

[CR249] Rogers RG (1997). The effects of spinal manipulation on cervical kinesthesia in patients with chronic neck pain: a pilot study. J Manipulative Physiol Ther.

[CR250] Rosner AL (2016). Chiropractic identity: a neurological, professional, and political assessment. J Chiropractic Humanities.

[CR251] Rossini PM, Rossini PM, Mauguière F (1990). Methodological and physiological aspects of motor evoked potentials. New trends in clinical neurophysiology (EEG Suppl 41).

[CR252] Rothwell JC (1997). Techniques and mechanisms of action of transcranial stimulation of the human motor cortex. J Neurosci Methods.

[CR253] Sanders GD, Nitz AJ, Abel MG, Symons TB, Shapiro R, Black WS, Yates JW (2015). Effects of lumbosacral manipulation on isokinetic strength of the knee extensors and flexors in healthy subjects: a randomized controlled, single-blind crossover trial. J Chiropr Med.

[CR254] Santos MJ, Kanekar N, Aruin AS (2010). The role of anticipatory postural adjustments in compensatory control of posture: 1. Electromyographic analysis. J Electromyogr Kinesiol.

[CR255] Sherrington C (1952) The integrative action of the nervous system (CUP Archive)

[CR256] Silfies SP, Mehta R, Smith SS, Karduna AR (2009). Differences in feedforward trunk muscle activity in subgroups of patients with mechanical low back pain. Arch Phys Med Rehabil.

[CR257] Silverman MN, Pearce BD, Biron CA, Miller AH (2005). Immune modulation of the hypothalamic-pituitary-adrenal (HPA) axis during viral infection. Viral Immunol.

[CR258] Simoneau GG, Ulbrecht JS, Derr JA, Cavanagh PR (1995). Role of somatosensory input in the control of human posture. Gait Posture.

[CR259] Singaravelu SK, Hoheisel U, Mense S, Rolf-Detlef Treede (2021) Rat dorsal horn neurons primed by stress develop a long-lasting manifest sensitization after a short-lasting nociceptive low back input. Pain reports 6 10.1097/PR9.0000000000000904 PMC7935483 33688602

[CR260] Solarino B, Coppola F, Di Vella G, Corsalini M, Quaranta N (2009). Vestibular evoked myogenic potentials (VEMPs) in whiplash injury: a prospective study. Acta Otolaryngol.

[CR261] Song X-J, Dong-Sheng Xu, Vizcarra C, Rupert RL (2003). Onset and recovery of hyperalgesia and hyperexcitability of sensory neurons following intervertebral foramen volume reduction and restoration. J Manipulative Physiol Ther.

[CR262] Song XJ, Gan Q, Cao JL, Wang ZB, Rupert RL (2006). Spinal manipulation reduces pain and hyperalgesia after lumbar intervertebral foramen inflammation in the rat. J Manipulative Physiol Ther.

[CR263] Song XJ, Huang ZJ, Song WB, Song XS, Fuhr AF, Rosner AL, Ndtan H, Rupert RL (2016). Attenuation effect of spinal manipulation on neuropathic and postoperative pain through activating endogenous anti-inflammatory cytokine interleukin 10 in rat spinal cord. J Manipulative Physiol Ther.

[CR264] Stanton TR, Leake HB, Chalmers KJ, Moseley GL (2016). Evidence of impaired proprioception in chronic, idiopathic neck pain: systematic review and meta-analysis. Phys Ther.

[CR265] Stephensen RW (1927). Chiropractic Text-book.

[CR266] Stubbs M, Harris M, Solomonow M, Zhou B, Lu Y, Baratta RV (1998). Ligamento-muscular protective reflex in the lumbar spine of the feline. J Electromyogr Kinesiol.

[CR267] Suffredini AF, Fantuzzi G, Badolato R, Oppenheim JJ, O'Grady NP (1999). New insights into the biology of the acute phase response. J Clin Immunol.

[CR268] Sung PS, Kang YM, Pickar JG (2005). Effect of spinal manipulation duration on low threshold mechanoreceptors in lumbar paraspinal muscles: a preliminary report. Spine.

[CR269] Suter E, McMorland G, Herzog W, Bray R (1999). Decrease in quadriceps inhibition after sacroiliac joint manipulation in patients with anterior knee pain. J Manipulative Physiol Ther.

[CR270] Tagliabue M, McIntyre J (2014). A modular theory of multisensory integration for motor control. Front Comput Neurosci.

[CR271] Taylor HH, Holt K, Murphy B (2010) Exploring the neuromodulatory effects of the vertebral subluxation and chiropractic care. Chiropractic J Australia 40

[CR272] Teodorczyk-Injeyan JA, McGregor M, Triano JJ, Injeyan SH (2018). Elevated production of nociceptive CC chemokines and sE-selectin in patients with low back pain and the effects of spinal manipulation: a nonrandomized clinical trial. Clin J Pain.

[CR273] Thayer JF (2009). Vagal tone and the inflammatory reflex. Cleve Clin J Med.

[CR274] The Rubicon Group (2017) Definition and position statement on the chiropractic subluxation

[CR275] Thunberg J, Hellström F, Sjölander P, Bergenheim M, Wenngren B-I, Johansson H (2001). Influences on the fusimotor-muscle spindle system from chemosensitive nerve endings in cervical facet joints in the cat: possible implications for whiplash induced disorders. Pain.

[CR276] Tokimura H, Ridding MC, Tokimura Y, Amassian VE, Rothwell JC (1996). Short latency facilitation between pairs of threshold magnetic stimuli applied to human motor cortex. Electroencephalogr Clin Neurophysiol.

[CR277] Tong MH, Mousavi SJ, Kiers H, Ferreira P, Refshauge K, van Dieën J (2017). Is there a relationship between lumbar proprioception and low back pain? A systematic review with meta-analysis. Arch Phys Med Rehabil.

[CR278] Treleaven J (2008). Sensorimotor disturbances in neck disorders affecting postural stability, head and eye movement control. Man Ther.

[CR279] Treleaven J (2017). Dizziness, unsteadiness, visual disturbances, and sensorimotor control in traumatic neck pain. J Orthop Sports Phys Ther.

[CR280] Tresch MC, Saltiel P, d’Avella A, Bizzi E (2002). Coordination and localization in spinal motor systems. Brain Res Rev.

[CR281] Triano JJ, Budgell B, Bagnulo A, Roffey B, Bergmann T, Cooperstein R, Gleberzon B, Good C, Perron J, Tepe R (2013). Review of methods used by chiropractors to determine the site for applying manipulation. Chiropractic Manual Therapies.

[CR282] Tucker KJ, Tuncer M, Türker KS (2005). A review of the H-reflex and M-wave in the human triceps surae. Hum Mov Sci.

[CR283] Turker KS, Cheng HB (1994). Motor-unit firing frequency can be used for the estimation of synaptic potentials in human motoneurones. J Neurosci Methods.

[CR284] Turker KS, Powers RK (2005). Black box revisited: a technique for estimating postsynaptic potentials in neurons. Trends Neurosci.

[CR285] Van Dieën JH, Reeves NP, Kawchuk G, Van Dillen LR, Hodges PW (2018) Motor control changes in low-back pain: divergence in presentations and mechanisms. J Orthopaedic Sports Phys Therapy 1–24 10.2519/jospt.2019.7917 PMC7393576 29895230

[CR286] Van Dieën JH, Reeves NP, Kawchuk G, Van Dillen LR, Hodges PW (2019). Analysis of motor control in patients with low back pain: a key to personalized care. J Orthopaedic Sports Phys Therapy.

[CR287] van Vliet PM, Heneghan NR (2006) Motor control and the management of musculoskeletal dysfunction. *Manual Therapy Conference Proceedings from the 2nd International Conference on Movement Dysfunction. Pain and Performance: Evidence and Effect* 11: 208–13 10.1016/j.math.2006.03.009 16781184

[CR288] Vila-Chã C, Falla D, Correia MV, Farina D (2012). Changes in H reflex and V wave following short-term endurance and strength training. J Appl Physiol.

[CR289] Villalta SA, Nguyen HX, Deng B, Gotoh T, Tidball JG (2009). Shifts in macrophage phenotypes and macrophage competition for arginine metabolism affect the severity of muscle pathology in muscular dystrophy. Hum Mol Genet.

[CR290] Vining R, Long CR, Amy Minkalis M, Gudavalli R, Xia T, Walter J, Coulter I, Goertz CM (2020). Effects of chiropractic care on strength, balance, and endurance in active-duty us military personnel with low back pain: a randomized controlled trial. J Altern Complement Med.

[CR291] Wehling-Henricks M, Jordan MC, Gotoh T, Grody WW, Roos KP, Tidball JG (2010). Arginine metabolism by macrophages promotes cardiac and muscle fibrosis in mdx muscular dystrophy. PLoS ONE.

[CR292] WHO (2016) ICD-ICD-10-CM-International Classification of Diseases, Tenth Revision, Clinical Modification.(2016). In.

[CR293] Wilke J, Schleip R, Yucesoy CA, Banzer W (2018). Not merely a protective packing organ? A review of fascia and its force transmission capacity. J Appl Physiol.

[CR294] Wilson SA, Lockwood GW, Thickbroom GW, Mastaglia FL (1993). The muscle silent period following transcranial magnetic cortical stimulation. J Neurol Sci.

[CR295] Windhorst U (2007). Muscle proprioceptive feedback and spinal networks. Brain Res Bull.

[CR296] Wirth B, Gassner A, de Bruin ED, Axén I, Swanenburg J, Humphreys BK, Schweinhardt P (2019). Neurophysiological effects of high velocity and low amplitude spinal manipulation in symptomatic and asymptomatic humans: a systematic literature review. Spine.

[CR297] Woo S-H, Lukacs V, De Nooij JC, Zaytseva D, Criddle CR, Francisco A, Jessell TM, Wilkinson KA, Patapoutian A (2015). Piezo2 is the principal mechanotransduction channel for proprioception. Nat Neurosci.

[CR298] Yahia L, Rhalmi S, Newman N, Isler M (1992). Sensory innervation of human thoracolumbar fascia: an immunohistochemical study. Acta Orthop Scand.

[CR299] Yiou E, Caderby T, Hussein T (2012). Adaptability of anticipatory postural adjustments associated with voluntary movement. World J Orthopedics.

[CR300] Zhao WP, Kawaguchi Y, Matsui H, Kanamori M, Kimura T (2000). Histochemistry and morphology of the multifidus muscle in lumbar disc herniation: comparative study between diseased and normal sides. Spine.

[CR301] Ziemann U, Tergau F, Wassermann EM, Wischer S, Hildebrandt J, Paulus W (1998). Demonstration of facilitatory I wave interaction in the human motor cortex by paired transcranial magnetic stimulation. J Physiol.

